# The combined effects of predation, fishing, and ocean productivity on salmon species targeted by marine mammals in the northeast Pacific

**DOI:** 10.1371/journal.pone.0296358

**Published:** 2024-03-14

**Authors:** Fanny Couture, Villy Christensen, Carl Walters

**Affiliations:** 1 Institute for the Oceans and Fisheries, University of British Columbia, Vancouver, British Columbia, Canada; 2 Marine Mammals Research Program, Ocean Wise Research Institute, Vancouver, British Columbia, Canada; Swedish University of Agricultural Sciences and Swedish Institute for the Marine Environment, University of Gothenburg, SWEDEN

## Abstract

Along the northeast Pacific coast, the salmon-eating southern *resident* killer whale population (SRKW, *Orcinus orca*) have been at very low levels since the 1970s. Previous research have suggested that reduction in food availability, especially of Chinook salmon (*Oncorhynchus tshawytscha*), could be the main limiting factor for the SRKW population. Using the ecosystem modelling platform Ecopath with Ecosim (EwE), this study evaluated if the decline of the Pacific salmon populations between 1979 and 2020 may have been impacted by a combination of factors, including marine mammal predation, fishing activities, and climatic patterns. We found that the total mortality of most Chinook salmon populations has been relatively stable for all mature returning fish despite strong reduction in fishing mortality since the 1990s. This mortality pattern was mainly driven by pinnipeds, with increases in predation between 1979 and 2020 mortality ranging by factors of 1.8 to 8.5 across the different Chinook salmon population groups. The predation mortality on fall-run Chinook salmon smolts originating from the Salish Sea increased 4.6 times from 1979 to 2020, whereas the predation mortality on coho salmon (*Oncorhynchus kisutch*) smolts increased by a factor of 7.3. The model also revealed that the north Pacific gyre oscillation (NPGO) was the most important large-scale climatic index affecting the stock productivity of Chinook salmon populations from California to northern British Columbia. Overall, the model provided evidence that multiple factors may have affected Chinook salmon populations between 1979 and 2020, and suggested that predation mortality by marine mammals could be an important driver of salmon population declines during that time.

## Introduction

The Southern *resident* Killer Whale (SRKW, *Orcinus orca*) population inhabits the coastal waters between northern British Columbia and central California [1]. SRKW are highly specialized predators, feeding primarily on Pacific salmon species [2–4]. Chinook salmon (*Oncorhynchus tshawytscha*) is considered a prey of choice for SRKW during the summer months in the Salish Sea, where they can constitute up to 90% of the diet [3]. The SRKW population has been at a very low level since its identification as a distinct ecotype in the early 1970s, and was listed as *Endangered* by the Committee on the Status of Endangered Wildlife in Canada (COSEWIC) in 2001, and under the Endangered Species Act (ESA) in the US in 2005 [1, 5]. As of July 2022, the population only counted 73 individuals, and the abundance, quality, and availability of prey have been designated as major drivers of the SRKW population trend over the last 50 years [6–8]. The question of the potential causes behind the low levels of the SRKW population is of critical importance, as another salmon-eating *resident* killer whale population (i.e. the Northern *resident* Killer Whale, NRKW, *Orcinus orca*) geographically overlaps with the SRKW [1]. Although largely feeding on Chinook salmon as well, the NRKW population has more than doubled since 1975, and scientists are still baffled on what could explain the different demographic trends of those two populations [1].

Most Chinook salmon populations along the northeast Pacific have decreased in abundance over recent decades, and an ongoing debate exists regarding the causes of these declines. While some studies [9–11] argue that the growing pinniped populations now have a substantial predation impact on those salmon populations, others suggest that fishing [12], climatic patterns [13–16], and deterioration of freshwater habitats [17, 18] could have significantly impacted the productivity of Chinook salmon populations over the years.

Several pinniped species occur along the northeast Pacific coast, including harbor seals (*Phoca vitulina*) and Steller (*Eumetopias jubatus*) and California (*Zalophus californianus*) sea lions [19–22]. All three species had been intensely depleted by commercial harvests and predator control programs in Canada and the US prior to their protection under the *Fisheries Act* in Canada in 1970 and the *Marine Mammal Protection Act* in the U.S. 1972 [23]. All populations have grown in size since then, with the harbor seal population stabilizing in the early 2000s throughout its range [20, 21, 24]. In the Strait of Georgia only, the harbor seal population has increased by about 20-fold between 1970 and the early 2000s [20]. Further, the population sizes of Steller and California sea lion have increased about six and four times since the early 1970s, respectively [22, 25]. Although constituting a limited portion of their diet, all three species feed upon Pacific salmonids, including Chinook salmon [11, 26–28].

The potential impact of increased predation pressure on depleted Pacific salmon populations by harbor seal and sea lion populations has received considerable attention in recent years. Chasco et al. (2017) estimated that the annual biomass of Chinook salmon consumed by pinnipeds along the northeast Pacific coast increased by almost ten-fold between 1970 and 2015 [10]. In 2015, that consumption would have been equivalent to that of all *resident* killer whales, and two times higher than all fisheries combined [10]. In the Columbia River only, Sorel et al. (2020) established a negative correlation between Chinook salmon survival and pinniped abundance, with overall mortality increasing by about 21.1% between years of low and high California sea lion abundance [29]. Despite those findings, there is still a real need to understand whether this growing predation pressure on Pacific salmon species over the last 40 years could have significantly impacted the overall availability of prey available to SRKW, ultimately limiting the growth of the population.

Climatic patterns have also been advocated as being an important driver for salmon population declines along the northeast Pacific coast. Crozier et al. (2008, 2021) suggested that changes in streamflow and temperatures associated with climate change might pose a direct threat to Chinook salmon survival during their freshwater stages [16, 30]. For instance, high temperature might reduce stream flow, hence potentially decreasing potential habitat size and further increasing food competition and vulnerability to predation [30]. Several large scale seasonal and annual environmental indicators patterns, such as the Pacific Decadal Oscillation (PDO), North Pacific Gyre Oscillation (NPGO), or El-Niño Southern Oscillation (ENSO), have also been of particular interests for scientists and fisheries managers in recent years. For instance, the NPGO has been shown to correlate with variations in Pacific salmon population productivity [14] and survival [31], while the PDO has been found to be associated with changes in returning spawner abundance [32]. In addition, Kilduff et al. (2015) suggested that the NPGO was the most important driver associated with changes in Chinook and coho salmon survival along the north American west coast since the 1980s [31]. Other studies [33, 34] found similar co-variations between NPGO and other organisms along the northeast Pacific Ocean, including invertebrates, demersal fish, and phytoplankton.

Fishing has been a major cause of Pacific salmon populations’ decline until the late twentieth century. Prior to European contact, Argue et al. (1990) estimated that about 18,000 metric tons of Pacific salmon were consumed annually by aboriginal communities along the coast, which would be equivalent to the fisheries levels observed in the early 2000s [35, 36]. In the early 1800s, salmon were also harvested by fur traders for subsistence and trade [36]. By the mid-1860s, Pacific salmon were considered the cornerstone of the fishing industry of British Columbia [35, 37]. At this time, new fishing techniques, such as trolling, allowed for more intensive and extensive fishing activities [36]. The fishing industry continued to expand over the next decades, with over 7,000 vessels and almost 100 salmon canneries operating in British Columbia by the 1920s [36]. Between 1970 and 1990, more than 1,000,000 Chinook salmon were caught annually along this coast [36]. Intense fishing activity was found to have significant impacts on the abundance, age, and size of returning Chinook salmon individuals [38]. More recently, Hard et al. (2008) showed that high fishing pressure could even induce evolutionary changes (i.e. morphology, migration timing, and maturation timing) on all Pacific salmon species [39]. However, in the face of the dramatic decline of most Chinook salmon stocks, drastic harvesting restrictions (e.g., seasonal closures, gear, licenses) were implemented in the mid-1990s along the northeast Pacific coast [40]. Altogether, recent evidence [9, 41] supports the argument that the current low levels of most Pacific Chinook salmon populations cannot not be solely attributed to fishing pressure. Ward et al. (2013) argued that a fishing reduction could have mainly had a beneficial impact in the early 1980s [12]. Following this claim, O’Farrell and Satterthwaite (2015) showed that the highest impact of fisheries on Chinook salmon in California would also have occurred in the mid-1980s [41]. Besides, despite the reduction in fishing activities over the past 40 years, the overall mortality rates of Chinook salmon along the northeast Pacific have kept increasing, likely driven mainly by predation by marine mammals [9]. In comparison to the findings presented by Chasco et al. (2017), this research will further investigate the impact of those declining Chinook salmon populations for the SRKW. Our study will also provide an additional level of understanding regarding the potential role of primary productivity fluctuations in those salmonid populations.

In this study we use an ecosystem modelling approach to evaluate if the decline of Pacific salmon populations could be due to a combination of factors, rather than to any single factor as evaluated by most previous studies. Choosing an ecosystem-based approach might also allow for developing more realistic and sustainable conservation and management plans for both Pacific salmon and SRKW. This is of significant importance, especially given that both salmon and killer whales are iconic and highly commercially and ecologically valuable species of great conservation concern.

Analytical methods became an important tool for scientists and ecologists to understand animal population dynamics in the 1930s [42]. Ecosystem modelling is increasingly recognized as a convincing method to provide an understanding of how specific perturbations could affect marine ecosystems while predicting future population trends [42, 43]. Such approach provides a global representation of an ecosystem’s mechanisms, as it incorporates species life-history characteristics, harvest data, as well as spatial and temporal ecological variations over time [44, 45].

The ecosystem modelling platform Ecopath with Ecosim (EwE) was ranked the most applied tool for modelling marine and aquatic ecosystems worldwide [46, 47]. The software has been used to address a variety of questions, including fisheries impacts [44, 48, 49], efficiency of marine protected areas [46, 50, 51], habitat loss [52, 53], interactions between marine species [54–58], environmental impact assessments [59–62], pollution [63] as well as climate change and associated environmental variations [64–66]. This modelling approach has been widely applied in the northeast Pacific, with about 37 EwE models created to date for different areas extending from the Aleutian Islands to Mexico [67]. Among those models, some [68, 69] aimed at understanding the bottom-up and top-down processes regulating the ecosystem, while others [70, 71] examined the adequacy of fisheries management plans or intended to provide a general overview of a static system [72, 73]. In this study, the use of such modelling platform would allow to gain valuable insight on the concurrent effects of the three most important factors impacting Pacific salmon species: marine mammal predation, fishing activity, and primary production.

In this paper, we aimed to evaluate to which extent marine mammal predation, fishing activities, and climatic patterns may have affected the population trends of prey available to SRKW between 1979 and 2020. The primary objectives of this paper were to 1) estimate the predicted variations in predation pressure on salmon populations from 1979 to 2020, 2) examine if competition for food might have occurred between the SRKW and other marine mammals during that time, and 3) assess whether environmental variables may have impacted the population trends of prey available to SRKW. The use of the EwE modelling approach will allow to use historical fishing data to drive the fishing pressure on the different Pacific salmon species over the years, while marine mammal diet studies and population demographics will be used as a proxy for marine mammal predation pressure. Ultimately, this research aimed at understanding the ecological linkages existing in the ecosystem while providing a detailed overview of the method and parameters used in an EwE model. This study will fit into the important research effort aiming at conserving economically and culturally valuable Pacific salmon species while ensuring the resilience of an already endangered killer whale population.

## Materials and methods

The ecological modeling platform Ecopath with Ecosim (EwE, http://www.ecopath.org) was used to describe the population dynamics related to the interplay between salmon species, killer whales, pinnipeds, fisheries, and environmental conditions between 1979 and 2020 [74]. EwE combines a static analysis (Ecopath) of an ecosystem trophic mass balance (i.e biomass status and trophic flow) with a temporal (Ecosim) dynamic model for representing variations over time [74].

### Study area

**Fig 1:**
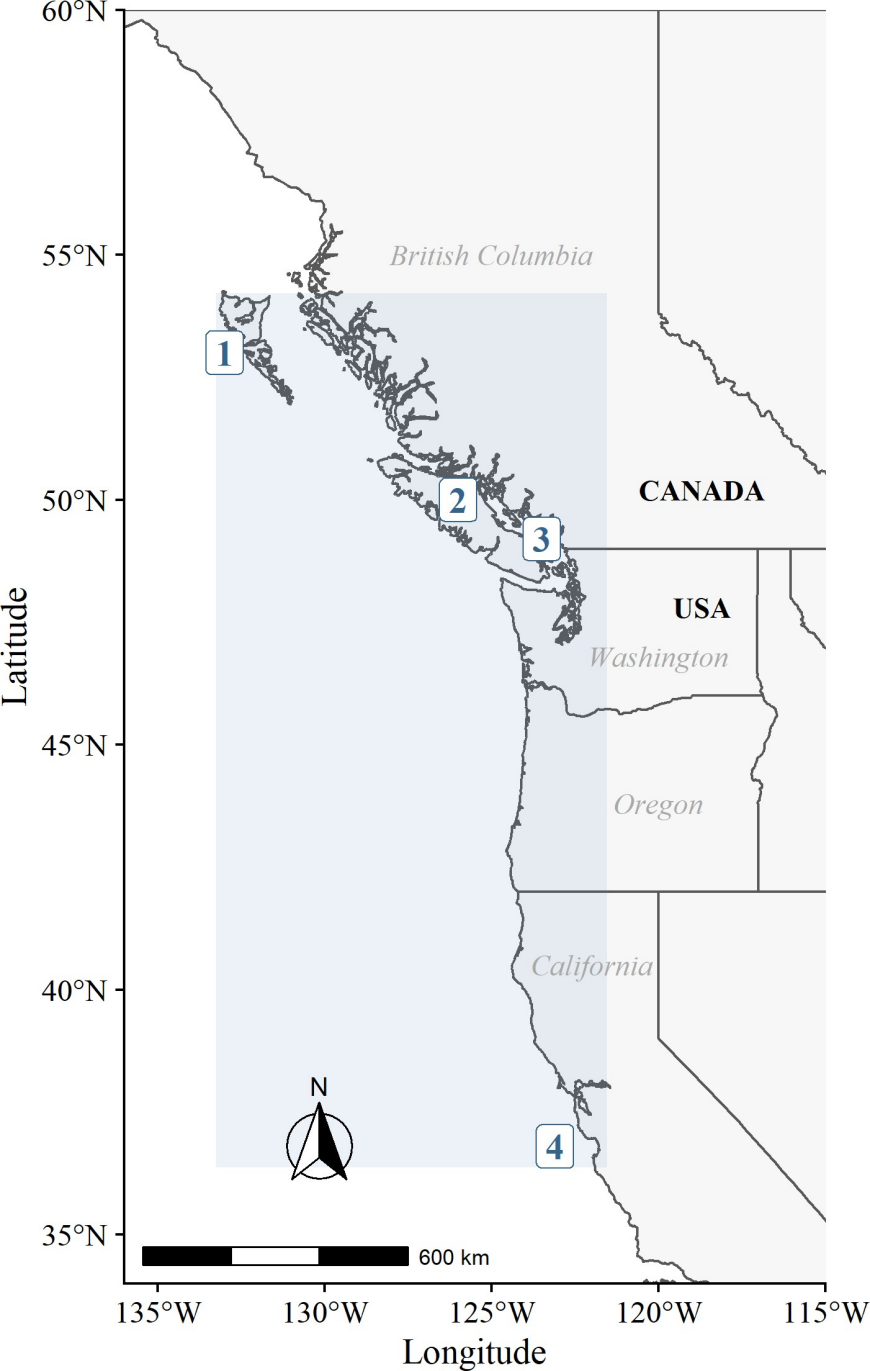
Study area of the model. The study area of the model is the blue square, which corresponds to the historical geographical range of SRKW, and extends from the northern tip of the Haida Gwaii Archipelago (**1**) (central British Columbia, Canada) to Monterey Bay (**4**) in California (USA). The model area includes the Salish Sea (**3**) and the Strait of Georgia, which is open water part of the Salish Sea between Vancouver Island (**2**) and the mainland of British Columbia. This map was created using the R package rnaturalearth.

The study area of the model corresponded to the historical geographical range of SRKW, and expends from the northern tip of the Haida Gwaii Archipelago on the western coast of British Columbia (Canada) to the northern border of California (USA) (Fig 1). It included the continental shelves of British Columbia (~150,000 km^2^), Washington and Oregon State (~ 62,486 km^2^), and the northern portion of California (~30,137 km^2^). The total model area ([133°26’25W, 121°55’42E, 54°22’08N, 36°37’44S]) was estimated at 242,623 km^2^ [69, 75].

### Ecopath model

The Ecopath model was built to represent the base year 1979, and represented the start for the time dynamic Ecosim analysis. The Ecopath analysis provided a static mass-balanced assessment of stocks and flows for the ecosystem (biomass estimates, mortality rates, and trophic flows) [74]. This analysis relies on two master equations used to describe the production term (Eq 1) and the energy flow between each group of an ecosystem (Eq 2) [74]. In addition to diet compositions for all consumer groups (biomass pools), four parameters were provided or estimated for each group prior to running the Ecopath analysis: the biomass (B, t/km^2^), the production/biomass ratio (P/B, (year^-1^)), the consumption/biomass ratio (Q/B, (year^-1^)), and the fisheries landings (Y, t/km^2^/year)). When a population is considered at equilibrium (i.e. stable population level), the production (P/B) represents the instantaneous total mortality rate of a functional group (usually referred to as Z in fisheries science) [76]. In the case of a growing population, the logarithm of the finite rate of increase of the population (usually referred to as λ) needs to be added to the calculation of the P/B ratio, so that P/B = Z + ln (λ). In the case of a continuous population growth rate, ln (λ) is also equal to the intrinsic rate of increase of the population, which can be defined as the maximum instantaneous growth rate that a species can achieve under ideal survival and fecundity conditions. The consumption/biomass ratio Q/B corresponds to the total consumption rate of a predator over its biomass.


$${\left(P/B\right)}_{i}*B_{i}=Y_{i}+DC_{ij}*B_{i}*\sum_{j=1}{\left(Q/B\right)}_{i}+E_{i}+BA_{i}+{\left(P/B\right)}_{i}*B_{i}*(1‐EE_{i})$$ (Eq 1)

$${\left(Q/B\right)}_{i}*B_{i}={\left(P/B\right)}_{i}*B_{i}+R_{i}+UN_{i}$$ (Eq 2)

In Eq 1, DC_*ij*_ * B_*i*_ * ∑_*j = 1*_(Q/B)_*i*_ represents the sum of predation mortalities from predators *j* on a given prey *i* depending on the relative proportion of the diet predator *j* obtained from prey *i* (DC_*ij*_)_._ Y_*i*_ represents the fisheries catch of functional group *i*, E_*i*_ the net migration of group *i*, BA_*i*_ the bioaccumulation of group *i*, and EE_i_ the Ecotrophic Efficiency (1- mortalities, i.e. proportion of the production used in the model) of group *i*. In Eq 2, R_*i*_ corresponds to the respiration rate of group *i*, and UN_*i*_ to the unassimilated food for group *i*.

All the parameters sources used to build the Ecopath model for the year 1979 are presented in details in the Section 1 of S1 Appendix.

#### Functional groups

Altogether, 74 functional groups were included in the model. A functional group can be defined as a species or a group of ecologically or taxonomically related species, or a life history stage or stanza for one species [77]. In total, species (i.e. Chinook salmon, coho salmon, chum salmon, and herring) were split into ‘multi-stanzas’, allowing to capture ontogenetic diet shifts and different age-specific predation and exploitation patterns [77, 78]. ‘Pedigrees’ indicating how well input data were rooted in local information were associated with most parameters entered in Ecopath, allowing to define confidence intervals around data based on their origin [74, 79]. Those pedigree estimates can be used by the Monte-Carlo simulations of the Ecosim routine to calculate confidence intervals in the different Ecopath ecological indicators [78]. All the parameters and literature sources used to build the Ecopath model for the year 1979 are summed up in this paper and presented in Table 1 (marine mammals), Table 2 (salmon species), and Table 3 (all other functional groups). The references listed in those tables are not intended to serve as reference citations. However, all associated references can be found in the S1 Appendix. All parameter estimates are listed in Table 4, whereas detailed descriptions of how the parameters were calculated for the different functional groups can be found in the Section 1 of S1 Appendix.

**Table 1:** Literature sources used to compute the Ecopath parameters of the eight marine mammal functional groups included in the model.

Functional group	Species	Parameter	Literature
TKW	Transient killer whale (*Orcinus orca*)	Population trend, average longevity, distribution	Ford et al. 2007, 2013
		Food consumption	Williams et al. 2004
		Population trend	Center for Whale Research 2022
SRKW	Southern *resident* killer whale (*Orcinus orca*)	Mass-at-age estimates	Noren 2011
		Survival-at-age and average longevity	Robeck 2015
		Diet	Ford and Ellis 2006, Hanson et al. 2010, 2021
		Population growth rate	Olesiuk et al. 1990, Krahn et al. 2002
		Population trend	Center for Whale Research 2022
NRKW	Northern *resident* killer whale (*Orcinus orca*)	Mortality-at-age and average longevity	Olesiuk et al. 2005
		Population growth rate	Olesiuk et al. 1990, Ford et al. 2005
		Population trend	Gaskin 1992, Laake et al. 1998, Baird et al. 2003
Dolphins and Porpoises	Harbour porpoise (*Phocoena phocoena*)	Food consumption	Lockyer et al. 2003, Cosewic 2016
		Distribution, weight estimates	Heise et al. 1997, Klinkenberg 2021
	Pacific white-sided dolphin (*Lagenorhynchus obliquidens*)	Food consumption	Reichsteiner et al. 2013
		Distribution	Morejohn 1979, Jefferson 1988
	Dall’s porpoise (*Phocoenoides dalli*)	Food consumption	Ohizumi et al. 1998
		Population growth rate, weight-at-age, mortality, food consumption	Olesiuk 1993
HS	Harbor seal (*Phoca vitulina*)	Adult weight estimates	Pitcher and Calkins 1979
		Population trend	Jeffries et al. 2003, Brown et al. 2009, DFO 2022
		Population demographics, growth rate, survival-at-age, longevity	Olesiuk 2018
SSL	Steller sea lion (*Eumetopias jubatus*)	Population trend	Olesiuk 2018, DFO 2021
		Weight-at-age	Winship et al. 2001
		Food consumption	Winship et al. 2002
CSL	California sea lion (*Zalophus californianus*)	Population trend	Laake et al. 2018
		Food consumption, adult weight estimates	Weise and Harvey 2018, Kastelein et al. 2000
		Survival-at-age and average longevity	Delong et al. 2017
		Population growth rate	NOAA 2011
Seabirds	Common murre (*Uria aalge*)	Population trend, distribution, average survival rates	Manuwal et al. 2001
		Adult weight estimates	BC Centre for conservation 1996a
		Food consumption	Gabrielsen 1996, Roth et al. 2008
	Rhinoceros auklets (*Cerorhinca monocerata*)	Distribution	Gaston et al. 2009
		Adult weight estimates	BC Centre for conservation 1996b
		Average survival rates	Bertram et al. 2000
		Food consumption	Vermeer and Devito 1986

**Table 2:** Literature sources used to compute Ecopath parameters of the four Pacific salmon species included in the model.

Functional group	Species	Parameter	Literature
Chinook salmon (all groups)	Chinook salmon (*Returning spawners*) (*Oncorhynchus tshawytscha*)	Catch estimates, description of stanzas, mortality	PSC 2021*
		Escapement and Terminal run estimates	PSC 2021*, PMFC 2022
		Average adult weight	Ford and Ellis 2006
		Von Bertalanffy growth model parameters	FishBase, Froese and Pauly 2023
		Hatchery production	RMIS 2022a
Coho salmon	Coho salmon (*Returning spawners*) (*Oncorhynchus kisutch*)	Description of stanzas	Sandercock 1991, Beamish and Sweeting 1999, Beamish et al. 2004
		Von Bertalanffy growth model parameters	FishBase, Froese and Pauly 2023
		Marine survival	Coronado and Hilborn 1998
		Catch estimates, Escapement, Cohort estimates	PSC reports from 1986 to 2010*, PFMC 2022
		Hatchery production	RMIS 2022b
Chum salmon	Chum salmon (leading stanza: *Returning spawners*) (*Oncorhynchus keta*)	Description of stanzas	Fisheries and Oceans Canada 1999, Grant and Pestal 2009
		Distribution, Abundance estimates, average weight	PSC 1987, Grant and Pestal 2009
		Average adult weight	PSF 2023
		Von Bertalanffy growth model parameters	FishBase, Froese and Pauly 2023
		Marine survival	Bradford 1995
		Hatchery production	RMIS 2022
Other salmonids	Pink salmon (*Oncorhynchus gorbuscha*)	Escapement estimates, average adult weight	Ruggerone and Irvine 2018
		Catch estimates	Salmonid Catch Statistics (NPAFC 2023)
		Mortality estimates	Aydin et al.2003, Preikshot 2007
		Consumption estimates	FishBase, Froese and Pauly 2023
	Sockeye salmon (*Oncorhynchus nerka*)	Escapement estimates, average adult weight	Ruggerone and Irvine 2018
		Catch estimates	Salmonid Catch Statistics (NPAFC 2023)
		Mortality estimates	Aydin et al.2003, Preikshot 2007
		Consumption estimates	FishBase, Froese and Pauly 2023

*All PSC estimates for Chinook and coho salmon can be found in individual annual reports available from https://www.psc.org/publications/technical-reports/technical-committee-reports/

**Table 3:** Literature sources used to compute Ecopath parameters for Pacific herring, lingcod, halibut, rockfish, and Pacific hake.

Functional group	Species	Parameter	Literature
Pacific herring	Herring (*Adult*) (*Clupea pallasii*)	Description of stanzas	Taylor 1964
		Spawner abundance	Thompson et al. 2017
		Mortality estimates, Von Bertalanffy growth model parameters	FishBase, Froese and Pauly 2023
Lingcod	Pacific lingcod (*Ophiodon elongatus*)	Biomass estimates, fisheries catch	Fisheries and Oceans 2011
		Mortality estimates, food consumption	FishBase, Froese and Pauly 2000
Halibut	Pacific halibut (*Hippoglossus stenolepis*)	Biomass estimates, fisheries catch	Sullivan et al. 1999
		Mortality estimates, food consumption	FishBase, Froese and Pauly 2023
Rockfish	Copper rockfish (*Sebastes caurinus*)	Biomass and mortality estimates, food consumption	FishBase, Froese and Pauly 2023, Preikshot 2017
	Puget Sound rockfish (*Sebastes emphaeus*)		
	Quillback rockfish (*Sebastes maliger*)		
	Black rockfish (*Sebastes melanops*)		
	China rockfish (*Sebastes nebulosus*)		
	Tiger rockfish (*Sebastes nigrocinctus*)		
	Yelloweye rockfish (*Sebastes ruberrimus*)		
Pacific hake	Pacific hake	Biomass estimates	Dorn et al. 1998
		Mortality estimates, food consumption	FishBase, Froese and Pauly 2023

**Table 4:** List of all parameters entered in Ecopath for the northeast Pacific model for the year 1979. Those values were entered based on estimates found in previous literature and were not generated by the model.

Functional group	Biomass in habitat area (t/km^2^) B	Total mortality (/year) Z	Production / biomass (/year) P/B	Consumption / biomass (/year) Q/B	Catch (t/km^2^) Y	Other production
TKW	0.000046 **		0.044	11.08		
SRKW	0.00084 *		0.05208	10.17		**0.005**
NRKW	0.0017 *		0.0416	10.17		**0.023**
Porpoises/dolphins	0.0125 ***		0.3	38.37		
Harbor seals	0.00863 **		0.212	15.5		0.012
Steller sea lions	0.0053 **		0.162	24.45		0.048
California sea lions	0.0229 **		0.144	16.89		0.054
Seabirds	0.0058 ***		0.1375	93.7		
FRGSPS SP						
FRGSPS SP *River*	*0*.*000377762*	0.5		8.98533		
FRGSPS SP *Smolts*	*0*.*000449512*	3.5		5.771215		
FRGSPS SP *Juveniles*	*0*.*000933173*	0.8		4.058203		
FRGSPS SP *Marine*	*0*.*002487325*	0.44		2.834945	**3.11E-05**	
FRGSPS SP *Returning spawners*	0.006 **	0.72		2	**0.003074**	
FRGSPS SP *Escapees*	*0*.*05373438*	0.157		1.050405	**0.002929**	
FRGSPS SU						
FRGSPS SU *River*	*1*.*17E-05*	1		39.86551		
FRGSPS SU *Smolts*	*0*.*00090348*	3.5		10.44263		
FRGSPS SU *Juveniles*	*0*.*003396815*	0.510826		4.300026		
FRGSPS SU *Marine*	*0*.*007634544*	0.55		2.813696	**0.000938**	
FRGSPS SU *Returning spawners*	0.015 **	0.89		2	**0.010379**	
FRGSPS SU *Escapees*	*0*.*1190069*	0.15		1.031226	**0.005724**	
FRGSPS FA						
FRGSPS FA *River*	*3*.*28E-05*	1		39.59757		
FRGSPS FA *Smolts*	*0*.*003113446*	3		10.03799		
FRGSPS FA *Juveniles*	*0*.*01393653*	0.5		4.268976		
FRGSPS FA *Marine*	*0*.*03764028*	0.2		2.767977	**0.000903**	
FRGSPS FA *Returning spawners*	0.08 **	1		2	**0.064264**	
FRGSPS FA *Escapees*	*0*.*1849568*	0.31		1.172155	**0.03246**	
WCVI FA						
WCVI FA *River*	*1*.*35E-05*	1		39.59756		
WCVI FA *Smolts*	*0*.*000854519*	**4**		10.73029		
WCVI FA *Juveniles*	*0*.*00269063*	**0.510826**		4.271125		
WCVI FA *Marine*	*0*.*005812909*	**0.63**		2.801022	**0.001227**	
WCVI FA *Returning spawners*	0.01 **	**1**		2	**0.005365**	
WCVI FA *Escapees*	*0*.*0451843*	**0.2**		1.078224	**0.004189**	
CRWORC SP	* *	** **			** **	
CRWORC SP River	*0*.*000372801*	1		9.076533		
CRWORC SP Smolts	*0*.*00046814*	**2**		5.612062		
CRWORC SP Juveniles	*0*.*00146552*	**0.510826**		3.966077		
CRWORC SP Marine	*0*.*004267717*	**0.459**		2.787735	**0.000299**	
CRWORC SP Returning spawners	0.008 **	**1**		2	**0.004551**	
CRWORC SP Escapees	*0*.*05267467*	**0.156**		1.031227	**0.002829**	
CRWORC SU	* *	** **			** **	
CRWORC SU River	*0*.*002606931*	**1**		9.132318		
CRWORC SU Smolts	*0*.*003273621*	**2**		5.646552		
CRWORC SU Juveniles	*0*.*0102851*	**0.5**		3.989615		
CRWORC SU Marine	*0*.*03097257*	**0.4**		2.800298	**0.004584**	
CRWORC SU Returning spawners	*0*.065 **	**0.9**		2	**0.051773**	
CRWORC SU Escapees	*0*.*6308022*	**0.13**		1.006305	**0.018924**	
CRWORC FA	* *	** **			** **	
CRWORC FA River	*2*.*88E-05*	1		40.11358		
CRWORC FA Smolts	*0*.*003395708*	**2.5**		9.854838		
CRWORC FA Juveniles	*0*.*01772177*	**0.510826**		4.326784		
CRWORC FA Marine	*0*.*03996976*	**0.543**		2.830654	**0.005156**	
CRWORC FA Returning spawners	0.086 **	**0.79**		2	**0.0516**	
CRWORC FA Escapees	*0*.*09529229*	**0.6**		1.339623	**0.034019**	
Coho salmon	* *	** **			** **	
Coho River	*0*.*007927025*	1		25.67039		
Coho Smolts	*0*.*01462168*	2		15.10733		
Coho Marine	*0*.*08549532*	**0.4**		8.861984	**0.0179**	
Coho Returning spawners	0.079 **	**0.8**		6.489996	**0.0166**	
Coho Escapees	*0*.*1448364*	**0.8**		4.902339	** **	
Chum salmon	* *	** **			** **	
Chum River	*0*.*000256017*	1		22.30038		
Chum Smolts	*0*.*04662696*	2.5		4.732551		
Chum Marine	*0*.*1130326*	**1**		2.071343		
Chum Returning spawners	0.07 **	**1.2**		1.4	0.012	
Chum Escapees	*0*.*05169914*	**0.4**		1.023608	** **	
Other salmonids	1.57	** **	1.34	3.8	0.366	
Herring	* *	** **			** **	
Herring Juveniles	*0*.*6268337*	**0.5**		8.277314		
Herring Adults	2.7	**1**		4	0.691	
Halibut	0.11		0.2065	1.85	0.0139	
Hake	4.02		**0.3**	1.85	0.329	
Rockfish	1		0.365	3.11		
Lingcod	0.05		0.54	1.7	0.0121	
Pacific sand lance	**2**		0.77	7.3		
Other forage fish	10 ****		0.77	38.4		
Other fish	40 ****		0.5	2.5		
Invertebrates	**50**		3.05	11.1		
Zooplankton	50		17	50		
Phytoplankton	31		130			
Detritus	10					

Parameters written in bold have been modified through the mass-balancing of the Ecopath model, and parameters written in italic are directly derived from Ecopath. All acronyms found in this table are detailed in the Section 1 of S1 Appendix.

Asterisks correspond to the pedigree applied to the different estimates (sampling locally and high precision (*), sampling locally and low precision (**), approximate or indirect method (***), and guesstimates (****)).

#### Mass-balance

Mass balancing was the next major step to ensure that ecological and thermodynamics rules were followed [74]. Following the equation that describe the production term (Eq 1), parameters were adjusted when the model-derived ecotrophic efficiency (EE) estimates (i.e. proportion of the production used in the model) were too high (>1) [74, 78]. The EE can also be written as EE = 1−other mortalities. In the case of an EE higher than 1, the ‘other mortalities’ estimate would thus be negative, and several scenarios were investigated, including underestimated biomass or production/biomass ratio, or overestimated predation mortality.

The initial biomass estimates were increased for all Chinook salmon *Returning spawners* stanzas, which seemed reasonable given that the salmon abundance estimates provided by the Pacific Salmon Commission do not include all salmon stocks present in the model area at a given time [80]. The catch was adjusted for all groups in order to maintain a similar initial fishing mortality F, based on the fact that F = catch/biomass [81].

Following a similar method, the initial biomass of the *Adult* herring stanza was increased. The biomass estimates initially entered in Ecopath solely involved the spawning biomass and are based on fishery landings of mature individuals (i.e. Age 4+ fish) provided by different governmental organizations [82]. Yet, virtual population analyses and age-structured population dynamics model fitting (i.e. age structure reconstruction) of herring populations suggest that the immature/mature biomass ratio has typically been around 1.33, suggesting that the initial biomass could be multiplied by at least 2.3. Finally, the diet proportions of several functional groups (i.e. pinniped species, salmon species) were adjusted in order to balance the model. All modifications were minor, and deemed reasonable given that most diet studies are carried out in small geographic areas during limited periods of time, hence potentially not accurately representing the annual diet of a species throughout its range.

### Ecosim

Ecosim analysis provide dynamic simulation expressing biomass dynamics through a system of differential equations derived from the Ecopath master equations [77, 83]. In this simulation, temporal variations in biomasses (*dB*_*i*_) of a functional group *i* are estimated by multiplying its consumption (*Q*_*ji*_) with the food conversion efficiency *g*_*i*_, adding immigration (*I*_*i*_) and subtracting mortality terms, i.e. consumption *Q_ij_* of all predators *j*, and the product of the group biomass *B*_*i*_ and the summed non-predation mortality rate *M*0_*i*_, emigration rate(*e_i_*), fishing mortality rate (Fi) and immigration rate (*I_i_*), expressed as follows:

$$\frac{{dB}_{i}}{dT}=g_{i}\sum_{j}Q_{ji}-\sum_{j}Q_{ij}+I_{i}-({M0}_{i}+F_{i}+e_{i})*B_{i}$$ (Eq 3)

The consumption rates are calculated based on the ‘foraging arena’ concept, according to which prey move from ‘refuges’ to ‘foraging arenas’ where they are vulnerable to predators [81, 84, 85]. This concept is illustrated in Ecosim using ‘vulnerability multipliers’, which allow for prediction of the magnitude at which an increase in predator’s abundance will influence the predation mortality of a given prey [77]. The vulnerability multipliers express how much the predation mortality of a prey may increase if the predators were to increase to the carrying capacity. A vulnerability of 1 thus imply that the predator is at carrying capacity and hence cannot increase the predation mortality it is causing on its prey–at carrying capacity the predator is limited by available prey. Conversely, a high vulnerability multiplier imply that a predator is far from its carrying capacity and may be able to increase prey predation mortality as it grows towards its carrying capacity. Ecosim simulations permit to fit predicted temporal changes in biomass and mortality to time-series of reference data. Except if stated otherwise, all biomass time-series were entered as relative to the base Ecopath biomass estimates of the different functional groups in 1979 [77]. Detailed descriptions of how each Ecosim time-series was calculated and used can be found in the Section 3 of S1 Appendix. For multi-stanza populations, the differential equations are replaced by a discrete-time (monthly) age structured population model that uses rates calculated from the differential equation model rates for fish of all ages that are in each stanza each month [86].Finally, time-series of fishing mortality were entered for each functional group of the model between 1979 and 2020.

After the time-series were loaded and applied, we generated a statistical measure of goodness of fit (i.e. a weighted sum of sum of squared deviations of log biomasses from log predicted biomasses, often referred to as SS residuals) for each Ecosim run [77]. The SS residuals are sensitive to the vulnerability multipliers for each functional group and their prey, as those trophic flow between predators and their prey [77, 81]. Those vulnerability estimates were generated by Ecosim to improve the ‘rightness’ of fit (i.e. lower SS) based upon the influence of other driving factors, including primary productivity and fishing activity. In addition, we ran a search for primary production anomalies between 1979 and 2020, and applied the resultant as a forcing function for the ‘producers’ functional group (i.e. phytoplankton). We then compared this forcing function to three different environmental indices: NPGO, DPO, and ENSO. Anomaly indices for the NPGO were downloaded directly from the Georgia Institute of Technology website (http://www.o3d.org/npgo/) [87]. Further, both DPO (https://www.ncei.noaa.gov/access/monitoring/pdo/, [88]) and ENSO (https://www.ncei.noaa.gov/access/monitoring/enso/) anomaly indices were extracted from the National Centers for Environmental Information of the National Oceanic and Atmospheric Administration. For each climatic index, the primary productivity anomaly function extracted from Ecosim was rescaled for comparison. The NPGO, PDO, and ENSO environmental indexes were not initially entered in the model, as it was deemed important to understand whether the model itself would accurately predict changes in primary productivity between 1979 and 2020.

## Results

The main objective of this model was to examine whether competition for food between marine mammals could be a limiting factor for the SRKW population, and to understand how the predation mortality associated with growing populations of marine mammals has changed over the northeast Pacific coast between 1979 and 2020. *Resident* killer whales are primarily feeding on mature fish on their way to spawning grounds, while harbor seals primarily target young smolts entering the marine environment. Steller and California sea lions target Pacific salmonids through different life stages but are mostly found foraging in coastal waters. To illustrate changes in predation mortality, we thus chose to present the results of six marine mammal groups (i.e. TKW, SRKW, NRKW, HS, SSL, CSL) and 16 salmon groups (i.e. *Smolts* and *Returning Spawners* stanzas for all Chinook and coho salmon). EwE simulation output with changes in biomass of all other functional groups can be found in the Figs B1-B7 of S2 Appendix. The biomass trajectories predicted by the EwE simulations and presented here are those for which we obtained the ‘best fit’ (i.e. the lowest sums of squared differences between the predicted and observed biomass estimates based upon optimal vulnerability parameters). The biomass predictions are presented for marine mammals and *Returning spawners* stanzas only. Detailed information about the different vulnerability settings used in the EwE simulations can be found in the Tables A5-A7 of S1 Appendix.

### Population biomass

The overall sums of squared differences before model fitting was 86.8. The fit was improved to 55.60 when using search-derived vulnerability multipliers (Section 4, Tables A5-A7 of S1 Appendix). Finally, the primary productivity anomaly estimated by Ecosim was applied to all time-series and further improved the overall sums of squared differences to 49.7. After applying the primary productivity anomaly, the fit was improved for all Chinook salmon *Returning spawners* stanzas.

**Fig 2:**
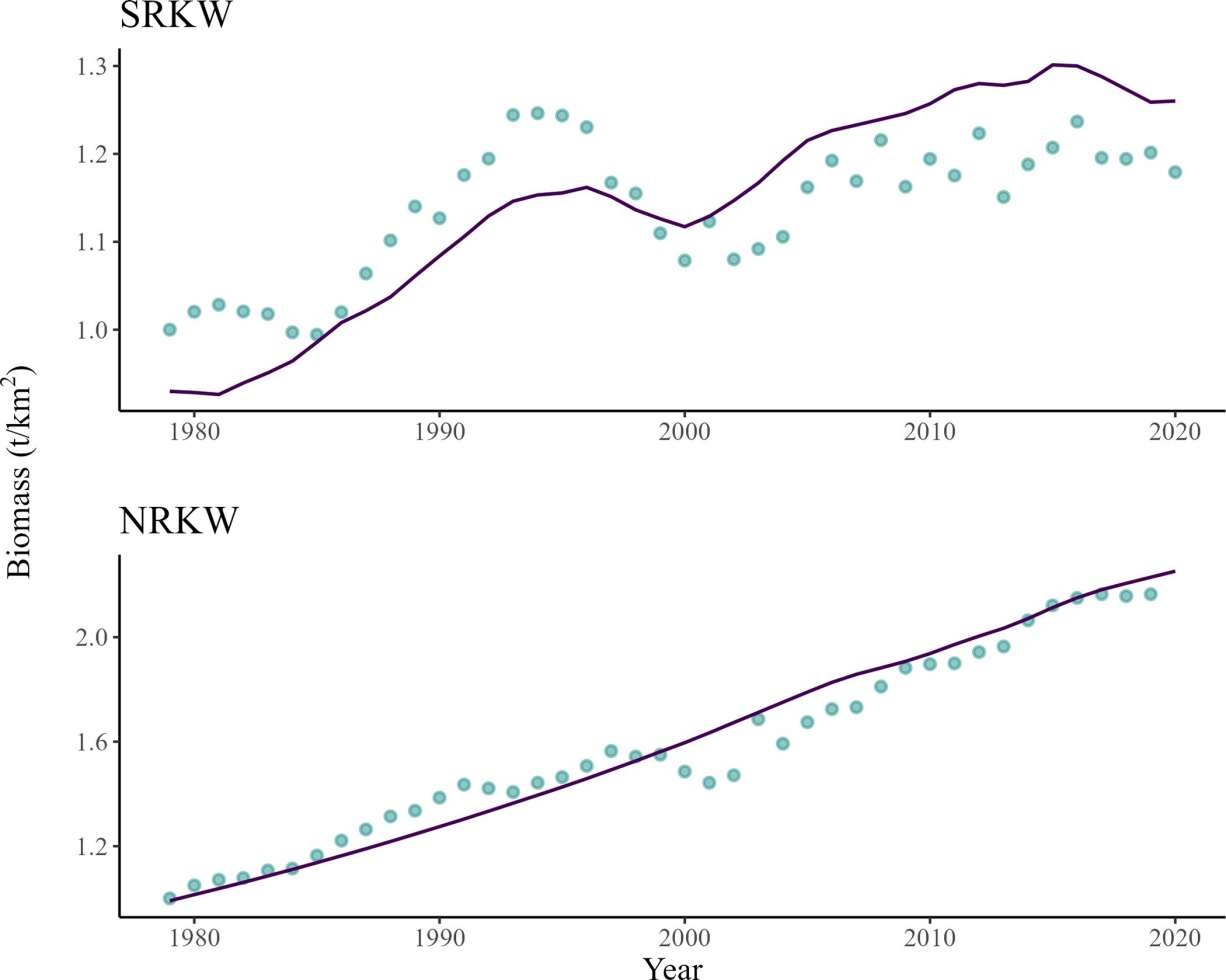
Comparison of changes in SRKW (top) and NRKW (bottom) relative predicted biomass (line) versus biomass estimates from population censuses (points) between 1979 and 2020.

The model predicted that the population trend of SRKW has been fluctuating slightly since 1979 (Fig 2). This result is in line with the population censuses reported by the Center for Whale Research (2022), but with less amplitude [89]. However, the bioaccumulation for this population had to be lowered (to 0.5%) in order to achieve this fit. This modification is not surprising, as the growth rate of the population that we initially used (i.e. 1.3%) was an annual average between 1974 and 1987 [90]. Besides, the SRKW population has undergone periods of negative population growth, including in the early 1980s [91] (Fig 2). The model also predicted that the NRKW population would have increased by 2.1-fold between 1979 and 2020, which is in line with previous estimates [1, 92] (Fig 2).

**Fig 3:**
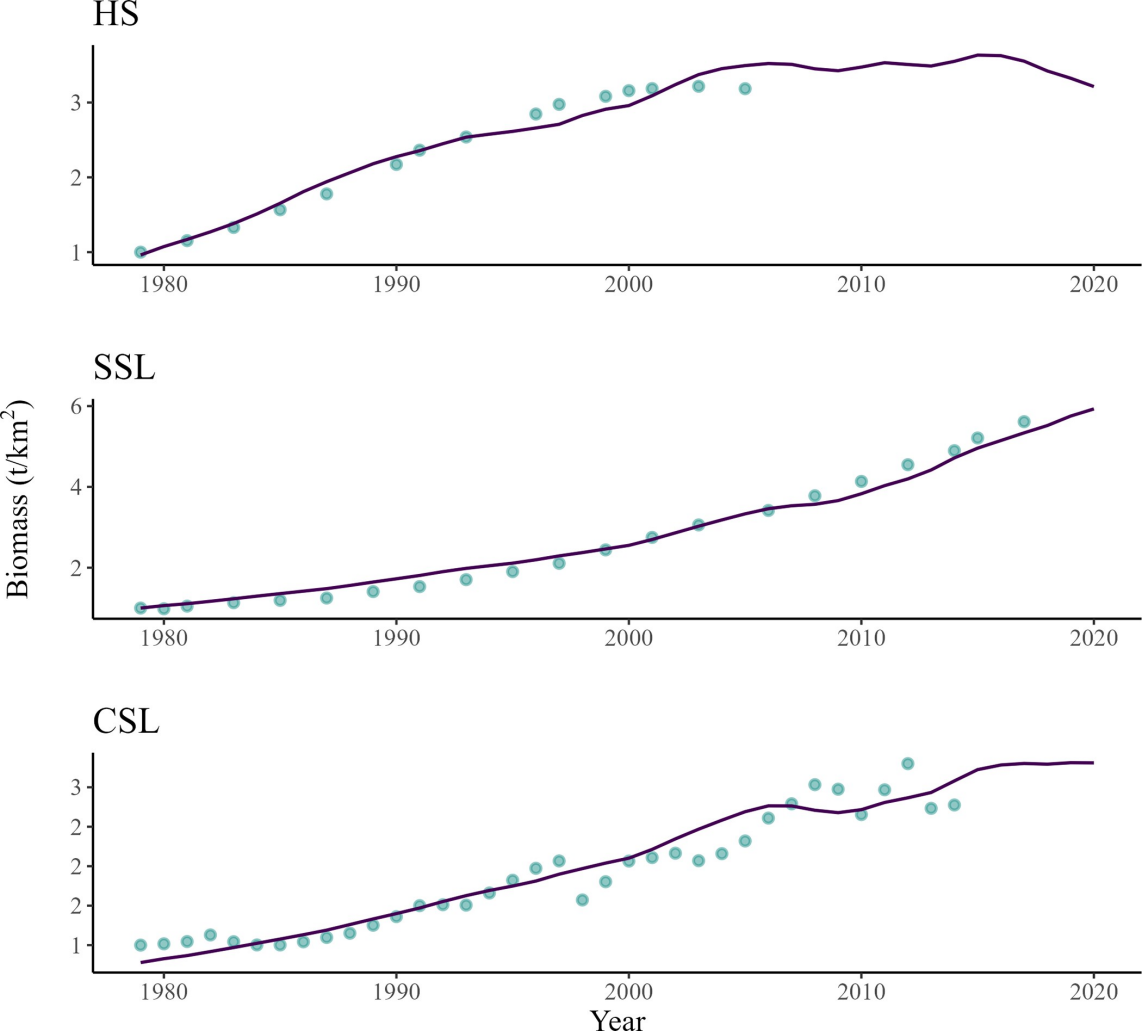
Comparison of changes in HS (top), SSL (middle), and CSL (bottom) relative predicted biomass (line) versus biomass estimates from population censuses (points) between 1979 and 2020.

The model predicted that the three populations of pinnipeds consistently increased from 1979 on (Fig 3). The EwE simulation predicted that the harbor seal population rose by about 3.2 times between 1979 and the early 2000s, before plateauing in the early 2000’s. The population of Steller and California sea lions increased by about six and three times since 1979, respectively. The growth of the CSL population was predicted to slow down around 2015 (Fig 3).

**Fig 4:**
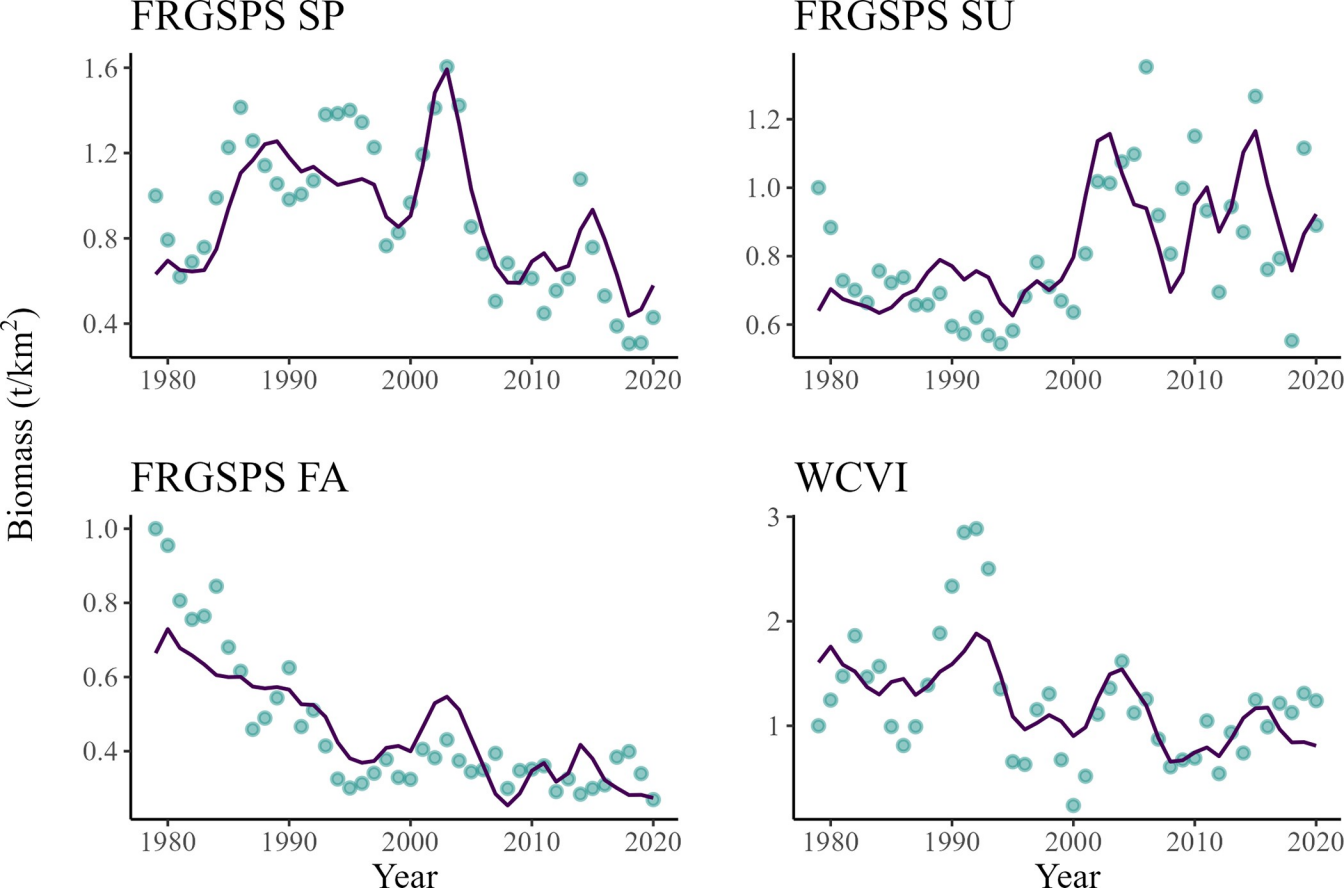
Comparison of changes in relative predicted biomass (line) of spring (top left), summer (top right), and fall (bottom left) *Returning Spawners* Chinook salmon originating from the Salish Sea (Fraser River/ Georgia Strait/Puget Sound) and the west coast of Vancouver Island (bottom right) versus biomass estimates from CWT recoveries (points) between 1979 and 2020.

The predicted variations in population trends of *Returning spawners* of Chinook salmon were similar to the trends extracted from CWT recoveries for all three runs originating from the Salish Sea (Fraser River/Georgia Strait/Puget Sound) (Fig 4). A peak in abundance was predicted for all three groups in the early 2000s (Fig 4). Overall, the abundance of FRGSPS spring Chinook salmon group was predicted to only slightly decrease (~ -8%) between 1979 and 2020. The FRGSPS summer Chinook salmon group increased by about 43% between 1979 and 2020, whereas the FRGSPS fall Chinook group was predicted to decline by about 90% throughout the time series (Fig 4). Finally, the model predicted that the overall abundance of the *Returning spawners* Chinook salmon originating from the west coast of Vancouver Island decreased by about 50% between 1979 and 2020 (Fig 4).

**Fig 5:**
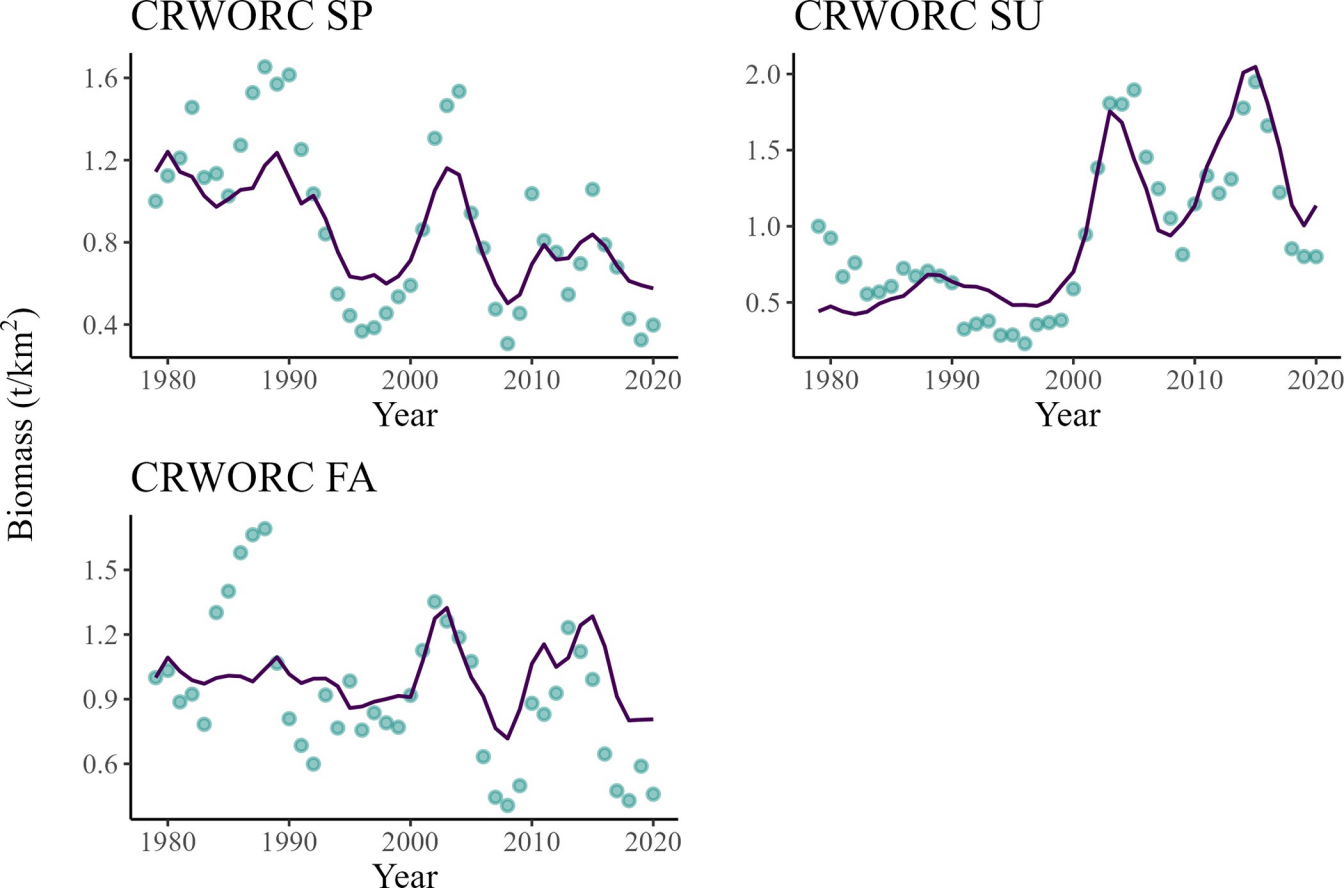
Comparison of changes in relative predicted biomass (line) of *Returning spawners* Chinook salmon originating from the Columbia River/Oregon/Washington/California coast (CRWORC) in the spring (top), summer (middle), and fall (bottom) versus biomass estimates from CWT recoveries (points) between 1979 and 2020.

The predicted variations in population trends of *Returning spawners* Chinook salmon were similar to the trends extracted from CWT recoveries for all three runs originating from the Columbia River/Washington/Oregon/California coasts (Fig 5). Similarly to the FRGSPS groups, a peak in abundance was predicted for all three groups between 2000 and 2007. A second peak was predicted for the *Returning spawners* summer run between 2010 and 2018. The model predicted that this functional group would have increased by 61% during that time. Finally, an overall decline in abundance was predicted between 1979 and 2020 for the spring and fall run, which decreased by 50% and 20%, respectively (Fig 5).

**Fig 6:**
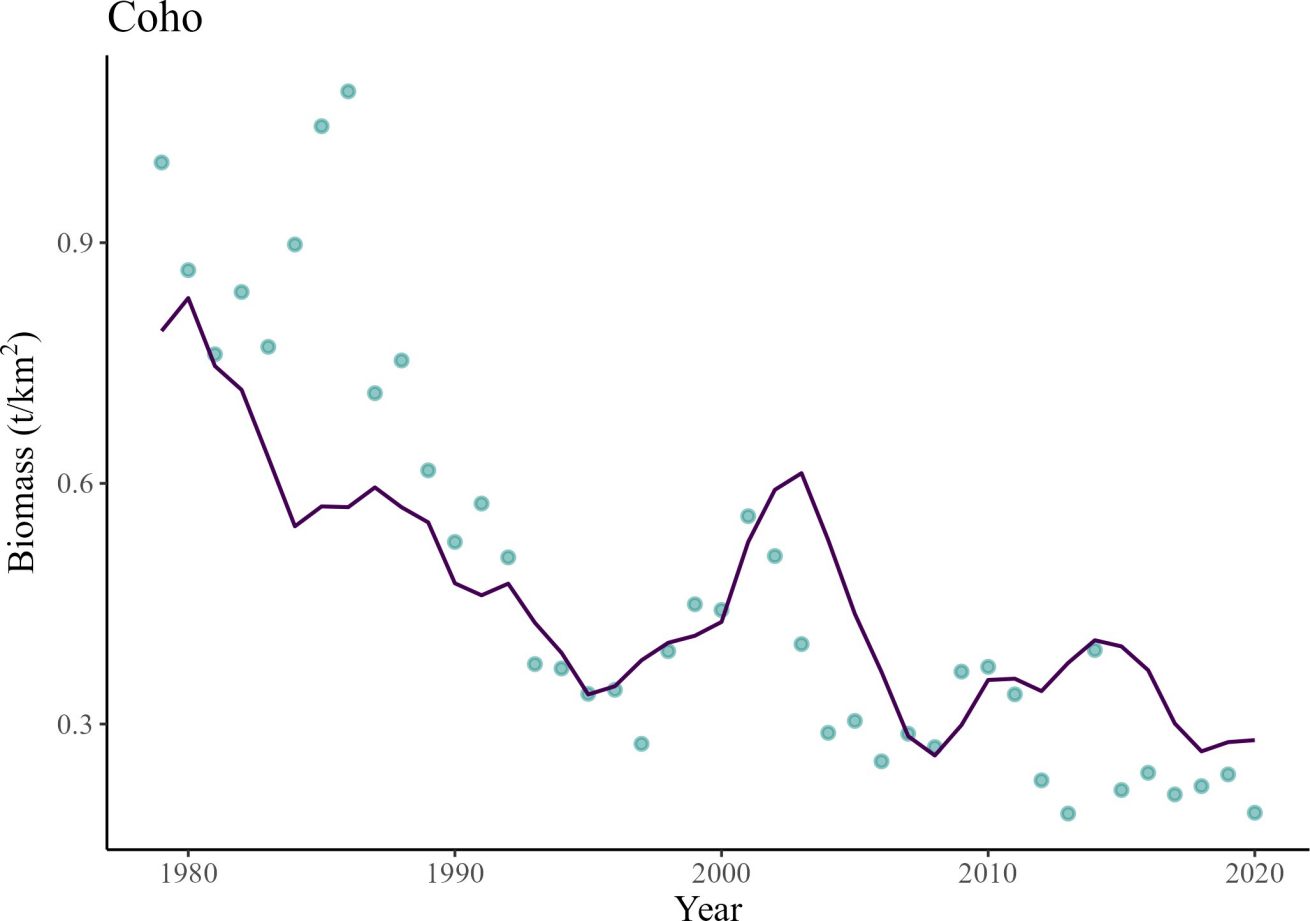
Comparison of changes in coho salmon *Returning spawners* relative predicted biomass (line) versus biomass estimates from catch and escapement (points) data between 1979 and 2020.

The model predicted that the coho salmon population would drastically decrease between 1979 and 2020 (Fig 6), which, however, was a slightly less severe decline than in previous population estimates [93, 94]. Overall, the model predicted a 66% biomass decrease between 1979 and 2020 (Fig 6).

### Mortality

**Fig 7:**
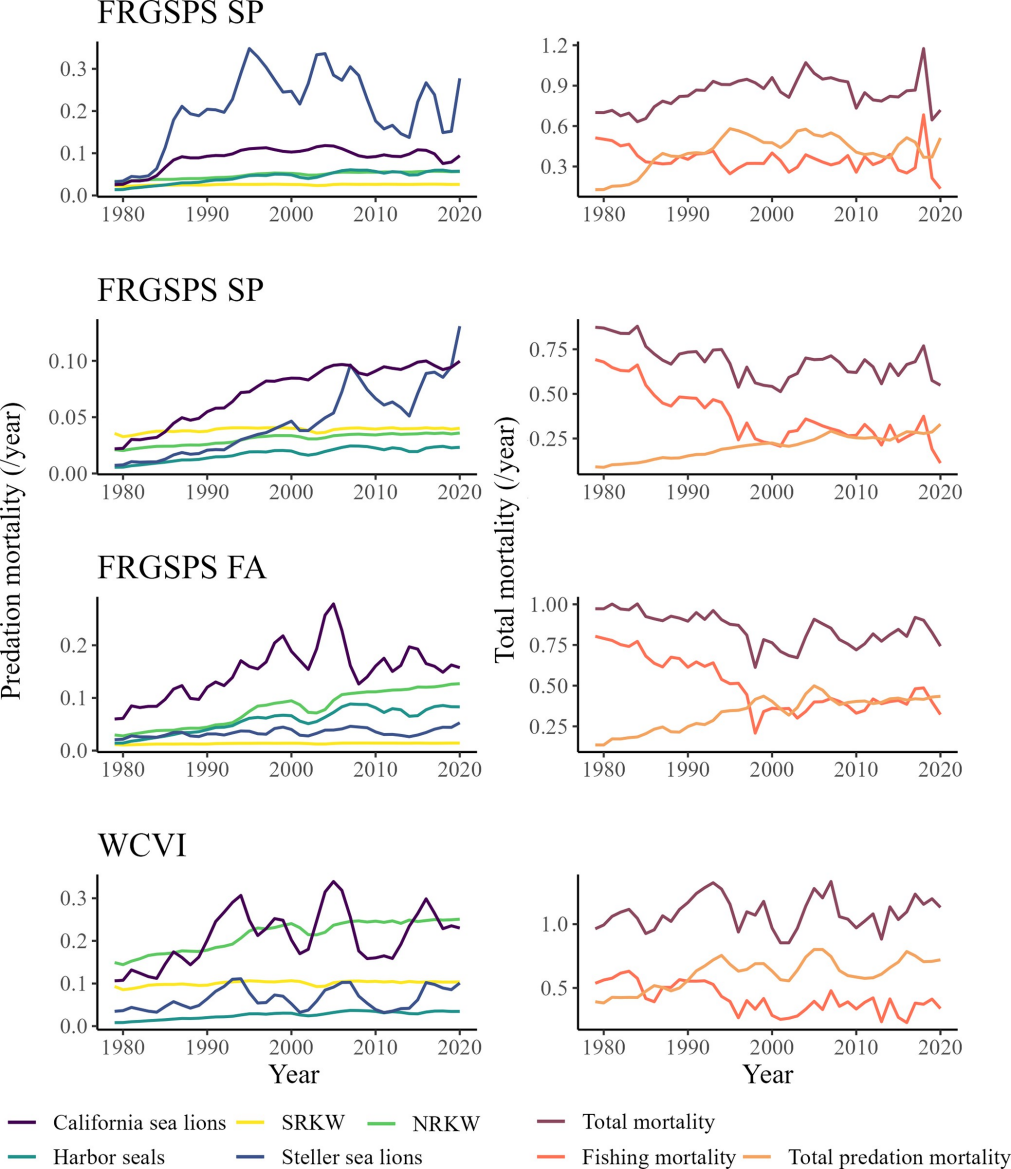
Relative marine mammal predation mortality (left column) and total mortality (including fishing and predation mortality) (right column) on *Returning spawners* Chinook salmon originating from the Salish Sea (i.e. FRGSPS) and the west coast of Vancouver Island from 1979 to 2020. The graphs are presented for FRGSPS SP (first row), FRGSPS SU (second row), FRGSPS FA (third row), and the WCVI (fourth row).

The model predicted that total mortality of all Chinook salmon *Returning spawners* groups originating from the Salish Sea (i.e. FRGSPS) and the west coast of Vancouver Island has been relatively constant between 1979 and 2020 (Fig 7). This is the case despite a consistent decrease in fishing mortality during this period, including for FRGSPS SP (-73.6%), FRGSPS SU (-83.8%), FRGSPS FA (-59.9%), and WCVI (-36.9%) (Fig 7). In contrast, the predation mortality associated with marine mammals has increased for all groups between 1979 to 2020 (Fig 7). The model predicted that the predation mortality on FRGSPS SP *Returning spawners* was multiplied by 3.9 between 1979 and 2020, and by 3.6, 3.1, and 1.8 for FRGSPS SU, FRGSPS FA, and WCVI, respectively (Fig 7). This increase in predation mortality is driven by different marine mammals depending on the Chinook salmon functional group (Fig 7). In 2020, California sea lion accounted for almost 40% of the predation mortality predicted for FRGSPS SU while Steller sea lion accounted for 54.2% of the predation mortality predicted for FRGSPS SP Chinook salmon *Returning spawners*. Finally, NRKW and California sea lion together accounted for 66.7% of the predation mortality predicted for WCVI *Returning spawners* in 2020.

**Fig 8:**
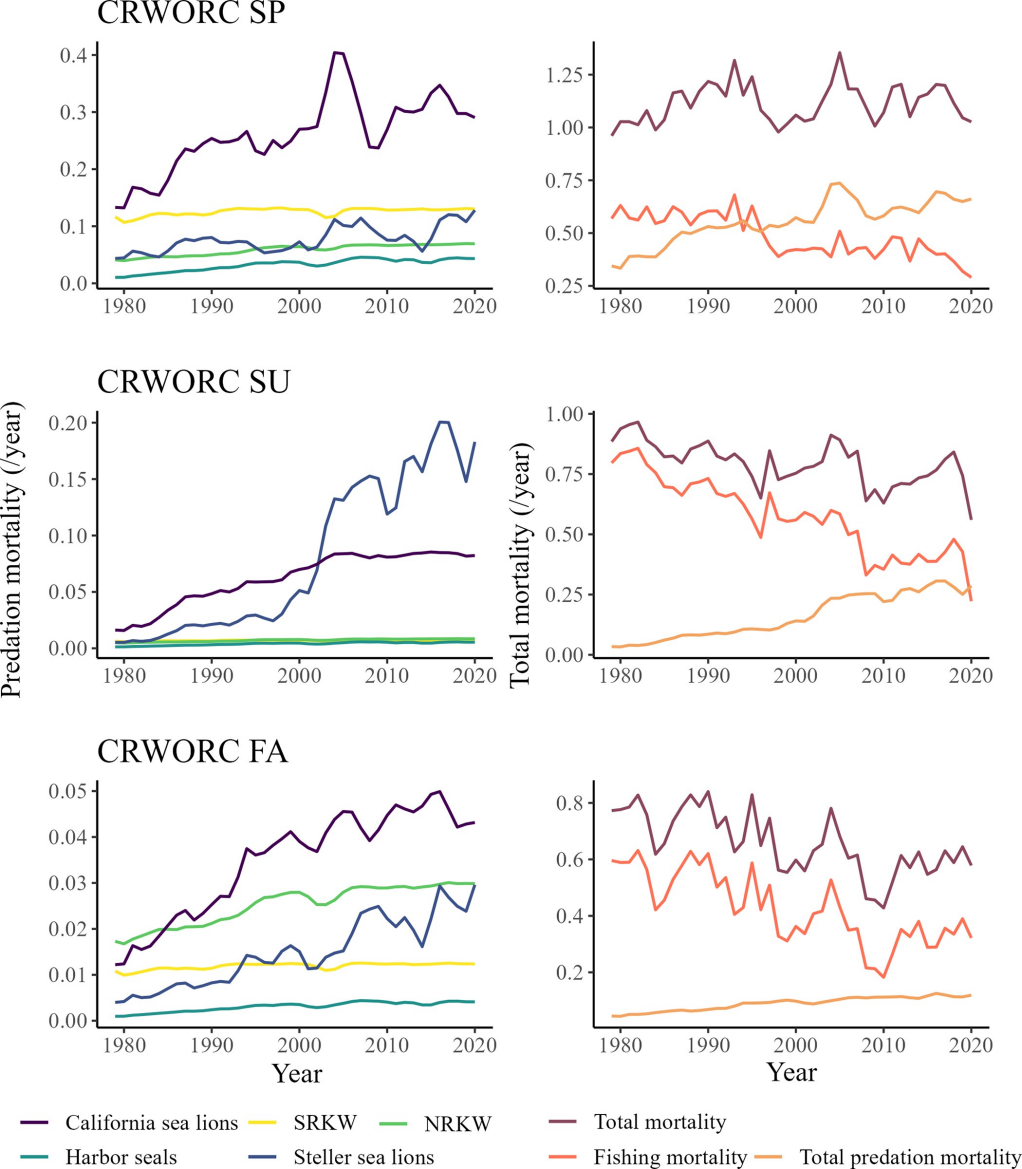
Relative marine mammal predation mortality (left column) and total mortality (including fishing and predation mortality) (right column) on *Returning spawners* Chinook salmon originating from the Columbia River and the Washington, Oregon, and California coast (i.e. CRWORC) from 1979 to 2020. The graphs are presented for CRWORC SP (top row), CRWORC SU (middle row), and CRWORC FA (bottom row).

The model predicted similar trends of predation and total mortality for Chinook salmon *Returning spawners* groups originating from the southern part of the model (i.e. CRWORC). The total mortality slightly decreased for CRWORC SU (-37%) and CRWORC FA (-26%) between 1979 and 2020, while their respective fishing mortality during that time was reduced by 72% and 45% (Fig 8). During this period, the predation mortality on CRWORC SU and CRWORC FA increased by factors of 8.5 and 2.6, respectively. The total predicted mortality for CRWORC SP Chinook salmon *Returning spawners* slightly increased (+18.6%) from 1979 to 2020 (Fig 8). During this time, the fishing mortality decreased by 49% while the predation mortality doubled (Fig 8). For this group, the combined predation of Steller and California sea lions accounted for 63.3% of the total predation mortality in 2020. During this year, Steller sea lion accounted for about 64% of the predation mortality predicted for CRWORC SU Chinook salmon *Returning spawners*. Finally, NRKW accounted for 25% of the predation mortality predicted for CRWORC FA (Fig 8).

The predation mortality of FRGSP SP, FRGSPS SU, CRWORC SP, CRWORC SU, and CRWORC FA *Smolts* was not predicted to vary significantly throughout the time series, as very little predation was applied on those groups.

**Fig 9:**
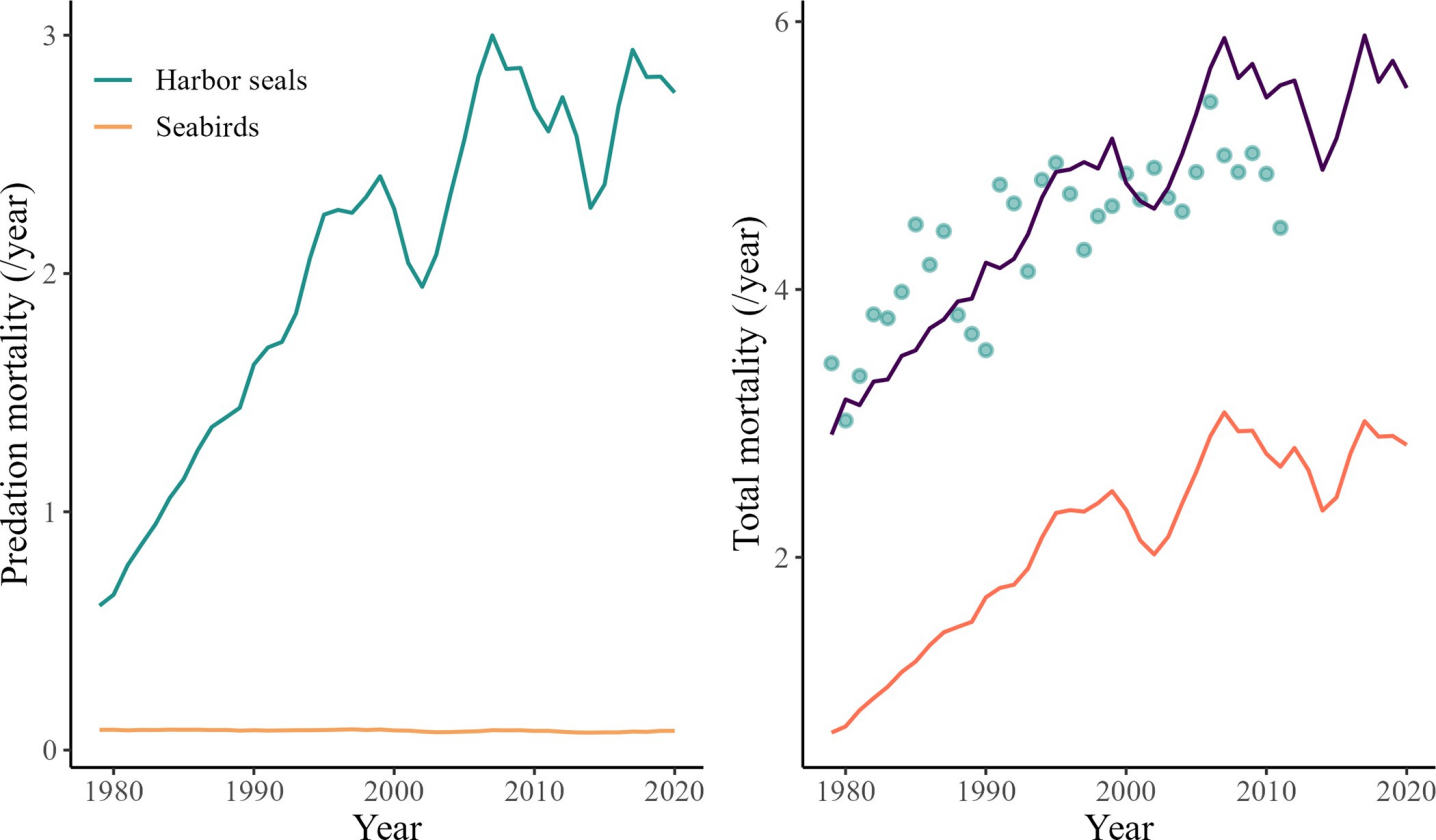
The first graph (left) shows the relative predation mortality of harbor seals and sea birds on FRGSPS FA Chinook salmon *Smolts* predicted by the model between 1979 and 2020. The second graph (right) is a comparison of the predicted total mortality Z (thick purple line) versus total mortality estimates from CWT recoveries (points) from 1979 to 2020 (top).

The predation mortality associated with harbor seals on FRGSPS FA *Smolts* was predicted to increase from 0.6 to 2.8 between 1979 and 2020 (Fig 9). This increase follows the same variation patterns as predicted by the model for total mortality. The total mortality was predicted to have doubled between 1979 and 2020, which is in line with observations and data from CWT recoveries [80] (Fig 9).

**Fig 10:**
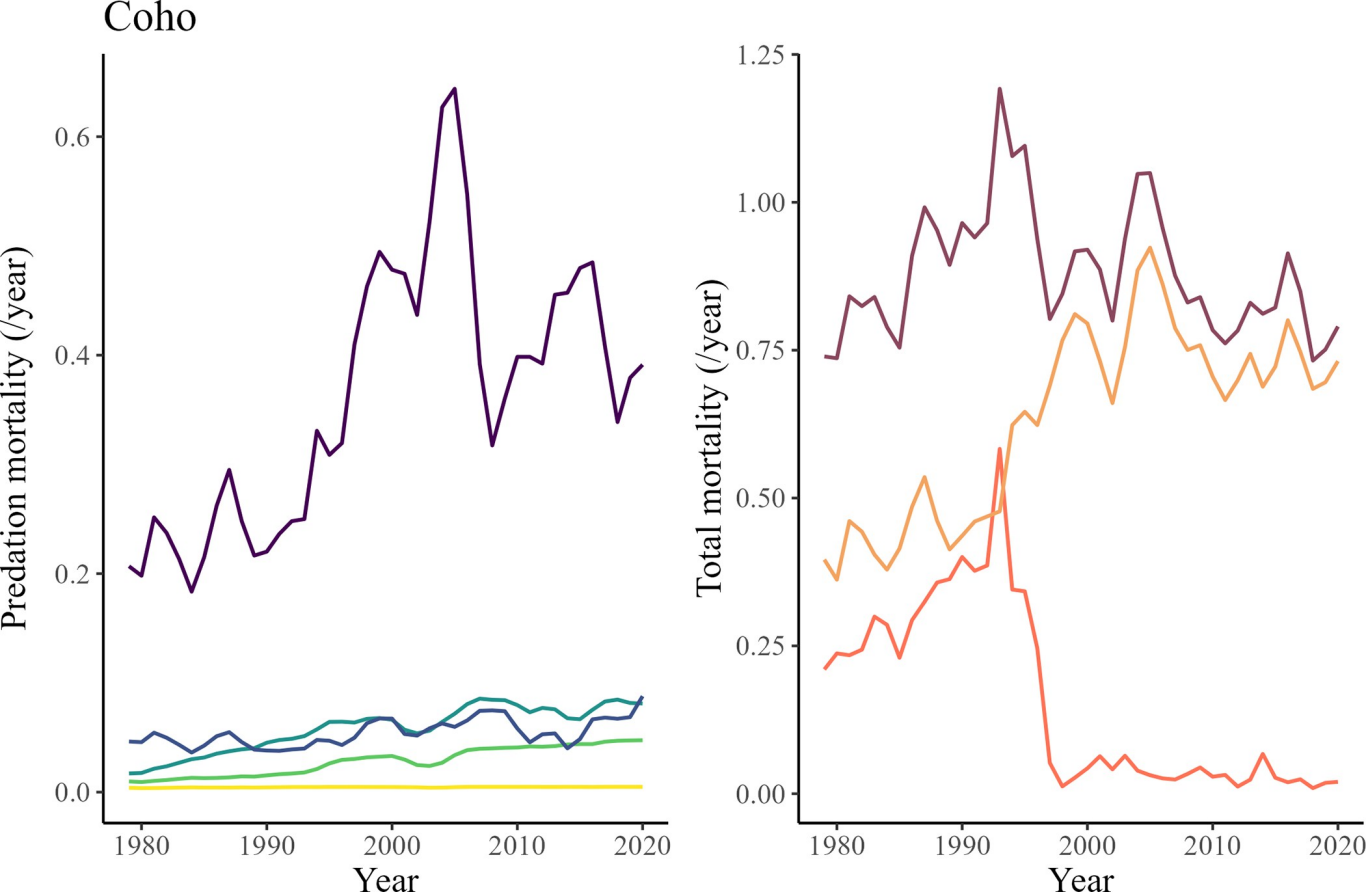
The first graph (left) shows the relative predation mortality of marine mammals on coho salmon *Returning spawners* predicted by the model between 1979 and 2020. The second graph (right) shows the predicted total mortality Z (thick purple line, includes fishing and predation mortality) for this group from 1979 to 2020.

Despite a drastic reduction in fishing mortality (- 90%) on *Returning spawners* coho salmon between 1979 and 2020, the model predicted that the total mortality of this group would have stayed relatively stable during that time. This is because the predation mortality on this group was predicted to almost double between 1979 and 2020 (Fig 10). California sea lions accounted for more than 50% of this predation mortality throughout the 42 years of the model.

**Fig 11:**
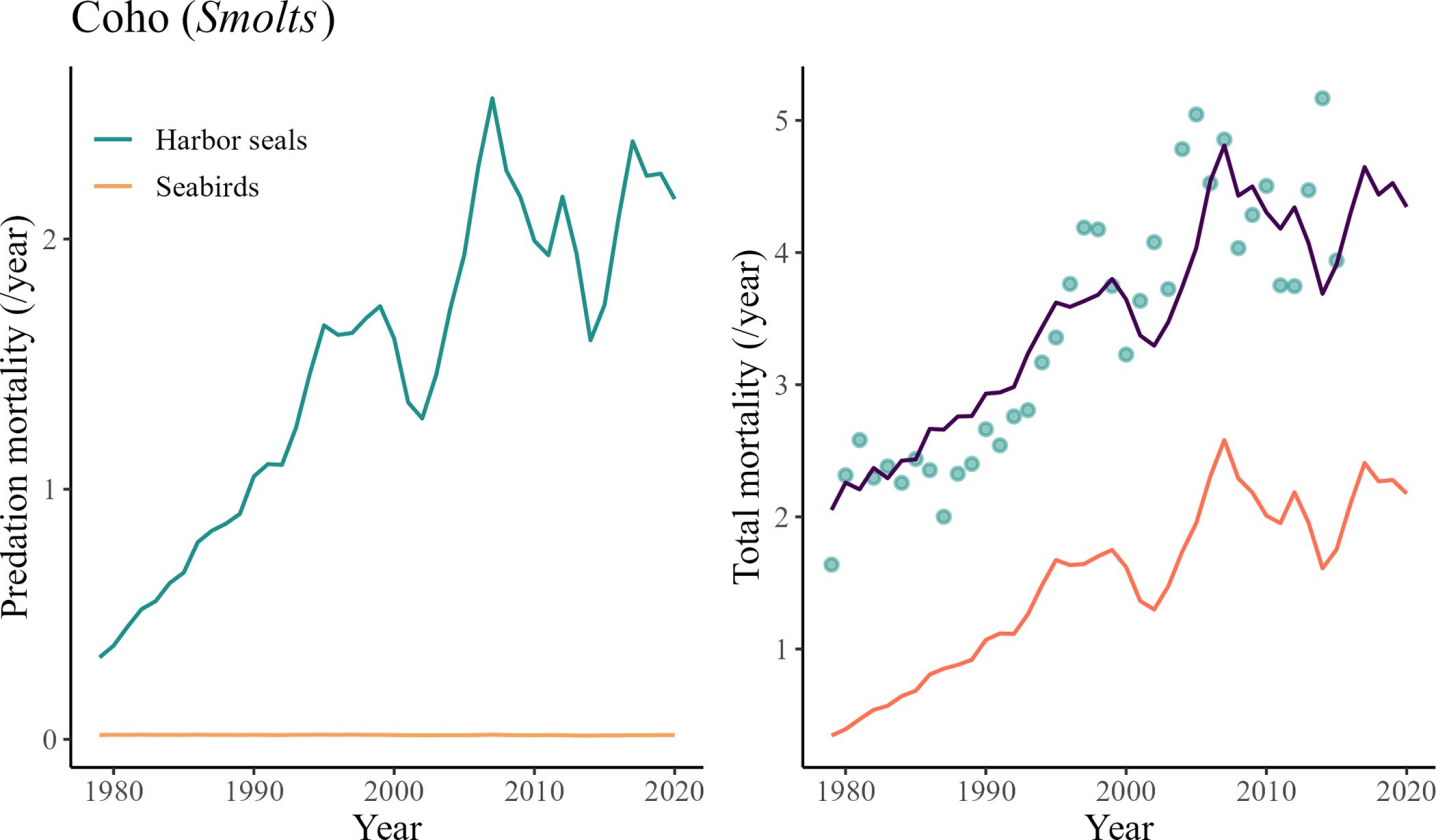
The first graph (left) shows the relative predation mortality of harbor seals and sea birds on coho salmon *Smolts* predicted by the model between 1979 and 2020. The second graph (right) is a comparison of the predicted total mortality Z (thick purple line) versus total mortality estimates from CWT recoveries (points) from 1979 to 2020.

The predation mortality due to harbor seals on coho salmon *Smolts* was predicted to increase from 0.32 to 2.2 between 1979 and 2020 (Fig 11). This increase follows the same variation pattern as predicted by the model for total mortality (Fig 11). The total mortality was predicted to have doubled between 1979 and 2020, which is in line with observations and data extracted from CWT recoveries (Fig 11).

**Fig 12:**
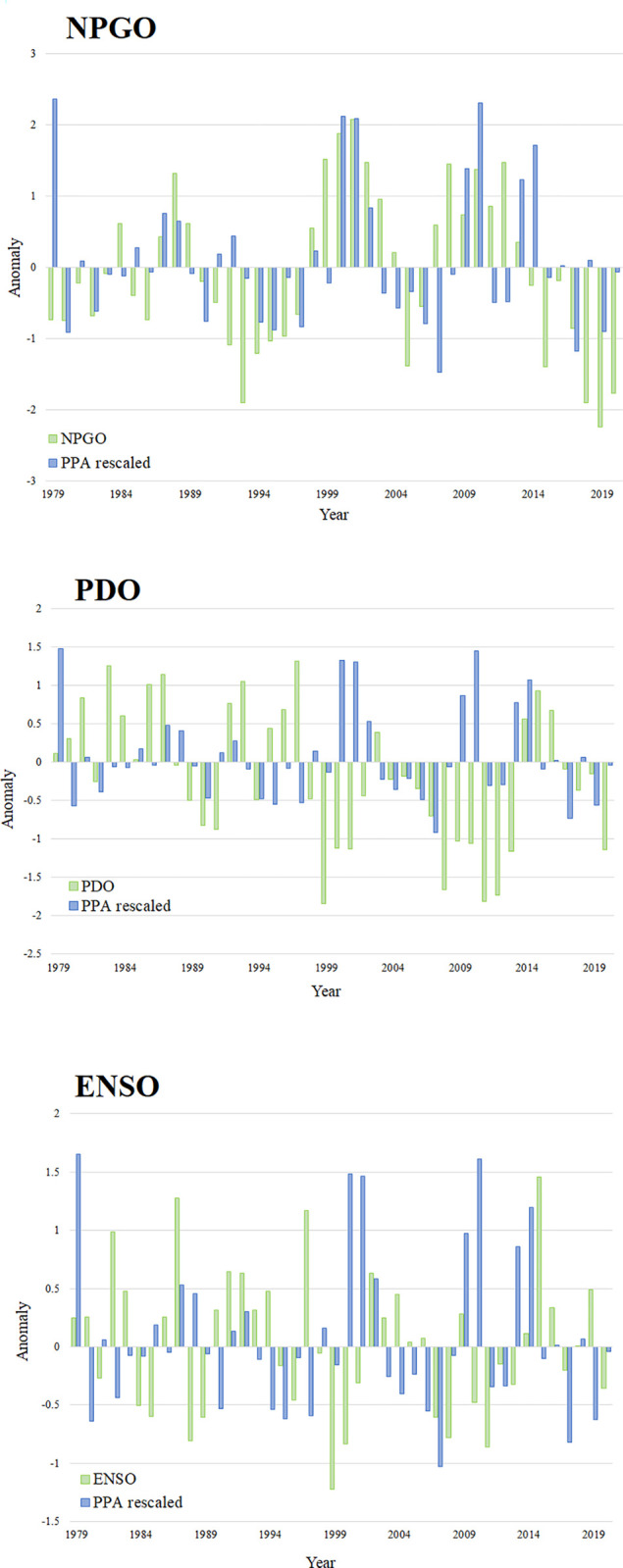
Anomaly indices of relative primary productivity (PPA rescaled) generated through Ecosim, and three large-scale climatic indices NPGO, PDO, and ENSO from 1979 to 2020.

The primary productivity anomalies were estimated as 42 yearly values. These primary productivity anomalies (PPA) estimated by Ecosim showed an alternation of positive and negative fluctuations throughout the time series. These ‘oscillations’ lasted from one to six years (Fig 12). This primary productivity function extracted from Ecosim showed oscillation patterns that were very similar to the NPGO anomalies indices, with 25 years of the time-series being either positive or negative for both functions (Fig 12). The primary productivity anomaly function and the climatic patterns ENSO and PDO simultaneously showed negative or positive values for only 21 years and 19 years, respectively (Fig 12). Clear periods of overall negative (i.e. 1993–1997, 2015–2020) and positive (i.e. 1998–2002) anomalies were detected by Ecosim for primary productivity, all of which matched the timing and range of oscillations observed for NPGO (Fig 12).

## Discussion

Our predictions of changes in biomass of the different marine mammal populations are in line with previous literature and recent population surveys [1, 21, 22, 24, 89, 95, 96]. The Chinook and coho salmon biomass estimates predicted by the model are also aligned with the mostly declining population trends suggested by CWT recovery data [80]. The main objective of this research was to quantify how predation mortality associated with growing marine mammal populations may have limited those salmonid populations so as to potentially reduce food availability for SRKW since 1979. To answer this question, it is important to understand which other sources of mortality, such as fishing, could have limited Chinook and coho salmon populations.

Since 1979, the fishing mortality on *Returning spawners* Chinook salmon was drastically reduced for all groups, with decreases ranging from -37% (WCVI) to -84% (FRGSPS FA). These declines in fishing mortality resulted from numerous conservation management plans and fishing restrictions implemented over the years, such as the Pacific Salmon Treaty (1985, [80]). Severe restrictions in gear, spatial and temporal closures, and fish quotas were gradually applied to both recreational and commercial fisheries over the years. For instance, troll fisheries in British Columbia can now only operate a few weeks per year in specific areas, and some years there are hardly any openings, even for bread-and-butter fisheries such as notably for sockeye salmon [40]. Similarly, recreational fishing is banned from some regions of the Fraser River in the spring and fall, and strict gear, fish size, and number restrictions apply during the rest of the year [40, 97]. Similar extensive regulations have been applied to coho salmon over the last 40 years, with fishing restrictions occurring in Washington, Oregon, and California from the early 1990s and in British Columbia from between 1996 and 1998 [98]. Despite those severe fishing regulations on both Chinook and coho salmon, the model suggests that the total mortality of those groups has not significantly decreased between 1979 and 2020.

During that time, estimated predation mortality increased for all Chinook and coho salmon *Returning spawners* and *Smolts* stanzas included in the model. Large pinnipeds (i.e. California and Steller sea lions) and NRKW appear to be the most important predators of *Returning spawners* Chinook salmon in recent years. The model suggests that the predation mortality of Steller and California sea lions on those Chinook salmon groups in 2020 ranged from 0.05 to 0.3, which would represent an annual consumption of about 0.7 and 0.8 million fish, respectively. Those estimates are in line with the results presented by Chasco et al. (2017) for the year 2015 [9]. Further, the model predicted that the overall predation mortality associated with NRKW on *Returning spawners* Chinook salmon doubled between 1979 and 2020. The intensifying predation by those marine mammals on mature fish can affect the average body size, maturation timing, and life-history characteristics of the Chinook salmon populations through the selective removal of larger fish [99]. In addition, leaving smaller Chinook salmon implies a reduction in caloric value, which could further affect other marine mammals feeding on similar preys, including SRKW [100, 101]. Although one could argue that SRKW might have access to larger fish the same way NRKW do, recent evidence suggests that SRKW might exhibit different foraging strategies than NRWK [102]. When compared to NRKW, SRKW females were found to be less efficient at capturing prey [102]. In this context, the size-selective predation by growing populations of more efficient hunters could directly affect the availability of high-quality prey for SRKW. In addition, no published study yet exists on the potential behavioral avoidance of SRKW when in presence of other marine mammals. Saulitis et al. (2000) observed aggressive non-predatory interactions between Steller sea lions and *resident* killer whales [103]. During their study, Steller sea lions were reported to charge and nip at both *transient* and *resident* killer whales.

The model suggested that the growing population of harbor seals could have been a primary cause for the increased mortality of FRGSPS FA Chinook salmon and coho salmon *Smolts* between 1979 and 2020. Estimated predation mortality by harbor seals on coho salmon *Smolts* increased by a factor of 6.4 between 1979 and 2020, against 4.6 for FRGPS FA *Smolts*. This result is in line with numerous recent studies [9–11, 104] suggesting considerable overall consumption of Pacific salmon smolts by harbor seals in recent years. Most fall-run Chinook salmon exhibit an ocean-type juvenile life-history and spend their first year rearing in the marine environment, potentially making them highly vulnerable to predation during this residency time [105]. In addition, fall-run Chinook smolts are known to grow at a faster rate than their spring- and summer counterparts [106]. In this context, it is possible that harbor seals exhibit a positive selection rather than an opportunistic behavior by targeting more profitable prey with higher calorie value. In addition, the hatchery contribution to the total production of FRGSPS FA smolts in the Salish Sea between 1979 and 2020 ranged from about 33% to 61%. Most hatchery rearing environments do not allow conditioning of young salmon to respond to predator stimuli, therefore potentially increasing their vulnerability to predation after release [107, 108].

In 2020, the model predicted that harbor seals could have consumed about 27 million Chinook salmon smolts in the Salish Sea alone, while Chasco et al. (2017) estimated that consumption at about 23.2 million in 2015 [9]. Further, our study suggested that harbor seal could have consumed about 47 million coho smolts in 2020. Thomas et al. (2017) indicated that harbor seals could consume about 5.7 million coho smolts in one month only in the Strait of Georgia, and that coho smolts were part of the harbor seal diet for at least 8 months of the year [11]. In both cases, the consumption of Chinook and coho smolts by harbor seals predicted by the model is uncertain, as the seasonal and spatial overlap between harbor seals and their prey is not considered in these computations. In order to more precisely quantify the consumption of Pacific salmons by marine mammals, it is essential to acknowledge the highly complex life cycle and seasonal movement patterns of Pacific salmonid species. Efforts should thus be made in future modeling to integrate information on the seasonal and spatial overlap of the main predators and their prey.

The high level of juvenile salmon mortality [109, 110] and declining smolt-to adult survival [111, 112] for Chinook and coho salmon have been well documented. Along with other reports [11, 113], our study suggests that the predation mortality by harbor seals could strongly contribute to the poor survival of those fish in the early marine phase of life. In asking the question of competition between marine mammals, one could argue that harbor seals and SRKW do not target Chinook salmon of similar age. However, a dramatic reduction of survival in the early life could directly result in a lower number of adult individuals available to fisheries and larger predators [111, 114, 115]. In this context, harbor seal could indirectly compete with SRKW by lowering the prey biomass available to the whales at a later stage.

Despite our findings, knowing with certainty whether harbor seal predation on juvenile salmon is additive or non-additive remains challenging and is an important limitation of the model. Non-additive mortality occurs if a prey becomes more vulnerable to predation because of external stress, physiological, or behavioral factors, yet would die even if not eaten by a predator [116]. Additive mortality would occur if each mortality sources were independent from each other [116, 117]. In the case of Chinook salmon, Mesa et al. (1998) suggested that juvenile fish infected with disease agents were significantly more vulnerable to predation than healthy individuals [118]. Other studies conducted on Chinook [119], coho [120], and steelhead [121] salmon suggested that pathogens could be related to lower growth rates, fitness indices, potential chances of successful migration, and overall survival. Besides, Walters and Christensen (2019) suggested that Chinook smolts could still be exhibiting high stress-dependent mortality rates even if seal populations were reduced [116]. Taking into consideration the geographical overlap between the different marine mammal populations may also be of critical importance when examining how marine mammal competition might be limiting for the SRKW population. This is especially needed given that the geographical range of marine mammal populations may well be changing over the coming decades. For instance, Edgell and Demarchi (2012) suggested that in recent years sea lions have been occurring more frequently and in higher numbers along the coast of Washington State and British Columbia [122]. Spatial integration should be recognized as a key aspect of future model examining the evolution of inter-specific competition between highly mobile predator and prey populations.

The model did not show a clear association between variations in primary productivity and changes in coho and chum salmon population trends. On the other hand, the model suggested that primary productivity anomalies linked to the North Pacific Gyre Oscillation could influence the productivity of Pacific Chinook salmon populations from British Columbia to California. This finding is of critical importance, and highlights the potential correlation between large-scale climatic indexes and the productivity of separate Chinook salmon stocks. Di Lorenzo et al. (2008) showed that the NPGO was correlated to significant changes in sea surface temperature, salinity, as well as in nutrient and chlorophyll-a concentrations [87]. Increased sea surface temperatures have been shown to influence salmon juvenile growth rates [123], prevalence to diseases [124], mortality [125], and productivity [126, 127] of different Pacific salmon species. In addition, studies in the north Pacific showed that sea-surface salinity could negatively affect the growth [128] of chum salmon. In the model, peaks in abundance of all Chinook salmon groups were observed in years where anomalies in primary productivity and NPGO were positive (i.e. early 2000s, and after 2010). These results are in line with several recent studies [31, 129, 130], which have concluded that a significant correlation exist between the NPGO and the productivity of several Pacific salmon populations. For instance, Debertin et al. (2017) observed that a positive NPGO was associated with an increased primary productivity and growth in chum salmon [129]. Kilduff et al. (2015) further suggested that the NPGO could be the most important climatic driver of coho and Chinook salmon survival along the northeast Pacific coast, which is also supported by the results of the model [31]. Our study emphasizes the need to consider large-scale changes in climatic conditions and primary productivity patterns when aiming to understand the causes for changes in Pacific salmon population trends. Finally, it is important to note that while the main research question focused on the impact of marine mammal predation on Pacific salmon populations, the use of an ecosystem model allowed for investigating both top-down and bottom-up driving forces in the ecosystem. Important future research should aim at understanding how different management measures could impact the state and evolution of this ecosystem in the coming decades.

## Supporting information

S1 Appendix (DOCX)

S2 Appendix (DOCX)
