# Supplementary material for: The combined effects of predation, fishing, and ocean productivity on salmon species targeted by marine mammals in the northeast Pacific
Source: PLoS One. 2024 Mar 14;19(3):e0296358. doi: 10.1371/journal.pone.0296358 (PMC10939214; doi:10.1371/journal.pone.0296358)
Supplement: S1 Appendix — (DOCX) [file pone.0296358.s001.docx]

**Supporting information S1 - Appendix A**

**SECTION 1: FUNCTIONAL GROUP ECOPATH PARAMETERS**

##### Transient killer whales (**TKW**)

Mammal-eating *transient* killer whales (*Orcinus orca*), also referred to as *Bigg’s* killer whales, were identified as a distinct ecotype in the early 1970s [1, 2]. This ecotype is divided into three subpopulations over the northeast Pacific region: the Gulf of Alaska, the West Coast, and the AT1 [3], and was listed as *threatened* under the COSEWIC (Committee on the Status of Endangered Wildlife in Canada) in 2001 and the SARA (Species at Risk Act) in 2003 [4]. The West Coast *transien*t killer whale population inhabits the coastal waters of British Columbia and is divided into two subpopulations: the ‘inner coast’ and the ‘outer coast’ *transient* killer whale population [5]. The first subpopulation is composed of about 300 individuals, and is mainly found in the inshore waters of the Salish Sea and along the coastal waters of the west coast of Vancouver Island and Haida Gwaii. The ‘outer coast’ population comprises about 200 individuals and is believed to commonly occur in deeper waters closer to the continental shelf [5]. Given the coastal focus of our study area, only the ‘inner coast’ subpopulation of *transient* killer whales was included as a functional group in our model, and was referred to as TKW.

The 1979 TKW population abundance was obtained from Ford et al. (2007), and estimated at 22 individuals [1]. No information was found on the average body growth pattern of female or male TKW, so the 1979 biomass for this functional group was estimated using the average weight of a *resident* killer whale (i.e. 2998 kg) [6]. This approach was deemed reasonable as *transient* and *resident* killer whales exhibit only slight morphological differences, mainly regarding the shape of the dorsal fin [7] and the pigmentation of the saddle patch [8]. TKW are known to occur from northern California to the extreme northern border of southeast Alaska, and are thus likely to occur outside the modeled area during specific times of years [5]. This overall range represents a linear distance of about 2,000 km along the coast, while the model extends over 1,400 km [1, 5, 9]. The initial biomass estimate for TKW in our model area was thus multiplied by 0.7, and estimated at 0.00018 t/km^2^. In addition, it is believed that TKW used to spend only the summer months (~3 months) in our model area, so this estimate was further divided by 4 and adjusted to 0.000046 t/km^2^. To calculate P/B, the average survival-at-age was extracted from Ford et al. (2007) for calves, juveniles, females, sub-adult males, and males [1]. Here, we assumed that sub-adult males were individuals ranging from 15 to 20 years old [1]. The average longevity for the TKW population in 1979 was 22.5 years, so the P/B for this groups was estimated at 0.044 year^-1^. To calculate Q/B, we used the average field metabolic rate presented by Williams et al. (2004) for marine mammal-eating killer whales [10]. According to this study, an adult TKW would need about 172,374 kilocalories daily. Based on an energetic density of about 2.534 kcal. g^-1^, this prey consumption would be the equivalent of a killer whale consuming about 1.36 Steller sea lion pups of 40 kg per day [10].Following this assumption, we estimated that the average daily food intake for a 2298 kg TKW was about 68 kg, and determined that the consumption/biomass ratio (Q/B) for this group was about 11.08 year^-1^.

##### Southern resident killer whales (**SRKW**)

The number, estimated age, and sex of every individual in the SRKW population is known for every year since the early 1970s [11]. In 1979, it is estimated that the population was 78 individuals. The overall biomass of this functional group was calculated by adding the estimated mass-at-age of every individual present in the population that year. Those calculations are detailed in the method section of Couture et al. (2022) and based on body growth rate estimates from Noren et al. (2011) [6, 12]. Overall, the average individual weight was estimated at 2998 kg, and the overall biomass at 0.00084 t/km^2^. The estimated annual survival-at-age reported by Robeck et al. (2015) was used to estimate the average longevity and P/B of the SRKW population [13]. We found that the average longevity for this population was only 19.1 years, yielding a P/B of 0.052 year^-1^.

To calculate the food consumption of SRKW, we calculated the average daily prey energetic requirements (DPER) of this functional group for the year 1979. The detailed method for those calculations can be found in Couture et al. (2022). The average DPER was then split according to the relative diet proportion and weight-at-age of the three most important prey species for SRKW between May and October: Chinook, chum, and coho salmon [14]. Overall, it was estimated that the average daily weight eaten per whale was about 84 kg, giving a yearly Q/B of 10.17. Finally, it is estimated that the SRKW population was growing at an annual rate of about 1.3% between 1974 and 1987, which also corresponds to half of the estimated maximum intrinsic rate of increase for this population [15, 16]. The initial biomass accumulation rate (BA) was thus set at 0.013 year^-1^.

##### Northern resident killer whales (**NRKW**)

The NRKW population is the other *resident* population found in the waters around British Columbia in the summer. Similarly to the SRKW population, this functional group has been closely monitored since the early 1970s [11]. It was estimated that 140 individuals occurred in the population in 1979, yielding a biomass estimate of 0.0017 t/km^-2^. The estimated annual survival and mortality rates at-age reported by Olesiuk et al. 2005 were used to estimate the average longevity and P/B of the NRKW population [17]. We found that the average longevity for this population was about 25 years, yielding a P/B of 0.0416 year^-1^. We used the estimates of average individual body weight and Q/B computed for SRKW as a proxy for NRKW, as both *resident* populations are generally considered similar in term of body size and morphology. It was estimated that the average daily weight eaten per whale was about 84 kg, giving a yearly Q/B of 10.17 year^-1^. Finally, it is estimated that the NRKW population was growing at about 2.6% between the 1970s and the 1990s, which also corresponds to the maximum intrinsic rate of increase for this population [15, 18]. The initial biomass accumulation rate (BA) was thus set at 0.026 year^-1^.

##### Dolphins and porpoises

The exact status of most dolphin and porpoise populations occurring along the northeast Pacific coast and British Columbia is unknown, as it is hard to obtain accurate abundance estimates for species with wide geographical ranges [19, 20]. The three most common species for the modeled area included harbour porpoises (*Phocoena phocoena*), Pacific white-sided dolphins (*Lagenorhynchus obliquidens),* and Dalls’s porpoises (*Phocoenoides dalli*). Gaskin (1992) estimated that 15,000 to 20,000 harbour porpoises were inhabiting western Canadian waters, but this estimate is uncertain [21]. Overall, little is known on the overall historic and current population trend for this species [22]. In 1996, it is believed that the population occurring in the coastal waters of the Salish sea was about 15,000 individuals, while the population inhabiting the coastal waters along the Oregon and Washington coasts comprised about 40,000 individuals in 1997 [22, 23]. No estimates could be found regarding the size of the harbor porpoise population in the late 1970s. Dall’s porpoises and Pacific white-sided dolphins are sometimes seen interacting and travelling together in the wild [20]. Dall’s porpoises are found in the coastal and pelagic waters of the north Pacific Ocean, from the west coast of California to the Sea of Japan [24, 25]. Further, Pacific white-sided dolphins are amongst the most abundant dolphin species in the north Pacific, and their range extends from the southern tip of the Baja Peninsula (Mexico) to the Aleutian Islands in Alaska [26, 27]. No information could be found on the status of either population, as their movement and migratory patterns are still poorly understood.

Preikshot (2007) developed an Ecopath model for the year 1950, and estimated that the overall biomass of odontocetes for British Columbia and the northeast Pacific coast was 0.036 t/km^2^ [28]. Harbour porpoises, Dall’s porpoises, and Pacific white-sided dolphins together represented 34.8% of this functional group. We used this estimate for our model, and set the overall biomass to 0.0125 t/km^2^. Regarding the Q/B ratio, the average of the three calculations for each species was taken as a proxy for this functional group. Harbour porpoises have an average adult weight of 52.5 kg, and research on individuals in captivity showed that they could consume about 8.5 % of their body mass (i.e. ~ 4.5 kg.day^-1^) [21, 29, 30]. The Q/B for this species was thus estimated at 31.025 year^-1^. Dall’s porpoises have a maximum daily food intake of about 5.04% of their body weight, and adult individuals could weigh about 136 kg, yielding a Q/B estimate of 18.3 year^-1^ for this species [31]. Further, Pacific white-sided dolphins have very high energetic needs, and might consume up to 18% of their body weight daily [32]. With an average adult weight of 90 kg, the Q/B for this species was set at 65.7 year^-1^ [26]. Overall, the Q/B ratio for this functional group was evenly averaged between all three species and set at 38.37 year^-1^. Finally, the P/B ratio of Pacific white-sided dolphins was taken as a proxy for the whole group, as no survival-at-age data were found for the two other species. According to the life tables presented by Heise (1997), the P/B was estimated at 0.3 year^-1^[26].

##### Harbor seal (**HS**)

Harbor seals have been protected under the Marine Mammal Protection Act since 1972, and have been growing exponentially in British Columbia between the early 1970s and 1990 at an annual rate of 12.5% [33]. It is thought that the average weight of an adult harbor seal is about 80 kg (male; 84.6±2,1 kg, female:76.5±3 kg) [34]. However, there is a need to consider the overall age- and sex- structure of the harbor seal population in 1979 to achieve a more realistic estimate of the overall biomass at this time. Previous population parameters and sex-specific weight-at-age estimates presented by Olesiuk (1993) were used to calculate the average individual weight (i.e. 44.7 kg) of harbor seal in the Strait of Georgia [33]. This individual weight estimate was used to calculate the initial biomass of this group for the year 1979, along with abundance estimates in British Columbia [35], Puget Sound [36], and Washington and Oregon coasts [36, 37] for this year. Data on the status of harbor seals in California were scarce, and only covered the period 1982-1995 [38]. In addition, harbor seals occurring in California represent less than 10% of the overall northeast Pacific population, while about 40% occur in British Columbia only [38, 39]. Based on those estimates, it was deemed reasonable to exclude the Californian portion of the population from our calculations. The biomass estimate for our model area was 0.0863 t/km^2^. To calculate the P/B value for this group, we first used the life tables (survival-at-age) and weight-at-age reported by Olesiuk (1993), allowing us to estimate the biomass-at-age (i.e. average weight multiplied by numbers at-age) and mortality (i.e. average weight multiplied by annual mortality loss) [33]. Those calculations yielded a mortality rate of 0.098 year^-1^. As the finite growth rate of increase for harbor seal was previously estimated at 1.12, the overall P/B for this group was set at 0.212 year^-1^ [33]. Finally, Olesiuk (1993) estimated that the average daily food consumption of an adult harbor seal individual was about 1.9 kg, so the yearly consumption/biomass ratio Q/B for this group was estimated at 15.5 year^-1^.

##### Steller sea lion (**SSL**)

To estimate the average weight of a Steller sea lion individual, we used the relative proportion of different age groups (i.e. yearlings (20%), juveniles (36%), adult females (33%), and adult males (11%)) presented by Olesiuk (2018) for the population, along with the average weight-at-age recorded by Winship et al. (2001) [40, 41]. Overall, it was estimated that the average individual weight for this functional group was 279.8 kg. The historical trends in abundance of Steller sea lions in British Columbia were extracted from 1979 to 2017 [40, 42]. Unlike harbor seals and California sea lions, Steller sea lions did not increase right after the protection of their population in 1972, showing significant population increases only from the early 1990s onwards [40]. In 1979, it was estimated that about 5,590 individuals were present in British Columbia, yielding a biomass of 0.0053 t/km^2^ for that year. Winship et al. (2002) estimated that the average food consumption requirements for a mature (> 10 years old) female and male Steller sea lion were about 17 ($\pm3.8)$kg.day^-1^ and 33 ($\pm7.4)$ kg.day^-1^, respectively [43]. On the other hand, yearling and juvenile Steller sea lions could consume 13.37 kg and 19 kg of food daily, respectively [43]. Using an average daily prey consumption of 18.75 kg and an adult body weight of 279.87 kg, the consumption/biomass ratio Q/B for this functional group was set at 24.45 year^-1^ [41]. Estimates of survival-at-age were extracted from Olesiuk (2018) and Winship et al. (2002), along with estimates of weight-at-age extracted from Winship et al. (2001) [40, 41, 43]. The total mortality Z was then calculated from the biomass- and mortality- at-age estimates, and added to the intrinsic rate of increase of the population (i.e. 0.061). Altogether, the P/B for this group was estimated at 0.162 year^-1^. Finally, the Steller sea lion population was increasing at a rate of about 4.8% per annum between 1971 and 2013, so the biomass accumulation rate was set at 0.048 year^-1^ for this group.

##### California sea lion (**CSL**)

Five genetically distinct populations of California sea lions occur along the northeast Pacific coast, including the United States population [44]. This population breeds on offshore islands in California but its overall habitat ranges from the USA-Mexico border to southeastern Alaska [44]. Female California sea lions are considered non-migratory, and stay around the rockery islands throughout the year, whereas adult and non-reproductive males visit the coastal waters of Oregon, Washington, and British Columbia during the non-reproductive season [44, 45].

The overall abundance of this population has been increasing steadily since their protection in 1972, and counted about 92,812 individuals in 1979 [44]. For this model, we assumed a 1:1 female-male ratio, hence leaving half of the population outside of our model area. In addition, our model area represented only about 45 % of the total range of male California sea lions, yielding an overall biomass of 0.0229 t/km^2^ for this functional group in 1979. This calculation was based on the average asymptotic weight of an adult male, which was estimated at about 400 kg [46].

There is a paucity of information regarding the food consumption of otariids in the wild, and most information found on California sea lion concerned female individuals [47] or captive individuals in other regions of the world [46]. Here, we used the annual consumption estimate (i.e. about 18.5 kg/day per adult male) reported by Weise and Harvey (2018) [48]. We calculated that the yearly Q/B for that group was about 16.89 year^-1^. In addition, we used the age- and sex- specific survival rates presented by Delong et al. (2017) for the San Miguel Island in the California Channel Islands to estimate the average longevity for this group at about 14.2 years [49]. The intrinsic growth rate of the population was calculated from population growth data presented in Laake et al. (2018), and estimated at 0.073 [44]. Altogether, the P/B for this functional group was thus set at 0.144 year^-1^. Finally, it is believed that the population grew up at an annual rate of about 5.4 % between 1975 and 2008 [50].

##### Seabirds

This functional group included two different species considered the most abundant in our model area: the common murre (*Uria aalge*) and the rhinoceros auklet (*Cerorhinca monocerata*). About 1,034,600 common murres are known to inhabit the coastal region between northern California and British Columbia [51]. Those estimates were mainly given between 1979 and the early 1990s, and were used as proxies for our Ecopath model. Adult common murres weigh about 1 kg, so the biomass for this species was 0.0042 t/km^2^ [52]. Rhinoceros auklets are known to breed from the northern tip of Vancouver Island all the way to the Aleutian Islands in Alaska [53]. They are usually found further south during wintering periods, all the way to the Baja Peninsula in Mexico [54]. With a population of about 750,000 individuals and an average adult weight of 0.52 kg, the biomass for this species was estimated at 0.0016 t/km^2^ for our model area [54]. Overall, the biomass for this functional group was set at 0.0058 t/km^2^. Manuwal et al. (2001) reported that average survival rate for adult common murres ranged between 0.87 and 0.94, while Bertram et al. (2000) estimated it at 0.82 for adult rhinoceros auklets [51, 55]. The average survival rate between both species (i.e. 0.8625) was used to determine P/B, which was estimated at 0.1375 year^-1^. Although food consumption varies with breeding status, adult common murres consume an average of 350 g of food daily, which represents about 33% of their body weight [56, 57]. This estimate yielded a yearly Q/B of 127.5 year^-1^ for this species. Also, rhinoceros auklets consume about 59 g of food per day, so the Q/B for this species was set at 59.65 year^-1^[58]. The average Q/B between both species was 93.7 year^-1^, and was taken as a proxy for the entire functional group.

##### Chinook salmon

###### Stocks

Chinook salmon stocks were divided into seven groups according to their region of origin and the dominant timing of the adult runs. In total, 32 stocks were identified by the Joint Chinook Technical Committee of the Pacific Salmon Commission, and all are believed to be reliable escapement indicator stocks for Chinook salmon populations in British Columbia and along the coastal waters of Washington and Oregon states. In total, 16 Chinook salmon populations originating from the Salish Sea (i.e. Fraser River, Strait of Georgia, and Puget Sound, referred to as FRGSPS) were divided into three seasonal runs (i.e. spring, summer, and fall) based on their dominant life history trait and run timing. Based on the stock descriptions presented in the 2019 exploitation rate analysis and model calibration report of the Technical Chinook Committee [59], FRGSPS Spring (SP) included FS2 (Fraser Spring 1.2), FS3 (Fraser Spring 1.3), and NKS (Nooksack Spring ‘yearling’) (CTC 2019). FRGSPS Summer (SU) included four stocks: FSS (Fraser Summer 0.3), FSO (Fraser Summer 1.3), SNO (Snohomish ‘fingerling’), and SKG (Skagit ‘fingerling’). Finally, nine stocks were included into the FRGSPS Fall (FA) functional group: FCF (Chilliwack hatchery), FHF (Harrison hatchery), MGS (Middle Georgia Strait), LGS (Lower Georgia Strait), NKF (Nooksack Fall), PSY (Puget Sound hatchery ‘yearling’), PSN (Puget Sound natural ‘fingerling’), PSF (Puget Sound hatchery ‘fingerling’), and STL (Stillaguamish ‘fingerling’). Only two Chinook salmon stocks were included into the west coast of Vancouver Island fall run (referred to as WCVI FA) functional group: WVH (Robertson hatchery) and WVN (WCVI natural). Finally, 14 stocks originating from the Columbia River and the coastal areas of Washington, Oregon, and California States (referred to as CRWORC) were included. The CRWORC Spring (SP) functional group included two stocks (CWS (Cowlitz Spring hatchery) and WSH (Willamette Spring hatchery)) whereas the CRWORC Summer (SU) functional group only included SUM (Columbia River Summers). Further, the CRWORC Fall (FA) group included nine CTC stocks: WCN (Washington Coastal natural), WCH (Washington Coastal hatchery), NOC (north migrating Oregon Coastal), CWF (Cowlitz Fall hatchery), MCB (mid-Columbia Brights), LYF (Lyons Ferry ‘fingerling’), URB (Columbia upriver Brights), BON (Bonneville hatchery), and SPR (Spring Creek). Two other Chinook salmon assemblages originating from the Klamath and Sacramento Rivers (fall runs), for which data on escapement and fisheries catch were retrieved from two reports made available online by the Salmon Technical Team (STT) of the Pacific Management Fisheries Council (2022), were added to this last functional group [60, 61].

###### Stanzas

Each of the seven Chinook salmon functional groups were divided into six sub-groups referred to as ‘stanzas’, which allow to capture ontogenic diet shifts and different age-specific predation and exploitation patterns [62, 63]. Those stanzas were described as *River*, *Smolts*, *Juveniles*, *Marine*, *Returning spawners*, and *Escapees*. The *River* stanza accounted for the number of individuals from birth to 3 months old (for sub yearling fish, mainly summer and fall run type) or 12 months old (for yearling fish, mainly spring type). Those estimates were related to the average smolt age at release reported in by the Joint Technical Committee of the Pacific Salmon Commission [59]. For summer and fall run types (i.e. FRGSPS SU, FRGSPS FA, WCVI FA, CRWORC SU, CRWORC FA), the *Smolts* stanza extended from 3 to 12 months old, whereas the *Juveniles* stanza ranged from 12 to 24 months old. For spring run types (i.e. FRGSPS SP, CRWORC SP), the *Smolts* stanza extended from 12 to 16 months old, whereas the *Juveniles* stanza ranged from 16 to 24 months old. The *Juveniles* stanza was added to account for the proportion of fish which are rearing for longer periods of time in coastal waters and still prone to predation before starting their migration. For instance, fall-run Chinook salmon originating from the Fraser River and the Puget Sound area are known to spend more time rearing in the sheltered coastal waters of the Salish Sea than their spring- and summer- run counterparts [64]. Moreover, spring-type yearling Chinook salmon originating from the Columbia River also tend to show a very rapid northward migration and can travel all the way up to the coastal waters of Alaska [65]. For all groups, the *Marine* stanza (i.e. fish migrating in offshore waters and considered protected from marine mammal predation in our model) ranged from 24 to 36 months old*,* whereas the *Returning spawners* stanza ranged from 36 to 60 months old. The *Escapees* stanzas included fish that were older than 60 months, and mainly allowed to account for fish dying and contributing to the ecosystem’s nutrient cycle. When creating multi-stanza groups in Ecopath, the baseline estimates of total mortality Z need to be entered for each stanza. However, the biomass, Q/B, and bioaccumulation estimates are only needed for one ‘leading’ stanza [62]. In our case, the *Returning spawners* stanza was chosen as the leading stanza, as fisheries and biomass estimates are available and likely more reliable for this group.

###### Parameters

Following the method developed by Ruggerone and Irvine (2018), the biomass of the *Returning Spawners* stanzas of all Chinook salmon functional groups was based on abundance estimates achieved by adding the fisheries catch and escapement numbers [66]. Estimates of total cohort, escapement, and catch by fishery-at-age were provided by the Chinook Technical Committee (CTC) of the Pacific Salmon Commission [59]. All the fisheries used in this model are listed in Table A1 under their CTC acronyms, and are described in more details in the 2021 fisheries report issued by the Joint Chinook Technical Committee of the Pacific Salmon Commission [67]. For the *Returning spawners* stanza, the fisheries catch used to calculate fish abundance excluded fisheries occurring in the northern Pacific and Alaska, as those fish were removed from the system prior to marine mammal predation. Conversely, the fisheries catch used to estimate the fishing mortality (F) of this stanza solely included the fisheries occurring in the model area, and excluded freshwater fisheries. It is important to note that the fisheries were categorized depending on their dominant activity, as some fisheries operate both in marine and freshwater environments [67] (Table A1). Finally, the average weight of a 4-year-old Chinook salmon (i.e. 8.5 kg, [14]) was used to calculate the overall biomass of each *Returning Spawners* stanza, and yielded the following initial estimates: 0.0023 t/km^2^ (FRGSPS SP), 0.0084 t/km^2^ (FRGSPS SU), 0.0616 t/km^2^ (FRGSPS FA), 0.0081 t/km^2^ (WCVI FA), 0.0047 t/km^2^ (CRWORC SP), 0.0028 t/km^2^ (CRWORC SU), and 0.051 t/km^2^ (CRWORC FA). Note that those biomass estimates are different from the ones presented in Table 4 in the manuscript, as those were adjusted when balancing our Ecopath model. Fisheries operating north of our model (including Alaska) were used to estimate the fishing mortality of all Chinook salmon *Marine* stanzas. Finally, it was assumed that only Chinook salmon individuals that had already escape marine fisheries and predation would be vulnerable to freshwater fisheries, and those fisheries catch estimates were thus used to calculate the fishing mortality on all *Escapees* stanzas.

**Table A1.** **List of the fisheries included used to calculate the biomass and fishing mortality of the different Chinook salmon stanzas of our model.**

| **Northern/Alaska**  **Fisheries** | **Fisheries**  **occurring in the model area** | | **Freshwater Fisheries** |
| --- | --- | --- | --- |
| ALASKA T | NORTH T | CBC S | TGS FS |
| ALASKA N | WCVI T | NBC AABM S | TPS FS |
| TBC TBR FN | N FALCON T | NBC ISBM S | TGEO ST FN |
| TAK YAK N | S FALCON T | WCVI AABM S | TSF FS |
| ALASKA S | GEO ST T | WCVI ISBM S | TFRAS FN |
| TAK TBR N | NORTH N | N FALCON S | TPS FN |
| TYK YAK FN | CENTRL N | S FALCON S | TWAC FN |
| TAK TBR S | WCVI N | PGSDN S | TWCVI FS |
|  | J DE F N | PGSDO S | TFRASER FS |
|  | PGSDN N | GEO ST S | TCOL R N |
|  | PGSDO N | BC JF S |  |
|  | WASH CST N | TCOL R S |  |
|  | TCENTRAL FN | TNORTH FS |  |
|  | JNST N | TCENTRAL FS |  |
|  | FRASER N |  |  |

All description of those fisheries acronyms from the Joint Chinook Technical Committee can be found in their 2021 fisheries report [67].

The von Bertalanffy growth model was developed in 1938, and is often used in fisheries science to estimate the growth of a fish as a function of its age. For each Chinook salmon functional group, the annual curvature parameter (K, year^-1^, representing the growth rate) of this growth function needed to be inputted, as well as the weight at maturity (W_mat_)/weight at infinity (W_inf_) ratio. For both variables, we used the estimates of K=0.14 year^-1^ and W_mat_/W_inf_ = 0.186 from FishBase for Chinook salmon in western Canada [68]. In addition, the Q/B ratio needed to be added for each *Returning spawners* stanza, and was estimated at 2 year^-1^ [68].

Finally, the total instantaneous mortality Z was input for each stanza as the sum of the fishing mortality (F) and natural instantaneous mortality (M) [69]. The fishing mortality was assumed to be null before fish were considered mature, and was set at 0 for the *River*, *Smolts*, and *Juveniles* stanzas. Although it is likely that the finite natural mortality-at-age (i.e. fraction of a fish stock that dies over a specific period of time) of Chinook salmon has varied over the years, it is difficult to obtain accurate estimates, as data remain scarce for the different Chinook salmon populations of our model. Therefore, the finite natural mortality-at-age probabilities presented by the Pacific Salmon Commission were used to estimate the instantaneous natural mortality for each stanza [59]. The finite natural mortality rates for age-2, age-3, age-4, and age-5 or older Chinook are 40%, 30%, 20%, and 10% year^-1^, respectively [59]. The finite mortality M is usually expressed in percent and is equal to $1-s,$ where *s* is the survival rate. In contrast, the instantaneous mortality rate *m* (i.e. probability of death over a very short time frame, used in Ecopath) is by convention expressed as an exponential instantaneous coefficient so that $m=-\ln\left( s \right)$ (or $s=\exp(- m)$). Based on those equations, we assumed that fish between 3 and 5 years old constituted the *Returning spawners* stanza, and calculated that the average instantaneous natural mortality *m* for this group was 0.22 year^-1^. Finally, we assumed that the finite natural mortality for the *Juveniles* (i.e. age-2 fish) and *Marine* (i.e. age-2 and age-3 fish) *stanzas* were 0.51 and 0.43, respectively (PSC 2021a). The spawning proportion was set at 1 for the *Returning spawners* and *Escapees* and set at 0 for all other stanzas of these groups.

###### Hatchery forcing functions

All hatchery releases were extracted from the online database of the Regional Mark Processing Center website for Chinook salmon (through the Regional Mark Information System standard reporting) [70]. All results were filtered for both marked and unmarked fish individuals, and extracted by brood year. Data were extracted for different domains, basins, and regions depending on the area of origin of the different Chinook salmon functional groups included in our model. For the FRGSPS area, all the regions from ten basins (i.e. FRTH (Fraser River and Thompson River), JNST (Johnstone Strait), GST (Georgia Strait), NOWA (Northern Washington), SKAG (Skagit River), NPS (Northern Puget Sound), MPS (Mid Puget Sound), SPS (Southern Puget Sound), HOOD (Hood Canal), and JUAN (Strait of Juan de Fuca)) of two regions (British Columbia and Washington State) were extracted. For the CRWORC area, all the regions from 12 basins (NWC (Northern Washington Coast), GRAY (Grays Harbor), WILP (Willapa Bay), NOOR (Northern Oregon Coast), SOOR (Southern Oregon Coast), UPCR (Upper Columbia River), SNAK (Snake River), CECR (Central Columbia River), LOCR (Lower Columbia River), SAFA (Sacramento River, Feather River, American River), NOCA (Northern California Coast), and KLTR (Klamath River) of four basins (Washington State, Oregon State, Columbia River, California State) were extracted. Based on CWT data for Chinook salmon, the typical number of spawners produced by each smolt has been around 0.004, corresponding to an alternative estimate of about 250 smolts per spawner [59]. Based on those estimates, we back-calculated the effective smolt production based on the number of returning spawners three years earlier. Natural recruitment was added to hatchery releases, and overall estimates were then converted to relative releases (in comparison of the Ecopath year 1979) and entered as a forcing function in Ecosim. This production was added as a hatchery stocking forcing function in the multi-stanza editing tool in Ecopath (Figure A1).


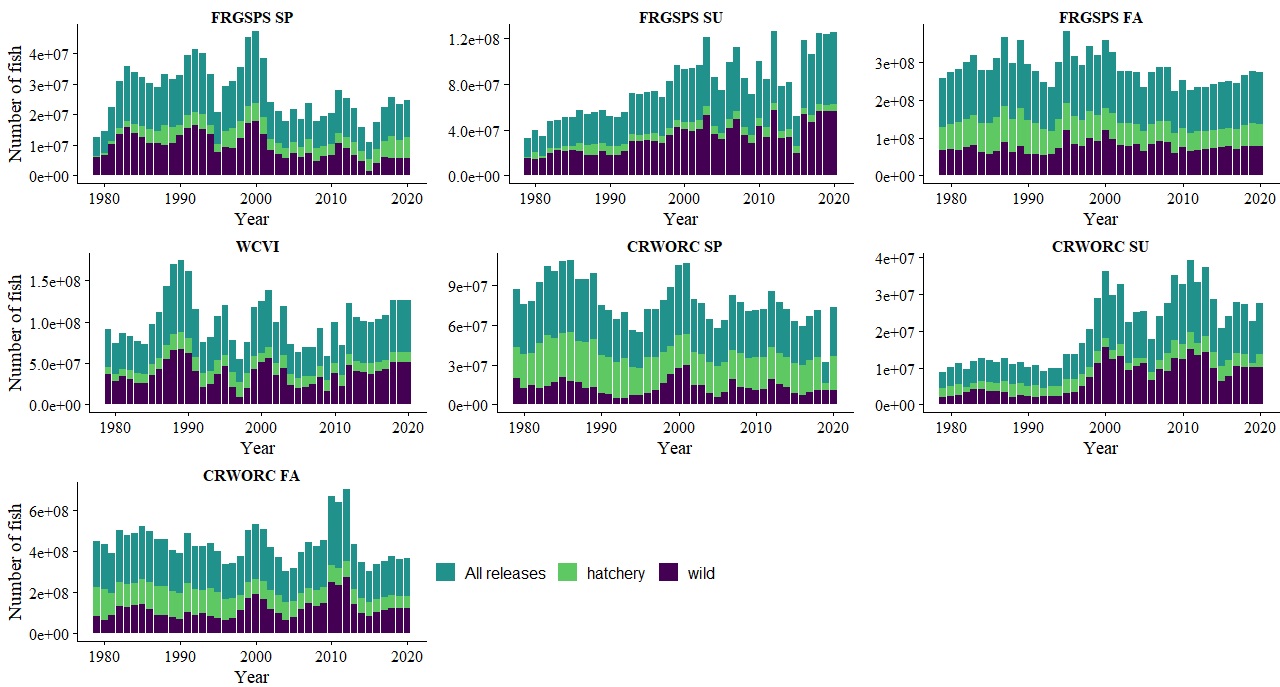


**Figure A1.** **Abundance trends of hatchery releases and estimated wild smolt production for the seven different Chinook salmon functional groups included in the model from 1979 to 2020**.

##### Coho salmon

Coho salmon usually hatch in the spring and spend the first year of their life in freshwater before entering the marine environment in the spring of the following year [71]. In the Strait of Georgia, coho salmon then spend their first summer along protected coastal waters before migrating in the early fall [72]. Overall, coho salmon are known to spend about 16 to 18 months at sea, mainly along the coast of British Columbia, before returning to spawn in their natal freshwater streams [73]. Based on those facts, this functional group was divided into 5 stanzas: *River* (0 to 12 months), *Smolts* (12 to 18 months), *Marine* (18 to 36 months), *Returning spawners* (36 to 48 months), and *Escapees* (more than 48 months). The spawning proportion was set at 1 for the *Returning spawners* and *Escapees,* and set at 0 for all the other stanzas. Based on estimates found in FishBase for this species, the VBGF curvature parameter K was set at 0.2 year^-1^, while the W_mat_/W_inf_ was set at 0.09. The consumption/biomass ratio (Q/B) for the leading stanza *Returning spawners* was also extracted from FishBase and set at 6.49 year^-1^ [68]. The average marine survival of coho salmon in the northeast Pacific have been summarized in several studies [74–77] over the last decades, and ranges from ~ 2.2% to ~ 2.9%. We chose to use the estimates presented by Coronado and Hilborn 1998, as they analyzed the entire coast of British Columbia [75]. We thus assumed that the survival for coho salmon in the marine environment was about 5.2%, yielding an instantaneous mortality rate Z of about 2.9 year^-1^. As most mortality happens in the first few days or months at sea, this mortality rate was not evenly divided between the different marine stages, and the *Smolt*s stanza was assigned most of the mortality (i.e. 2 year^-1^). Catch, escapement, and cohort abundance estimates of coho salmon in the Salish Sea (Fraser River, Strait of Georgia, Puget Sound) were extracted from publicly available assessments presented by the Joint Technical Coho Committee of the Pacific Salmon Commission for the years between 1986 and 2010 (<https://www.psc.org/publications/technical-reports/technical-committee-reports/coho/>). Further, fisheries catch for the years 1979 to 1986 were extracted from the stock status report prepared by Fisheries and Oceans (2002) [78]. Commercial and recreational fisheries targeting coho salmon in the Salish Sea were drastically restricted in the late 1990s, and most catch is now incidental or by First Nation communities for food, social and ceremonial purposes [79]. Abundance and fisheries catch estimates for the Columbia River and the Washington, Oregon, and Californian coasts were extracted from the pre-season report of the Pacific Fishery Management Council (2022) [60]. As no information could be found on the age of the fish caught by fisheries, we assumed that the catch was divided equally between the *Marine* and the *Returning spawner*s stanzas. The initial biomass of the *Returning spawners* leading stanza was computed using the average coho salmon adult weight of 4.5kg [80]. All hatchery releases were extracted from the online database of the RMPC website for coho salmon [70]. All results were filtered for both marked and unmarked individuals, and extracted by brood year. Similarly to Chinook salmon, hatchery releases were converted to relative releases (in comparison to the year 1979) and entered as a forcing function in Ecosim before being added as a hatchery stocking forcing function in the multi-stanza editing tool in Ecopath.

##### Chum salmon

The Fraser River is the largest producer of chum salmon in British Columbia, with runs averaging 2 million of fish between 2000 and 2010 [81]. In British Columbia, chum salmon emerge as fry in the winter and migrate downstream in the early spring [81]. After spending up to 6 months rearing in protected coastal waters, chum salmon migrate to offshore waters of the open northern Pacific Ocean and can be found all the way up to northern Alaskan waters [81, 82]. Chum salmon usually return to their natal freshwater streams after two to seven winters out in the marine environment [83]. Yet, most chum salmon originating from the Fraser River return to spawn between three and five years of age [81]. Based on those facts, this functional group was divided into five stanzas: *River* (0 to 3 months), *Smolts* (3 to 16 months), *Marine* (16 to 36 months), *Returning spawners* (36 to 60 months), and *Escapees* (more than 60 months). The spawning proportion was set at 1 for the *Returning spawners* and *Escapees* and at 0 for all the other stanzas. The production of chum salmon originating from the Columbia River is now considered very low, and was excluded from the model [84].

Escapement and fisheries catch estimates for chum salmon in the interior Salish Sea, the WCVI, Puget Sound, and the Washington coast (Willapa Bay and Grays Harbor) were extracted from the Joint Chum Technical Committee Report produced by the Pacific Salmon Commission for the year 1979 [85]. Chum salmon are the second largest Pacific salmon species, and the initial biomass of the *Returning Spawners* leading stanza was computed using the average chum salmon adult weight of 6.1 kg [86]. Based on estimates found on the online platform FishBase for this species, the VBGF curvature parameter K was set at 0.270 year^-1^, while the W_mat_/W_inf_ was set at 0.214 [68].

The consumption/biomass ratio (Q/B) for the leading stanza *Returning spawners* was also extracted from FishBase and set at 1.4 year^-1^[68]. We used the smolt-to-adult low survival rate of 0.7% (equivalent to an instantaneous mortality rate of 4.9 year^-1^) presented by Bradford (1995) to estimate the total mortality for all marine stanzas [74]. Several studies [87–89] suggested that the mortality of most Pacific salmon species might be higher in the early marine phase and often associated with the size and age of the fish. Based on those observations, most of the mortality was assigned to the *Smolts* stanza (i.e. 2.5 year^-1^). All hatchery releases were extracted from the online database of the RMPC website for chum salmon [70]. All results were filtered for both marked and unmarked individuals, and extracted by brood year. Hatchery releases were then converted to relative releases (in comparison to the year 1979) and entered as a forcing function in Ecosim before being added as a hatchery stocking forcing function in the multi-stanza editing tool in Ecopath.

##### Other salmonids

The functional group ‘Other salmonids’ included two other known Pacific salmon species: pink (*Oncorhynchus gorbuscha*) and sockeye (*Oncorhynchus nerka*) salmon. For both species, escapement data for North America were taken as a proxy for our model area and were extracted directly from Ruggerone and Irvine (2018) [66]. Catch statistics were accordingly extracted from the online public database Salmonid Catch Statistics provided by the North Pacific Anadromous Fish Commission (NPAFC, [90]) for both sport and commercial fisheries in British Columbia, Alaska, and the Yukon territory. The average weights for pink salmon (i.e. 1.7 kg) and sockeye salmon (i.e. 2.7 kg) were averaged to yield a biomass of 1.57 t/km^2^ for the year 1979 [66]. Following the method described by Preikshot (2007), the P/B ratio for pink and sockeye salmon were set at 1.4 year^-1^ and 1.27 year^-1^, respectively [28, 91]. Pink salmon and sockeye salmon represented about 59% and 41% of this functional group (in numbers), yielding an average P/B of 1.34 year^-1^. The Q/B for this group was set at 3.8 year^-1^ [68].

##### Herring

Pacific herring (*Clupea pallasii*) is known as a important species, supporting essential ecosystem trophic relationships as well as First Nation and commercial fisheries in the northeast Pacific Ocean [92, 93]. Distinct life stages of this species are known to support different populations of piscivorous predators, such as marine mammals [33, 94, 95], seabirds [96], and larger fish [97–99]. For instance, primary predators of age-0 herring in the Salish Sea include Pacific hake as well as juvenile coho and Chinook salmon, while adult herring are largely preyed upon by humpback whales and pinnipeds [33, 99–101]. In British Columbia, Pacific herring spawn in bays and inlets between February and April [102]. They usually migrate offshore at the end of their first summer, and often reappear along the coastline as mature spawning adults after 2 years [102]. Based on those facts, this functional group was split into two stanzas: *Juveniles* (0 to 24 months), and *Adults* (more than 24 months old). The spawning biomass estimates for herring were extracted from Thompson et al. (2017) for British Columbia (Haida Gwaii, Prince Rupert District, Central coast, Strait of Georgia, WCVI) and the states of Washington (Quartermaster Harbor, Port Gamble, Port Susan, Discovery Bay, Fidalgo Bay, Cherry Point, South Puget Sound, Hood Canal, Whidbey, Olympic, Anacortes, Whatcom), and Oregon (Yaquina Bay) [103]. Data were extracted for the period extending between 1979 and 2020 and converted into the correct unit for our model, yielding an initial biomass estimate of 1.47 t/km^2^. Data on the total landings of Pacific herring were scarce and considered small for California and Oregon States, and solely computed for the main fisheries in British Columbia [104, 105] and Washington State [106]. The natural mortality for fish aged 0 was estimated at 0.5 year^-1^, whereas the natural mortality for fish at maturity was set at 0.4 year^-1^ [68]. A fishing mortality estimate of 0.256 year^-1^ was obtained for the *Adults* stanza in 1979, yielding an overall P/B of 0.65 year^-1^. The VBGF curvature parameter K and the W_mat_/W_inf_ ratio were both set at 0.190 for this group [68].

##### Other functional groups

Other functional groups were added based on their relative importance in the diet of the other focal species of this research. Asides from salmonid species, SRKW are known to feed on Pacific lingcod, Pacific halibut, and different rockfish species (*Sebastes* spp) [107, 108]. Pacific lingcod is known to occur from Alaska to California, but is highly concentrated near the coast of British Columbia, especially around Haida Gwaii, Queen Charlotte Sound, Vancouver Island, and the Strait of Georgia [109]. For this reason, the estimated median stock biomass in British Columbia was used as a proxy for the entire functional group throughout the model area, and estimated at 0.05 t/km^2^ [109]. The fishing mortality was estimated from the total coastwide fishing estimates provided in the lingcod assessment report from Fisheries and Oceans Canada (2011) and set at 0.23 year^-1^ [109]. The consumption ratio Q/B was set at 1.7 year^-1^ whereas the natural mortality was estimated at about 0.31 year^-1^, yielding an overall P/B ratio estimate of 0.54 year^-1^ for this species [68].

Pacific halibut are known to occur from the Bering Sea all the way down to central California, but are most common in the central Gulf of Alaska [110]. Besides, about 83% of the Pacific halibut population that can be fished is found in Alaskan waters [110]. The 1979 total biomass estimates for adult Pacific halibut were calculated by Sullivan et al. (1999) from northern California to northern Haida Gwaii, and estimated at about 60 million pounds, or about 0.11 t/km^2^ [111]. For the same year, about 4.91 million pounds were harvested by commercial fisheries in this area, yielding a fishing mortality estimate of 0.127 year^-1^. The consumption ratio (Q/B) was set at 1.85 year^-1^ whereas the natural mortality was estimated at about 0.08 year^-1^, yielding an overall P/B ratio estimate of 0.2065 year^-1^ for this species [68].

There are at least 64 species of rockfish occurring between Alaska and central California, about 35 of which are found in British Columbia [112]. The consumption and production ratios of this functional group were averaged between the seven most common species found in our model area (i.e. copper rockfish (*Sebastes caurinus*), Puget Sound rockfish (*Sebastes emphaeus*), Quillback rockfish (*Sebastes maliger*), black rockfish (*Sebastes melanops*), China rockfish (*Sebastes nebulosus*), tiger rockfish (*Sebastes nigrocinctus*), and yelloweye rockfish (*Sebastes ruberrimus*)), and set at 3.11 year^-1^ and 0.36 year^-1^, respectively [68, 112]. Because of the diversity of species included in this functional group, an accurate calculation of the biomass was difficult, and the estimate given by Preikshot (2007) for the continental shelf of British Columbia was used (i.e. 1 t/km^2^) [28].

Pacific hake (*Merluccius productus*) is an important groundfish species found across both west Canadian and American waters, and one which all the pinniped species included in this model prey upon [33, 113]. Pacific hake is considered a highly migratory species, as fish spend most of the years in offshore waters before migrating in the spring towards the coastal waters of the northeast Pacific to spawn [114]. In the scope of this model, this functional group will only refer to the coastal stock which occur from northern Haida Gwaii to southern California [114]. Overall, Pacific hake is considered the most important ground fish species throughout its range [114]. It is believed that the population biomass of the coastal stock of Pacific hake in Canada and the USA in 1979 was about 1.6 million t [114]. This estimate was given for their entire coastal range (~154.715 km^2^), but it appeared difficult to achieve an accurate biomass estimate solely for our model area. We thus assumed that the distribution of Pacific hake was relatively even throughout their range, and scaled this biomass to our model area only, yielding an overall estimate of ~ 4.02 t/km^2^. The total estimated exploitation rate for this species was about 8.2% in 1979, which produced a fishing mortality of 0.082 year^-1^. The consumption ratio (Q/B) of this functional group was extracted from FishBase and set at 1.85 year^-1^ [68]. The natural mortality rate was estimated at 0.22 year^-1^, yielding a production biomass ratio of 0.302 year^-1^[68].

Pacific sand lance (*Ammodytes hexapterus*) is another ecologically important species found along the northeast Pacific coast, and was included as a functional group as they are found in the diet of harbor seals and most adult Pacific salmon species [33, 115]. There are no estimates of population trends for this forage species, as individuals often escape surveys given that they spend the majority of their time buried into sandy areas when not feeding [116]. The initial biomass for the year 1979 was set at 1.5 t/km^2^, and coupled with the pedigree *guesstimate* [117]. The P/B ratio was based on the natural mortality provided in FishBase and set at 0.77 year^-1^, while Q/B was estimated at 7.3 year^-1^ [68].

The other less important fish species that were found in the diet of the focal species of our model (i.e. marine mammals, Pacific salmon species) were aggregated into two other functional groups referred to as ‘Other forage fish’ and Other fish’, both of which were assumed to contain hundreds of different species. The Ecopath parameters for ‘Other forage fish’ were extracted from FishBase and averaged based on life-history estimates for Pacific sardines (*Scardinops sagax*) and northern anchovies (*Engraulis mordax*), yielding a Q/B of 38.4 year^-1^ and a P/B or 0.77 year^-1^ [68]. It was deemed reasonable to use those two species as proxies for this entire functional, as most species included in this group would be small pelagic fish species found at a similar trophic level. As it is impossible to accurately estimate the biomass of this functional group in 1979, we used a reasonable biomass estimate of 10 t/km^2^ and assigned a low pedigree (i.e. poor-quality data) to this number [62]. The biomass of the highly aggregated ‘Other fish’ functional group was set at 40 t/km^2^, and also assigned a low pedigree. The P/B of this group was set at 0.5 year^-1^ while the Q/B was 2.5 year^-1^. This would mean that the production would represent about 20% of the consumption (i.e. P/Q=0.2), which was deemed reasonable given that this group includes small fast-growing fish as well as larger species [62].

The ‘Invertebrates’ functional group was used to regroup the species that are preyed upon by most functional groups, and included shrimp, crabs, squids, and euphausiids (i.e. krill). As it was impossible to isolate invertebrate species based on diet studies, all biomasses (i.e. shrimps: 5.650 t/km^2^, crabs: 3.8 t/km^2^, squids: 0.5 t/km^2^, krill: 18 t/km^2^) were added from Preikshot (2007) from his northeast Pacific model, yielding an overall biomass of 27.95 t/km^2^ for this group [28]. The estimate of krill biomass provided by Preikshot (2007) was originally derived from two assessments reported by Beamish et al. (2001) [118] for the Strait of Georgia (i.e. 80 t/km^2^) and by Mackas (1992) [119] for the entire coast of British Columbia (i.e. 4.5 t/km^2^). Several studies [120, 121] have provided P/B estimates for krill in the north Pacific Ocean, all ranging from 5.5 to 8 year^-1^. We averaged all those estimates and the P/B for krill was set at 6.5 year^-1^. Following Preikshot’s method, the P/B ratios of shrimps, crabs, squids were set at 1.2 year^-1^, 1.5 year^-1^, and 3 year^-1^, respectively [28]. The overall P/B for this functional group (i.e. 3.05 year^-1^) was the non-weighted average of all listed estimates. All consumption ratios (Q/B) were extracted from Preikshot (2007) and averaged at 11.1 year^-1^.

The biomass, Q/B, and P/B estimates for other zooplankton (carnivorous and herbivorous zooplankton) were also extracted from Preikshot (2007), and set at 50 t/km^2^, 50 year^-1^, and 17 year^-1^, respectively [28]. The biomass was originally calculated based on estimates provided by Aydin et al. (2003) [91] for the eastern subarctic region, while the Q/B and P/B estimates were based on values provided by Beamish et al. (2001) [118] and Pauly et al. (1996) [122] for Strait of Georgia and the northeast Pacific region. The biomass for the phytoplankton functional group included both the macrophyte and phytoplankton estimates provided by Preikshot (2007) for the continental shelf of British Columbia, and was set at 31 t/km^2^. Those estimates were calculated based on different studies providing primary consumer biomasses for the Strait of Georgia [118], the Juan de Fuca Strait [123], and the west coast of Vancouver Island [124]. The P/B estimate provided by Beamish et al (2001) for the Strait of Georgia between 1998 and 2001 was used as a proxy for our model area, and set at 130 year^-1^[118]. Finally, the ‘detritus’ is the only functional group present by default in Ecopath. Its biomass was extracted from Preikshot (2007) and set at 10 t/km^2^ [28].

**SECTION 2: DIET COMPOSITION**

Following Ecopath diet composition setting guidelines, each prey contributions to predatory diets were converted into weight proportions [62]. All the relative diet composition estimates of the 74 functional groups are listed in Table A2 (marine mammals), A3 (Chinook salmon), and A4 (all other functional groups).

**Table A2.** **Diet composition of the marine mammal groups included in the northeast Pacific model.** Predators are listed in columns and prey species in the rows.

| **Predators (columns)** | TKW | SRKW | NRKW | Porpoises/dolphins | Harbor seals | Steller sea lions | California sea lions | Seabirds |
| --- | --- | --- | --- | --- | --- | --- | --- | --- |
| **Prey (rows)** |  |  |  |  |  |  |  |  |
| Porpoises/dolphins | 0.2920 | 0 | 0 | 0 | 0 | 0 | 0 | 0 |
| Harbor seals | 0.2469 | 0 | 0 | 0 | 0 | 0 | 0 | 0 |
| Steller sea lions | 0.2165 | 0 | 0 | 0 | 0 | 0 | 0 | 0 |
| California sea lions | 0.1082 | 0 | 0 | 0 | 0 | 0 | 0 | 0 |
| FRGSPS SP *Smolts* | 0 | 0 | 0 | 0 | 0 | 0 | 0 | 0.0010 |
| FRGSPS SP *Returning spawners* | 0 | 0.0168 | 0.0118 | 0 | 0.0006 | 0.0018 | 0.0005 | 0 |
| FRGSPS SU *Smolts* | 0 | 0 | 0 | 0 | 0.0004 | 0 | 0 | 0.0005 |
| FRGSPS SU *Juveniles* | 0 | 0 | 0 | 0 | 0.0002 | 0.0003 | 0.0002 | 0 |
| FRGSPS SU R*eturning spawners* | 0 | 0.0637 | 0.0186 | 0 | 0.0006 | 0.0010 | 0.0010 | 0 |
| FRGSPS FA *Smolts* | 0 | 0 | 0 | 0 | 0.0146 | 0 | 0 | 0.0005 |
| FRGSPS FA *Juveniles* | 0 | 0 | 0 | 0 | 0.0102 | 0.0028 | 0.0006 | 0 |
| FRGSPS FA *Marine* | 0 | 0 | 0 | 0 | 0.0091 | 0 | 0 | 0 |
| FRGSPS FA *Returning spawners* | 0 | 0.1108 | 0.1403 | 0 | 0.0085 | 0.0149 | 0.0144 | 0 |
| WCVI FA *Smolts* | 0 | 0 | 0 | 0 | 0 | 0 | 0 | 0.0005 |
| WCVI FA *Juveniles* | 0 | 0 | 0 | 0 | 0.0004 | 0.0028 | 0.0006 | 0 |
| WCVI FA *Returning spawners* | 0 | 0.1108 | 0.0873 | 0 | 0.0006 | 0.0031 | 0.0031 | 0 |
| CRWORC SP *Smolts* | 0 | 0 | 0 | 0 | 0 | 0 | 0 | 0.0005 |
| CRWORC SP *Juveniles* | 0 | 0 | 0 | 0 | 0.0004 | 0 | 0.0003 | 0 |
| CRWORC SP *Returning spawners* | 0 | 0.1108 | 0.0192 | 0 | 0.0006 | 0.0031 | 0.0031 | 0 |
| CRWORC SU *Smolts* | 0 | 0 | 0 | 0 | 0 | 0 | 0 | 0.0005 |
| CRWORC SU *Juveniles* | 0 | 0 | 0 | 0 | 0.0004 | 0 | 0.0006 | 0 |
| CRWORC SU *Returning spawners* | 0 | 0.0488 | 0.0187 | 0 | 0.0006 | 0.0031 | 0.0031 | 0 |
| CRWORC FA *Smolts* | 0 | 0 | 0 | 0 | 0 | 0 | 0 | 0.0005 |
| CRWORC FA *Juveniles* | 0 | 0 | 0 | 0 | 0.0004 | 0 | 0.0006 | 0 |
| CRWORC FA *Returning spawners* | 0 | 0.1108 | 0.0873 | 0 | 0.0006 | 0.0031 | 0.0031 | 0 |
| Coho *Smolts* | 0 | 0 | 0 | 0 | 0.0385 | 0 | 0 | 0.0005 |
| Coho *Marine* | 0 | 0 | 0 | 0 | 0.0857 | 0.0079 | 0.0077 | 0 |
| Coho *Returning spawners* | 0 | 0.0376 | 0.0459 | 0.0207 | 0.0102 | 0.0329 | 0.0486 | 0 |
| Chum *Smolts* | 0 | 0 | 0 | 0 | 0.0089 | 0 | 0 | 0.0005 |
| Chum *Returning spawners* | 0 | 0.1423 | 0.1122 | 0 | 0.0444 | 0.0268 | 0.0408 | 0 |
| Other salmonids | 0 | 0.0102 | 0.0121 | 0.2184 | 0.0418 | 0.0345 | 0.0603 | 0 |
| Herring *Adults* | 0 | 0 | 0 | 0.5786 | 0.1956 | 0.3237 | 0.3082 | 0.0983 |
| Halibut | 0 | 0.0102 | 0 | 0 | 0 | 0 | 0 | 0 |
| Hake | 0 | 0 | 0 | 0.1419 | 0.2134 | 0.1726 | 0.1407 | 0.1974 |
| Rockfish | 0 | 0 | 0 | 0 | 0.0045 | 0.0604 | 0.0343 | 0.1091 |
| **Table A2. *Continued***  Lingcod | 0 | 0.0610 | 0.0481 | 0 | 0.0037 | 0 | 0 | 0 |
| Pacific sand lance | 0 | 0 | 0 | 0 | 0.0059 | 0 | 0 | 0 |
| Other forage fish | 0 | 0 | 0 | 0 | 0.1497 | 0.1526 | 0.1641 | 0.1697 |
| Other fish | 0 | 0.1667 | 0.3984 | 0.0404 | 0.1497 | 0.1526 | 0.1641 | 0.4150 |
| Invertebrates | 0 | 0 | 0 | 0 | 0 | 0 | 0 | 0.0054 |
| Import | 0.1364 | 0 | 0 | 0 | 0 | 0 | 0 | 0 |

**Table A3. Diet composition of the Chinook salmon functional groups included in the northeast Pacific model.** Predators are listed in the top row and prey species in the first column.

| **Predator** | FRGSPS SP *River* | FRGSPS SP *Smolts* | FRGSPS SP *Juveniles* | FRGSPS SP *Marine* | FRGSPS SP *Returning spawners* | FRGSPS SP *Escapees* | FRGSPS SU *River* | FRGSPS SU *Smolts* | FRGSPS SU *Juveniles* | FRGSPS SU *Marine* | FRGSPS SU *Returning spawners* | FRGSPS SU *Escapees* |
| --- | --- | --- | --- | --- | --- | --- | --- | --- | --- | --- | --- | --- |
| **Prey** |  |  |  |  |  |  |  |  |  |  |  |  |
| Herring *Juveniles* | 0 | 0.3410 | 0.3410 | 0 | 0 | 0 | 0 | 0.3410 | 0.3410 | 0 | 0 | 0 |
| Herring *Adults* | 0 | 0 | 0 | 0 | 0.3000 | 0 | 0 | 0 | 0 | 0 | 0.3000 | 0 |
| Pacific sand lance | 0 | 0 | 0 | 0 | 0.3000 | 0 | 0 | 0 | 0 | 0 | 0.3000 | 0 |
| Other forage fish | 0 | 0 | 0 | 0.5000 | 0 | 0.5000 | 0 | 0 | 0 | 0.5000 | 0 | 0.5000 |
| Other fish | 0 | 0.3540 | 0.3540 | 0.5000 | 0 | 0.5000 | 0 | 0.3540 | 0.3540 | 0.5000 | 0 | 0.5000 |
| Invertebrates | 0 | 0.1525 | 0.1525 | 0 | 0.4000 | 0 | 0 | 0.1525 | 0.1525 | 0 | 0.4000 | 0 |
| Zooplankton | 1.0000 | 0.1525 | 0.1525 | 0 | 0 | 0 | 1.0000 | 0.1525 | 0.1525 | 0 | 0 | 0 |
| Phytoplankton | 0 | 0 | 0 | 0 | 0 | 0 | 0 | 0 | 0 | 0 | 0 | 0 |
| Import | 0 | 0 | 0 | 0 | 0 | 0 | 0 | 0 | 0 | 0 | 0 | 0 |

| **Predator** | FRGSPS FA *River* | FRGSPS FA *Smolts* | FRGSPS FA *Juveniles* | FRGSPS FA *Marine* | FRGSPS FA *Returning spawners* | FRGSPS FA *Escapees* | WCVI FA *River* | WCVI FA *Smolts* | WCVI FA *Juveniles* | WCVI FA *Marine* | WCVI FA *Returning spawners* | WCVI FA *Escapees* |
| --- | --- | --- | --- | --- | --- | --- | --- | --- | --- | --- | --- | --- |
| **Prey** |  |  |  |  |  |  |  |  |  |  |  |  |
| Herring *Juveniles* | 0 | 0.3410 | 0.3410 | 0 | 0 | 0 | 0 | 0.3410 | 0.3410 | 0 | 0 | 0 |
| Herring *Adults* | 0 | 0 | 0 | 0 | 0.3000 | 0 | 0 | 0 | 0 | 0 | 0.3000 | 0 |
| Pacific sand lance | 0 | 0 | 0 | 0 | 0.3000 | 0 | 0 | 0 | 0 | 0 | 0.3000 | 0 |
| Other forage fish | 0 | 0 | 0 | 0.5000 | 0 | 0.5000 | 0 | 0 | 0 | 0.5000 | 0 | 0.5000 |
| Other fish | 0 | 0.3540 | 0.3540 | 0.5000 | 0 | 0.5000 | 0 | 0.3540 | 0.3540 | 0.5000 | 0 | 0.5000 |
| Invertebrates | 0 | 0.1525 | 0.1525 | 0 | 0.4000 | 0 | 0 | 0.1525 | 0.1525 | 0 | 0.4000 | 0 |
| Zooplankton | 1.0000 | 0.1525 | 0.1525 | 0 | 0 | 0 | 1.0000 | 0.1525 | 0.1525 | 0 | 0 | 0 |
| Phytoplankton | 0 | 0 | 0 | 0 | 0 | 0 | 0 | 0 | 0 | 0 | 0 | 0 |
| Import | 0 | 0 | 0 | 0 | 0 | 0 | 0 | 0 | 0 | 0 | 0 | 0 |

**Table A3. Continued**

| **Predator** | CRWORC SP *River* | CRWORC SP *Smolts* | CRWORC SP *Juveniles* | CRWORC SP *Marine* | CRWORC SP *Returning spawners* | CRWORC SP *Escapees* | CRWORC SU *River* | CRWORC SU *Smolts* | CRWORC SU *Juveniles* | CRWORC SU *Marine* | CRWORC SU *Returning spawners* | CRWORC SU *Escapees* |
| --- | --- | --- | --- | --- | --- | --- | --- | --- | --- | --- | --- | --- |
| **Prey** |  |  |  |  |  |  |  |  |  |  |  |  |
| Herring *Juveniles* | 0 | 0.3410 | 0.3410 | 0 | 0 | 0 | 0 | 0.3410 | 0.3410 | 0 | 0 | 0 |
| Herring *Adults* | 0 | 0 | 0 | 0 | 0.3000 | 0 | 0 | 0 | 0 | 0 | 0.3000 | 0 |
| Pacific sand lance | 0 | 0 | 0 | 0 | 0.3000 | 0 | 0 | 0 | 0 | 0 | 0.3000 | 0 |
| Other forage fish | 0 | 0 | 0 | 0.5000 | 0 | 0.5000 | 0 | 0 | 0 | 0.5000 | 0 | 0.5000 |
| Other fish | 0 | 0.3540 | 0.3540 | 0.5000 | 0 | 0.5000 | 0 | 0.3540 | 0.3540 | 0.5000 | 0 | 0.5000 |
| Invertebrates | 0 | 0.1525 | 0.1525 | 0 | 0.4000 | 0 | 0 | 0.1525 | 0.1525 | 0 | 0.4000 | 0 |
| Zooplankton | 1.0000 | 0.1525 | 0.1525 | 0 | 0 | 0 | 1.0000 | 0.1525 | 0.1525 | 0 | 0 | 0 |
| Phytoplankton | 0 | 0 | 0 | 0 | 0 | 0 | 0 | 0 | 0 | 0 | 0 | 0 |
| Import | 0 | 0 | 0 | 0 | 0 | 0 | 0 | 0 | 0 | 0 | 0 | 0 |

| **Predator** | CRWORC FA *River* | CRWORC FA *Smolts* | CRWORC FA *Juveniles* | CRWORC FA *Marine* | CRWORC FA *Returning spawners* | CRWORC FA *Escapees* |
| --- | --- | --- | --- | --- | --- | --- |
| **Prey** |  |  |  |  |  |  |
| Herring *Juveniles* | 0 | 0.3410 | 0.3410 | 0 | 0 | 0 |
| Herring *Adults* | 0 | 0 | 0 | 0 | 0.3000 | 0 |
| Pacific sand lance | 0 | 0 | 0 | 0 | 0.3000 | 0 |
| Other forage fish | 0 | 0 | 0 | 0.5000 | 0 | 0.5000 |
| Other fish | 0 | 0.3540 | 0.3540 | 0.5000 | 0 | 0.5000 |
| Invertebrates | 0 | 0.1525 | 0.1525 | 0 | 0.4000 | 0 |
| Zooplankton | 1.0000 | 0.1525 | 0.1525 | 0 | 0 | 0 |
| Phytoplankton | 0 | 0 | 0 | 0 | 0 | 0 |
| Import | 0 | 0 | 0 | 0 | 0 | 0 |

**Table A4.** **Diet composition of the last functional groups included in the northeast Pacific model.** Predators are listed in the top row and prey species in the first column.

| **Predator** | Coho *River* | Coho *Smolts* | Coho *Marine* | Coho *Returning spawners* | Coho *Escapees* | Chum *River* | Chum *Smolts* | Chum *Marine* | Chum *Returning spawners* | Chum *Escapees* |
| --- | --- | --- | --- | --- | --- | --- | --- | --- | --- | --- |
| **Prey** |  |  |  |  |  |  |  |  |  |  |
| Herring *Juveniles* | 0 | 0.0538 | 0 | 0 | 0 | 0 | 0 | 0 | 0 | 0 |
| Herring *Adults* | 0 | 0 | 0 | 0.2500 | 0 | 0 | 0 | 0 | 0 | 0 |
| Halibut | 0 | 0 | 0 | 0 | 0 | 0 | 0 | 0 | 0 | 0 |
| Hake | 0 | 0 | 0 | 0 | 0 | 0 | 0 | 0 | 0 | 0 |
| Rockfish | 0 | 0 | 0 | 0 | 0 | 0 | 0 | 0 | 0 | 0 |
| Lingcod | 0 | 0 | 0 | 0 | 0 | 0 | 0 | 0 | 0 | 0 |
| Pacific sand lance | 0 | 0 | 0 | 0.2500 | 0 | 0 | 0 | 0 | 0 | 0 |
| Other forage fish | 0 | 0 | 0.5000 | 0 | 0.5000 | 0 | 0 | 0.5000 | 0.1100 | 0.5000 |
| Other fish | 0 | 0.9462 | 0.5000 | 0 | 0.5000 | 0 | 0 | 0.5000 | 0.1100 | 0.5000 |
| Invertebrates | 0 | 0 | 0 | 0.5000 | 0 | 0 | 0.5000 | 0 | 0.7800 | 0 |
| Zooplankton | 1.0000 | 0 | 0 | 0 | 0 | 1.0000 | 0.5000 | 0 | 0 | 0 |
| Phytoplankton | 0 | 0 | 0 | 0 | 0 | 0 | 0 | 0 | 0 | 0 |
| Detritus | 0 | 0 | 0 | 0 | 0 | 0 | 0 | 0 | 0 | 0 |
| Import | 0 | 0 | 0 | 0 | 0 | 0 | 0 | 0 | 0 | 0 |

**Table A4. Continued**

| **Predator** | Other salmonids | Herring *Juveniles* | Herring *Adults* | Halibut | Hake | Rockfish | Lingcod | Pacific sand lance | Other forage fish | Other fish | Invertebrates | Zooplankton |
| --- | --- | --- | --- | --- | --- | --- | --- | --- | --- | --- | --- | --- |
| **Prey** |  |  |  |  |  |  |  |  |  |  |  |  |
| Herring *Juveniles* | 0 | 0 | 0 | 0 | 0 | 0 | 0.1155 | 0 | 0 | 0 | 0 | 0 |
| Herring *Adults* | 0.0050 | 0 | 0 | 0.1000 | 0.1025 | 0.0745 | 0.1155 | 0 | 0 | 0 | 0 | 0 |
| Halibut | 0 | 0 | 0 | 0 | 0 | 0 | 0 | 0 | 0 | 0 | 0 | 0 |
| Hake | 0 | 0 | 0 | 0 | 0 | 0 | 0.1212 | 0 | 0 | 0 | 0 | 0 |
| Rockfish | 0 | 0 | 0 | 0.1000 | 0 | 0 | 0.0110 | 0 | 0 | 0 | 0 | 0 |
| Lingcod | 0 | 0 | 0 | 0 | 0 | 0 | 0 | 0 | 0 | 0 | 0 | 0 |
| Pacific sand lance | 0.1000 | 0 | 0 | 0.1000 | 0 | 0.0745 | 0.3030 | 0 | 0 | 0 | 0 | 0 |
| Other forage fish | 0 | 0 | 0.1000 | 0.2500 | 0.3365 | 0.1064 | 0.0606 | 0 | 0 | 0 | 0 | 0 |
| Other fish | 0 | 0 | 0.1000 | 0.2500 | 0.3365 | 0.1064 | 0.0909 | 0 | 0 | 0 | 0 | 0 |
| Invertebrates | 0.7950 | 0 | 0.1000 | 0.2000 | 0.2244 | 0.6383 | 0.1818 | 0.5000 | 0.2500 | 0.2500 | 0 | 0 |
| Zooplankton | 0.1000 | 1.0000 | 0.7000 | 0 | 0 | 0 | 0 | 0.5000 | 0.5000 | 0.5000 | 0.5000 | 0 |
| Phytoplankton | 0 | 0 | 0 | 0 | 0 | 0 | 0 | 0 | 0.2500 | 0.2500 | 0.5000 | 1.0000 |
| Detritus | 0 | 0 | 0 | 0 | 0 | 0 | 0 | 0 | 0 | 0 | 0 | 0 |
| Import | 0 | 0 | 0 | 0 | 0 | 0 | 0 | 0 | 0 | 0 | 0 | 0 |

##### Marine mammals

In contrast to fish-eating *resident* killer whales, *transient* killer whales feed upon other marine mammals. Previous diet studies in British Columbia estimated that harbor seals (*phoca vitulina*) and Steller sea lions (*Eumetopias jubatus*) represented 52% and 13% of the predation events recorded, respectively [125]. Other smaller marine mammals, including harbor porpoises (*Phocoena phocoena*), Dall's porpoises (*Phocoenoides dalli*), and Pacific white-sided dolphins (*Lagenorhynchus obliquidens*) represented the last 35% of the TKW diet. In California, TKW are also known to feed on California sea lions, which can represent up to 35% of their diet in some restricted specific areas [126]. The relative TKW diet proportions (in prey biomass) for the different marine mammals was adjusted, assuming that the average weight of the main preys were 44.7 kg (harbor seal), 52.5 kg (harbor porpoise), 136 kg (Dall’s porpoise), 280 kg (Steller sea lion), and 240 kg (California sea lion).

The diet of SRKW was estimated following recent diet studies during which field sampling was done both in the winter and in the summer months [107, 108, 127]. For each prey type, the relative seasonal prey proportions computed by Hanson et al. (2010, 2021) were averaged over the year [16, 107]. Although SRKW almost exclusively feed on Chinook salmon from May to July, they were recently found to exhibit a more diverse diet during the winter months, feeding on other fish such as lingcod, flatfish (e.g., English sole (*Parophrys vetulus*), big skate (*Rana binoculata*)), Pacific halibut, and steelhead salmon (*Oncorhynchus mykiss*). The yearly averaged prey contribution for Chinook salmon was estimated at 69%, while lingcod, chum salmon, and coho salmon represented 5%, 20%, and 6%, respectively [108]. Based on an average 4-year-old Chinook salmon weight of 8.5 kg, this species was found to constitute about 76% of the SRKW diet in prey biomass [14, 128]. This percentage was originally divided between all seven regional Chinook salmon groups, and all assigned to the *Returning spawners* stanzas, as predation on young fish is rare and mainly happens during a limited period of the year [14, 108]. Ford and Ellis (2006) showed that most of the SRKW predation occurs on Chinook salmon older than 2-year-old, and suggest that the catch of juvenile salmon is usually associated with playing and training in young calves [14]. No detailed diet study was found regarding the NRKW population. It is known that NRKW are also dietary specialists, feeding primarily on fish species, especially Chinook salmon [129]. In this context, the diet of both *resident* killer whale populations was assumed to be similar. However, a larger proportion of the diet of NRKW was allocated to ‘Other fish’, as they are known to feed on salmon stocks that are not included in our model area, especially during the winter months [130].

With the harbor seal population growing all over the Pacific coast since their protection in 1972, there has recently been a considerable scientific interest regarding their diet composition. Harbor seals are considered opportunistic predators that are able to feed on different species of fish depending on their local and seasonal abundance [95]. Most studies looking at harbor seal diets use the frequency of occurrence (FO) method, which indicates the frequency (in percentage) at which samples contain any particular prey type [94, 95]. Except if adjusted, FO do not add up to 100% and are not representative of the diet fraction of each prey. For this reason, we based the harbor seals diet estimations on two diet studies [131, 132] that used DNA sequencing and metabarcoding. Based on those studies, Pacific herring and Pacific hake represented about 22% and 24% of the HS diet, respectively. Chinook salmon could represent about 3.5% of the harbor seal diet, 85% of which are thought to be smolts or juveniles. In order to divide the predation mortality of harbor seals between the *Smolts* and *Juveniles* stanzas of the different Chinook salmon regional groups, it was important to take into consideration the life-history of each of those groups. Stream-type (mainly yearling salmon) Chinook salmon are known to undertake a rapid migration after entering the marine environment, and are thought to rear mainly in offshore waters where they spend their first ocean year [65]. In contrast, ocean-type (mainly sub-yearling salmon) are found in more coastal sheltered areas and are thus likely more prone to predation [65] (. Based on those facts, harbor seals were assumed to prey on Chinook salmon *Smolts* and *Juveniles* stanzas originating from the summer and fall runs of the FRGSPS functional groups. Further, fewer harbor seals are known to inhabit the southern coastal waters of our model, and they were thus assumed to prey only on out-migrating juvenile Chinook salmon of the CRWORC functional groups [36, 133]. Adult chum salmon is also a significant species consumed by harbor seals (~ 6%), especially in the late fall [132]. Chum salmon enter the ocean as fry during their first summer before rapidly migrating northwards and eastwards, hence only about 1% of the harbor seal consumption was assigned to the *Smolts* stanza [131, 134]. In addition, there are no known resident chum salmon populations in the Salish Sea, as this area is mainly used by this species as a migratory corridor [82]. For this reason, no harbor seal predation was assigned to the *Marine* stanza of this group.

Both California and Steller sea lion are important predators of Pacific hake and herring. Although slight diet differences exist between female and male Steller sea lion, they are both known to consume a high proportion of forage fish (~13% on average), rockfish (~19% on average), and salmon (~15.5% on average) (Trites and Calkins 2008). Chasco et al. (2017) estimated that juvenile and adult Chinook salmon represented about 2.0% and 4.5% of SSL diet (~ 0.005 % and 2.4 % in prey biomass, respectively) [135]. Only sub-adult and adult California sea lion males are seen in the Salish Sea during the winter months, and there is a paucity of information regarding potential seasonal and geographical changes in diet for this species. In the Gulf of California, rockfish and hake were found to be a considerable percentage of the prey biomass consumed by California sea lion, representing 28.6% and 2.1% of their diet, respectively (Weise and Harvey 2008). Chasco et al. (2017) estimated that juvenile and adult Chinook salmon represented about 1% and 5.3% of California sea lions’ diet (0.02 % and 23.8% in prey biomass, respectively) [135]. Other preys of California sea lions include juvenile spiny dogfish (*Squalus acanthias*), Pacific jack mackerel (*Trachurus symmetricus*), anchovy (Engraulis mordax), market squid (*Doryteuthis opalescens*), as well as other salmonids. To our knowledge, there has been no publication looking at the diet of California sea lion in Canadian waters, so the diet presented by Weise and Harvey (2008) was taken as a proxy for our entire study area [48].

Both common murres and rhinoceros auklets are known as piscivorous bird species [51, 136, 137]. Finding information on the diet by weight of those species was challenging, as most studies use the frequency of occurrence method. We assumed that the diet of both species was comparable, as they both primarily feed on herring and other small fish species such as juvenile salmonids and gadids [136, 138]. As no diet by weight estimates was found for rhinoceros auklets, we used the diet composition data reported for common murres by Ainley et al. (1996) in California waters during the breeding and wintering periods as a proxy for this functional group [137].

Finally, no accurate diet studies were found for the Pacific white-sided dolphin, Dall’s porpoise, or harbor porpoise. Heise (1996) suggested that herring, Pacific salmons (i.e. pink, sockeye, and coho), and gadids could represent about 59%, 30%, and 6% of the diet of Pacific white-sided dolphins, respectively [26]. Dall’s and harbor porpoises’ diet could be constituted of about 50% herring and 20% hake, but those estimates likely vary depending on what resources are available to the whales [139, 140]. We used a combination of those estimates along with the average weights of the different prey items to calculate the relative proportion of the different species into the diet of the ‘Dolphins and porpoises’ functional group.

##### Salmon species

Accurate information on the diet of fry Pacific salmon could not be found, but it was deemed reasonable to assume that fish individuals of the *River* stanza would only feed on small freshwater invertebrates. In addition, it was assumed that the majority of fish individuals included in the *Marine* and *Escapees* stanzas of the different Pacific salmon functional groups would feed outside of our model area, and 100% of their diet was thus allocated to ‘Other fish’ and ‘Other forage fish’. It is known that smolt and juvenile Chinook salmon primarily feed on small crustaceans, but information on a potential age-specific diet remain scarce [141]. We thus assumed that the diet was similar for both stanzas as it is unclear when the diet transitions occur. We used the diet estimates provided by Beamish et al (2012) for juvenile Chinook salmon, and assumed that juvenile herring and ‘Other fish’ were representing about 34.1 % and 35.4% of the volume of their diet, respectively [99]. This study was carried out between 1998 and 2009 (omitting 2007) in the Strait of Georgia. Zooplankton and invertebrates, such as euphausiids, squids, and amphipods, constituted the remainder of their diet [115, 142]. Diets of adult Chinook salmon were extracted from information provided in Beacham (1986), and included sand lance (~30%), herring (~30 %), and different species of invertebrates (~40%) [143]. Although there are seasonal and regional differences in the diet of adult Chinook salmon, there was not enough information available to use distinct diet estimates for our different Chinook salmon functional groups [115]. The diet of Chinook and coho salmon are quite similar, although the overall proportion of fish prey is higher for Chinook salmon [143]. Coho smolt and juvenile are known to mainly feed on zooplankton, juvenile herring, invertebrates, and other small forage fish[144], while adult coho salmon feed on sand lance (~25%), herring (~25%), and different species of invertebrates [99, 143]. Recent surveys carried out in British Columbia revealed that juvenile chum salmon primarily feed on zooplankton and small invertebrates, especially planktonic gelatinous prey [144]. Information on the diet of adult chum salmon is hard to find for our model area, but several studies [145–147] examined the stomach contents of this species during its oceanic phase in the Bering Sea. In this region, it appears that chum salmon primarily feed on small crustaceans, gelatinous zooplankton, and small fish species such as Walleye pollock (*Gadus chalcogrammus*) and mackerel (*Scomber* spp.). According to Beacham (1986), fish could only represent 10% to 35% of the diet of adult chum salmon [143]. Based on those findings, 22% of the diet of the *Returning spawners* stanza of chum salmon was evenly allocated between ‘Other fish’ and ‘Other forage fish’, while 78% was allocated to ‘Invertebrates’. Finally, diet estimates for pink and sockeye salmon (i.e. ‘Other salmonids’ functional group) were extracted from Beacham (1986) and allocated to sand lance (~10%), zooplankton (~10%), adult herring (0.5%), and invertebrates (~79.5%) [143].

##### Other species

Juvenile herring are known to feed on small zooplankton, while adults primarily feed on small fish (~20%), zooplankton (~70%), and planktonic crustaceans and other small invertebrates (~10%) [148]. Pacific halibut primarily feed on planktonic organisms, invertebrates, and small fish during their first years of life [110, 149]. Different species of fish, such as herring, sand lance, rockfish, and capelin (*Mallotus villosus*) start to make up a larger portion of halibut’s diet as they grow. Adult halibut are also known to consume benthic invertebrates such as crabs and molluscs [110, 149]. Based on that information, the relative diet proportions of this functional group were allocated to ‘Other fish’/’Other forage fish’ (~50%), herring (~10%), sand lance (~10%), rockfish (~10%), and invertebrates (~20%). Given the wide distribution of Pacific hake, it is likely that the diet of this species varies throughout its range. Overall, Pacific hake are known to feed on small schooling pelagic fish and crustaceans, such as euphausiids and herring [97]. Here, we used averages of diet proportion estimates of Pacific hake for the Vancouver area (i.e. west coast of Vancouver Island, Salish Sea) and the Columbia River as a proxy for the entire functional group. Pacific hake were assumed to feed on ‘Other fish’/’Other forage fish’ (~40%), adult herring (~40%), and invertebrates (~20%) [97]. It is believed that the diet of rockfish varies between species and life stages [150]. However, studies [150, 151] agree that rockfish primarily feed on invertebrates, Pacific herring, Pacific sand lance, and other smaller fish such as surfperches (*Embiotocidae* spp.) and greenlings (*Hexagrammidae* spp.). Lingcods are known to be ravenous predators feeding primarily on small fish such as sand lance, rockfish, and Pacific herring and hake [152, 153]. For instance, Beaudreau and Essington (2007) showed that rockfish could represent 11% (by weight) of the diet of lingcod in the San Juan Archipelago [152]. Information on the diet of sand lance were extracted from a study conducted by Hipfner and Galbraith (2014) in the Salish Sea in the 1960s [154]. According to this study, the diet of sand lance is dominated by copepods and crustaceans. Given the highly aggregated nature of the ‘Other fish’ and ‘Other forage fish’ functional groups, it was difficult to quantify their diet with accuracy as diet surely differs between species. In this context, it was deemed reasonable to assume that most of the small species included in those group would primarily feed on planktonic organisms and small invertebrates. Finally, 100% of the diet of invertebrates was allocated to zooplankton and phytoplankton while zooplankton organisms were assumed to solely feed on phytoplankton.

#### Other parameters

Other Ecopath basic input parameters include habitat area (i.e. proportion of the habitat occupied by a functional group) and unassimilated consumption (i.e. fraction of the food ingested that is not assimilated) [62]. The initial biomass of each functional group was adjusted depending on the geographical range of each species, so the habitat area default value of 1 was used. On the other hand, the unassimilated consumption default value of 0.2 was used for all of our carnivorous functional groups, as suggested by Winberg (1962) [62, 155]. This number suggests that about 80% of the food is assimilated by a species, while 20% is non-assimilated and directed to the detritus group. Following the Christensen et al. (2008) suggestion, this parameter was set at 0.4 for zooplankton, as herbivorous organisms usually show a higher proportion of non-assimilated food [62].

**SECTION 3: FUNCTIONAL GROUP ECOSIM PARAMETERS**

Ecosim simulations permit to fit predicted temporal changes in biomass and mortality to time-series of reference data. Except if stated otherwise, all biomass time-series were entered as relative to the base Ecopath estimates.

##### Killer whales

The number of individuals in the TKW population was extracted from Ford et al. (2007) [1]for each year between 1979 and 2006, and from Towers et al. (2019) [156] for each year between 2008 and 2019. The average annual growth rate of the TKW population was estimated at 2.7% from 2006 to 2011, and rose to about 4.1% after 2012 [125, 156]. This higher population growth in recent years is likely linked to high survival, greater immigration, and steady reproduction rates, and might be associated with the rising occurrence of TKW sightings in the coastal waters of British Columbia over those years [156, 157]. The population abundance of TKW between 2006 and 2019 was estimated using the Malthusian population growth model (Malthus 1798), according to which $N_{future}=N_{present}* {(1+r)}^{t}$, where *N* represents the population size, *r* the population growth rate, and *t* the time period [158]. TKW are known to exhibit large-scale movement patterns that are likely associated with the seasonal distribution of the marine mammal species they feed upon, and occur in offshore waters (i.e. outside of the model area) for some periods of the year [125]. As it would be challenging to accurately predict the population trend of a functional group that occurs outside the model area, the biomass time series of the TKW population was entered in Ecosim as a forced absolute biomass (i.e. Ecosim data type *-1*). When time series are forced in Ecosim, data need to be entered for every year of model. No precise TKW population assessment was found for the year 2020, so the annual growth rate estimates of this population after 2012 (i.e. 4.1%) was used to estimate the population abundance of this functional group for the last year of the model.

The births and deaths data collected by the Center for Whale Research were used to estimate the number of individuals as well as the age- and sex- structure of the SRKW population from 1979 to 2020 [11]. Following the method described earlier in this chapter, the biomass of the SRKW population was calculated for each year of the model, and entered as a relative biomass time series (i.e. Ecosim data type *0*). A time-series of instantaneous fishing mortality rates F (i.e. Ecosim data type *4*) was also read on Ecosim to account for non-natural deaths that have been documented since 1979 (e.g., blunt trauma, infections) [11].

The estimated number of individuals occurring in the NRKW population was extracted from Towers (2015) [159] for the period 1979 to 2014, and from Fisheries and Oceans Canada (2022) [160] for the years 2014-2020. The demographic structure of the NRKW population has been relatively stable over the last decades, yet data on the exact age- and sex- structure of this group could not be found [160]. In this context, the average adult individual weight estimate (i.e. 2998 kg) used in Ecopath was used to calculate the relative biomass of the entire population between 1979 and 2020.

##### Pinnipeds

To calculate the overall abundance of harbor seals, we summed the estimates provided for the Washington coast (including Puget Sound) [36], the Columbia River and Oregon Coast [37], as well as for the entire coast of British Columbia [35, 39]. The biomass estimates for this group were only entered for years were estimates for all those regions were available. This population increased at an annual rate of about 11.5% from the late 1970s to the late 1990s, with the population of harbor seals in the Strait of Georgia having increased about ten-fold during this period [39]. The growth of the population began to slow in the late 1990s, and the population appeared to stabilize and reach carrying capacity in the early 2000s [161].

Several studies [40, 42] have provided historical population estimates of the Eastern Steller sea lion stock throughout its range (i.e. southeast Alaska to California). For this model, we used the population estimates of Steller sea lion in British Columbia as a proxy for the entire model area. This was deemed reasonable, as the largest rockeries (i.e. breeding sites) are found in British Columbia and Alaska. In addition, it is likely that individuals from neighboring rockeries in Washington, Oregon, and California states move northwards to the coastal rockeries of British Columbia during the winter time [42]. We used the abundance estimates of pups and non-pup individuals provided by Fisheries and Oceans (2021) between 1979 and 2018 for the entire coast of British Columbia. It is believed that because commercial harvests and predator control kills were still occurring in British Columbia until 1968, this population did not show significant increase in growth rate in the early 1970s [40]. From the early 1990s to 2017, the population steadily increased at an annual rate of about 4.5% [42]. The production of pup appears to have been slowing since then.

The overall population estimate of the United States California sea lion population was extracted from Laake et al. (2018) for the period 1975-2014 [44]. For the biomass time series, the same approach as the one used for our Ecopath base year estimate was used. The abundance estimate was divided by two, as females are considered non-migratory and remain close from the rockeries throughout the entire year [162]. In addition, our model area represented only about 45% of the total range of male California sea lions [44].

##### Salmon species

The method used to calculate the biomass of each Chinook salmon functional groups in Ecopath was applied to build all the time series imported into Ecosim. All fisheries catch and escapement data were provided by the Joint Chinook Technical Committee of the Pacific Salmon Commission, and used to provide biomass estimates from 1979 to 2020. Fishing mortality time series were entered for all Chinook salmon *Marine*, *Returning spawners*, and *Escapees* stanzas as forced functions (i.e. Ecosim data type *-1*). Time series of total mortality of Chinook salmon *Smolts* were also entered in Ecosim, and extracted from the Appendix E of the exploitation rate analysis report provided by the Pacific Salmon Commission [59]. Stock-specific time series of smolt-to-Age 2 or 3 survival rates were converted to total mortality rates and used as proxies for our different Chinook salmon *Smolt*s stanzas, including Robertson Creek (used for WCVI), Nicola River (used for FRGSPS SP), lower Shushwap Lake (used for FRGSPS SU), as well as Chilliwack and Harrison Rivers (used for FRGSPS FA).

Catch, escapement, and cohort abundance estimates of coho salmon in the Salish Sea (Fraser River, Strait of Georgia, Puget Sound) were extracted from assessments presented by the Joint Technical Coho Committee of the Pacific Salmon Commission for the years between 1986 and 2010. Fisheries catch for the years 1979 to 1986 were extracted from the stock status report prepared by Fisheries and Oceans (2002) [78]. As no catch data were available prior to 1981, we assumed a similar fishing mortality from 1979 to 1981. Abundance and fisheries catch estimates for the Columbia River and the Washington, Oregon, and Californian coasts were extracted from the pre-season report of the Pacific Fishery Management Council (2022) [60]. Fishing mortality time series were entered for coho salmon *Marine* and *Returning spawners* stanzas as forced functions. Time series of coho salmon *Smolts* (i.e. first ocean year) survival rates were calculated for the Strait of Georgia between 1979 and 2015, and used as proxy for this functional group throughout its range. Those estimates predicted the number of coho salmon alive before marine fisheries mortality occurs, and are based on calculations which include in-river mortality after hatchery releases as well as predation mortality. Smolt survival estimates provided by Zimmerman et al. (2015) for the period 1977- 2010 are similar than the ones used in this model, but extend over a shorter period of time. Finally, escapement and fisheries catch estimates for chum salmon in the interior Salish Sea, the WCVI, Puget Sound, and the Washington coast (Willapa Bay and Grays Harbor) were extracted from the Joint Chum Technical Committee reports produced by the Pacific Salmon Commission for different periods of time: 1979-1984 [85], 1985-1989 [163], 1994-1999 [164], 2000-2009 [165], and 2010-2018 [166]. Fishing mortality time series were entered for the *Returning spawners* stanza of this functional group (i.e. Ecosim data type *4*).

##### Other functional groups

Escapement data of pink and sockeye salmon for north America were taken as a proxy for our model area and were extracted directly from Ruggerone and Irvine (2018) [66]. Catch statistics were extracted from the online public database Salmonid Catch Statistics provided by the North Pacific Anadromous Fish Commission (NPAFC) for both sport and commercial fisheries in British Columbia, Alaska, and the Yukon territory for the period 1979-2020 [90]. Alaska and the Yukon territory were included in this search to match the geographical range of the escapement estimates provided by Ruggerone and Irvine (2018) [66]. The spawning biomass estimates for the *Adult*s herring stanza were extracted from Thompson et al. (2017), and the instantaneous fishing mortality time series were entered for the *Adults* herring stanza (i.e. Ecosim data type *4*).

**SECTION 4: VULNERABILITY SETTINGS**

**Table A5**. **List of the vulnerability parameters with the lowest sum of squared differences between model predictions and reference time-series data of biomass and mortalities for the eight marine mammal groups included in the model.** Predators are listed in the top row and prey species in the first column.

| **Predator** | TKW | SRKW | NRKW | Porpoises/dolphins | Harbor seals | Steller sea lions | California sea lions | Seabirds |
| --- | --- | --- | --- | --- | --- | --- | --- | --- |
| **Prey** |  |  |  |  |  |  |  |  |
| Porpoises/dolphins | 1.370 | _ | _ | _ | _ | _ | _ | _ |
| Harbor seals | 7 | _ | _ | _ | _ | _ | _ | _ |
| Steller sea lions | 1.200 | _ | _ | _ | _ | _ | _ | _ |
| California sea lions | 1.370 | _ | _ | _ | _ | _ | _ | _ |
| FRGSPS SP *Smolts* | _ | _ | _ | _ | _ | _ | _ | 1.200 |
| FRGSPS SP *Returning spawners* | _ | _ | _ | _ | 8.887 | 50 | 4.725 | 2 |
| FRGSPS SU *Smolts* | _ | _ | _ | _ | 8.887 | _ | _ | _ |
| FRGSPS SU *Juveniles* | _ | _ | _ | _ | 8.887 | 50 | 4.725 | _ |
| FRGSPS SU R*eturning spawners* | _ | 1.200 | 2.095 | _ | 8.887 | 50 | 4.725 | _ |
| FRGSPS FA *Smolts* | _ | _ | _ | _ | 15 | _ | _ | 1.200 |
| FRGSPS FA *Juveniles* | _ | _ | _ | _ | 1000 | 50 | 100 | _ |
| FRGSPS FA *Marine* | _ | _ | _ | _ | 1000 | _ | _ | _ |
| FRGSPS FA *Returning spawners* | _ | 1.200 | 20 | _ | 20 | 50 | 1000 | _ |
| WCVI FA *Smolts* | _ | _ | _ | _ | _ | _ | _ | 1.200 |
| WCVI FA *Juveniles* | _ | _ | _ | _ | 8.887 | 50 | 4.725 | _ |
| WCVI FA *Returning spawners* | _ | 1.200 | 2.095 | _ | 8.887 | 50 | 4.725 | _ |
| CRWORC SP *Smolts* | _ | _ | _ | _ | _ | _ | _ | 1.200 |
| CRWORC SP *Juveniles* | _ | _ | _ | _ | 8.887 | _ | 4.725 | _ |
| CRWORC SP *Returning spawners* | _ | 1.200 | 2.095 | _ | 8.887 | 50 | 4.725 | _ |
| CRWORC SU *Smolts* | _ | _ | _ | _ | _ | _ | _ | 1.200 |
| CRWORC SU *Juveniles* | _ | _ | _ | _ | 8.887 | _ | 4.725 | _ |
| CRWORC SU *Returning spawners* | _ | 1.200 | 2.095 | _ | 8.887 | 50 | 4.725 | _ |
| CRWORC FA *Smolts* | _ | _ | _ | _ | _ | _ | _ | 1.200 |
| CRWORC FA *Juveniles* | _ | _ | _ | _ | 8.887 | _ | 4.725 | _ |
| CRWORC FA *Returning spawners* | _ | 1.200 | 2.095 | _ | 8.887 | 50 | 4.725 | _ |
| Coho *Smolts* | _ | _ | _ | _ | ∞ | _ | _ | 1.200 |
| Coho *Marine* | _ | _ | _ | _ | ∞ | 50 | ∞ | _ |
| Coho *Returning spawners* | _ | 1.200 | 100 | 20 | 10 | 1000 | 1000 | _ |
| Chum *Smolts* | _ | _ | _ | _ | 8.887 | _ | _ | 1.200 |
| Chum *Returning spawners* | _ | 1.200 | 2.095 | _ | 8.887 | 50 | 4.725 | _ |
| Other salmonids | _ | 1.200 | 2.095 | 2 | 8.887 | 50 | 4.725 | _ |
| Herring *Adults* | _ | _ | _ | 2 | 8.887 | 50 | 4.725 | 1.200 |
| Halibut | _ | 1.200 | _ | _ | _ | _ | _ | 2 |
| Hake | _ | _ | _ | 2 | 8.887 | 50 | 4.725 | 1.200 |
| Rockfish | _ | _ | _ | _ | 8.887 | 50 | 4.725 | 1.200 |
| Lingcod | _ | 1.200 | 2.095 | _ | 8.887 | _ | _ | 2 |
| Pacific sand lance | _ | _ | _ | _ | 8.887 | _ | _ | 2 |
| Other forage fish | _ | _ | _ | _ | 8.887 | 50 | 4.725 | 1.200 |
| Other fish | _ | 1.200 | 2.095 | 2 | 8.887 | 50 | 4.725 | 1.200 |
| Invertebrates | _ | _ | _ | _ | _ | _ | _ | 1.200 |

**Table A6. List of the vulnerability parameters with the lowest sum of squared differences between model predictions and reference time-series data of biomass and mortalities for the seven Chinook salmon groups included in the model.** Predators are listed in the top row and prey species in the first column.

| **Predator** | **FRGSPS SP** | | | | | | **FRGSPS SU** | | | | | |
| --- | --- | --- | --- | --- | --- | --- | --- | --- | --- | --- | --- | --- |
| **Prey** | *River* | *Smolts* | *Juveniles* | *Marine* | *Returning spawners* | *Escapees* | *River* | *Smolts* | *Juveniles* | *Marine* | *Returning spawners* | *Escapees* |
| Herring *Juveniles* | _ | 2 | 2 | _ | _ | _ | _ | 2 | 2 | _ | _ | _ |
| Herring *Adults* | _ | _ | _ | _ | ∞ | _ | _ | _ | _ | _ | 1 | _ |
| Halibut | _ | _ | _ | _ | _ | _ | _ | _ | _ | _ | _ | _ |
| Hake | _ | _ | _ | _ | _ | _ | _ | _ | _ | _ | _ | _ |
| Rockfish | _ | _ | _ | _ | _ | _ | _ | _ | _ | _ | _ | _ |
| Lingcod | _ | _ | _ | _ | _ | _ | _ | _ | _ | _ | _ | _ |
| Pacific sand lance | _ | _ | _ | _ | ∞ | _ | _ | _ | _ | _ | 1 | _ |
| Other forage fish | _ | _ | _ | ∞ | _ | 2 | _ | _ | _ | ∞ | _ | 2 |
| Other fish | _ | 2 | 2 | ∞ | _ | 2 | _ | 2 | 2 | ∞ | _ | 2 |
| Invertebrates | _ | 2 | 2 | _ | ∞ | _ | _ | 2 | 2 | _ | 1 | _ |
| Zooplankton | 2 | 2 | 2 | _ | _ | _ | 2 | 2 | 2 | _ | _ | _ |

| **Predator** | **FRGSPS FA** | | | | | | **WCVI FA** | | | | | |
| --- | --- | --- | --- | --- | --- | --- | --- | --- | --- | --- | --- | --- |
| **Prey** | *River* | *Smolts* | *Juveniles* | *Marine* | *Returning spawners* | *Escapees* | *River* | *Smolts* | *Juveniles* | *Marine* | *Returning spawners* | *Escapees* |
| Herring *Juveniles* | _ | 2 | 2 | _ | _ | _ | _ | 2 | 2 | _ | _ | _ |
| Herring *Adults* | _ | _ | _ | _ | 1 | _ | _ | _ | _ | _ | ∞ | _ |
| Halibut | _ | _ | _ | _ | _ | _ | _ | _ | _ | _ | _ | _ |
| Hake | _ | _ | _ | _ | _ | _ | _ | _ | _ | _ | _ | _ |
| Rockfish | _ | _ | _ | _ | _ | _ | _ | _ | _ | _ | _ | _ |
| Lingcod | _ | _ | _ | _ | _ | _ | _ | _ | _ | _ | _ | _ |
| Pacific sand lance | _ | _ | _ | _ | 1 | _ | _ | _ | _ | _ | ∞ | _ |
| Other forage fish | _ | _ | _ | ∞ | _ | 2 | _ | _ | _ | ∞ | _ | 2 |
| Other fish | _ | 2 | 2 | ∞ | _ | 2 | _ | 2 | 2 | ∞ | _ | 2 |
| Invertebrates | _ | 2 | 2 | _ | 1 | _ | _ | 2 | 2 | _ | ∞ | _ |
| Zooplankton | 2 | 2 | 2 | _ | _ | _ | 2 | 2 | 2 | _ | _ | _ |

**Table A6. Continued**

| **Predator** | **CRWORC SP** | | | | | | **CRWORC SU** | | | | | |
| --- | --- | --- | --- | --- | --- | --- | --- | --- | --- | --- | --- | --- |
| **Prey** | *River* | *Smolts* | *Juveniles* | *Marine* | *Returning spawners* | *Escapees* | *River* | *Smolts* | *Juveniles* | *Marine* | *Returning spawners* | *Escapees* |
| Herring *Juveniles* | _ | 2 | 2 | _ | _ | _ | _ | 2 | ∞ | _ | _ | _ |
| Herring *Adults* | _ | _ | _ | _ | ∞ | _ | _ | _ | _ | _ | ∞ | _ |
| Halibut | _ | _ | _ | _ | _ | _ | _ | _ | _ | _ | _ | _ |
| Hake | _ | _ | _ | _ | _ | _ | _ | _ | _ | _ | _ | _ |
| Rockfish | _ | _ | _ | _ | _ | _ | _ | _ | _ | _ | _ | _ |
| Lingcod | _ | _ | _ | _ | _ | _ | _ | _ | _ | _ | _ | _ |
| Pacific sand lance | _ | _ | _ | _ | ∞ | _ | _ | _ | _ | _ | ∞ | _ |
| Other forage fish | _ | _ | _ | ∞ | _ | 2 | _ | _ | _ | ∞ | _ | 2 |
| Other fish | _ | 2 | 2 | ∞ | _ | 2 | _ | 2 | ∞ | ∞ | _ | 2 |
| Invertebrates | _ | 2 | 2 | _ | ∞ | _ | _ | 2 | ∞ | _ | ∞ | _ |
| Zooplankton | 2 | 2 | 2 | _ | _ | _ | 2 | 2 | ∞ | _ | _ | _ |

| **Predator** | **CRWORC FA** | | | | | |
| --- | --- | --- | --- | --- | --- | --- |
| **Prey** | *River* | *Smolts* | *Juveniles* | *Marine* | *Returning spawners* | *Escapees* |
| Herring *Juveniles* | _ | 2 | 2 | _ | _ | _ |
| Herring *Adults* | _ | _ | _ | _ | 1 | _ |
| Halibut | _ | _ | _ | _ | _ | _ |
| Hake | _ | _ | _ | _ | _ | _ |
| Rockfish | _ | _ | _ | _ | _ | _ |
| Lingcod | _ | _ | _ | _ | _ | _ |
| Pacific sand lance | _ | _ | _ | _ | 1 | _ |
| Other forage fish | _ | _ | _ | 2 | _ | 2 |
| Other fish | _ | 2 | 2 | 2 | _ | 2 |
| Invertebrates | _ | 2 | 2 | _ | 1 | _ |
| Zooplankton | 2 | 2 | 2 | _ | _ | _ |

**Table A7. List of the vulnerability parameters with the lowest sum of squared differences between model predictions and reference time-series data of biomass and mortalities for the remaining functional groups included in the model.** Predators are listed in the top row and prey species in the first column.

| **Predator** | Coho *River* | Coho *Smolts* | | Coho *Marine* | Coho *Returning spawners* | Coho *Escapees* | Chum *River* | Chum *Smolts* | Chum *Marine* | Chum *Returning spawners* | Chum *Escapees* |
| --- | --- | --- | --- | --- | --- | --- | --- | --- | --- | --- | --- |
| **Prey** |  |  |  |  |  |  |  |  |  |  |  |
| Herring *Juveniles* | _ | | 2 | _ | _ | _ | _ | _ | _ | _ | _ |
| Herring *Adults* | _ | | _ | _ | 1 | _ | _ | _ | _ | _ | _ |
| Halibut | _ | | _ | _ | _ | _ | _ | _ | _ | _ | _ |
| Hake | _ | | _ | _ | _ | _ | _ | _ | _ | _ | _ |
| Rockfish | _ | | _ | _ | _ | _ | _ | _ | _ | _ | _ |
| Lingcod | _ | | _ | _ | _ | _ | _ | _ | _ | _ | _ |
| Pacific sand lance | _ | | _ | _ | 1 | _ | _ | _ | _ | _ | _ |
| Other forage fish | _ | | _ | 2 | _ | 2 | _ | _ | ∞ | 1 | 2 |
| Other fish | _ | | 2 | 2 | _ | 2 | _ | _ | ∞ | 1 | 2 |
| Invertebrates | _ | | _ | _ | 1 | _ | _ | 2 | _ | 1 | _ |
| Zooplankton | 2 | | _ | _ | _ | _ | 2 | 2 | _ | _ | _ |

| **Predator** | Other salmonids | Herring *Juveniles* | Herring *Adults* | Halibut | Hake | Rockfish | Lingcod | Pacific sand lance | Other forage fish | Other fish | Invertebrates | Zooplankton |
| --- | --- | --- | --- | --- | --- | --- | --- | --- | --- | --- | --- | --- |
| **Prey** |  |  |  |  |  |  |  |  |  |  |  |  |
| Herring *Juveniles* |  |  |  |  |  |  | 2 |  |  |  |  |  |
| Herring *Adults* | 1.001 |  |  | 2 | 2 | 2 | 2 |  |  |  |  |  |
| Halibut |  |  |  |  |  |  |  |  |  |  |  |  |
| Hake |  |  |  |  |  |  | 2 |  |  |  |  |  |
| Rockfish |  |  |  | 2 |  |  | 2 |  |  |  |  |  |
| Lingcod |  |  |  |  |  |  |  |  |  |  |  |  |
| Pacific sand lance | 1.001 |  |  | 2 |  | 2 | 2 |  |  |  |  |  |
| Other forage fish |  |  | 3.909 | 2 | 2 | 2 | 2 |  |  |  |  |  |
| Other fish |  |  | 3.909 | 2 | 2 | 2 | 2 | ∞ | 6 | 2 |  |  |
| Invertebrates | 1.001 |  | 3.909 | 2 | 2 | 2 | 2 | ∞ | 6 | 2 | 1 |  |
| Zooplankton | 1.001 | 1 | 3.909 |  |  |  |  |  | 6 | 2 | 1 |  |
| Phytoplankton |  |  |  |  |  |  |  |  |  |  |  | ∞ |

**References (Appendix A)**

[1] Ford JKB, Ellis GM, Durban JW. An assessment of the Potential for Recovery of West Coast Transient Killer Whales Using Coastal Waters of British Columbia. *Can Sci Advis Secr Pac Reg Sci Advis Rep* 2007; 088: 38.

[2] Morin PA, Archer FI, Foote AD, Vilstrup J, Allen EE, Wade P, et al. Complete mitochondrial genome phylogeographic analysis of killer whales (*Orcinus orca*) indicates multiple species. *Genome Res* 2010; 20: 908–916.

[3] Barrett-Lennard LG, Ellis GM. Population Structure and Genetic Variability in Northeastern Pacific Killer Whales: Towards an Assessment of Population Viability. *Fish Ocean Can Pac Biol Stn Nanaimo* 2001; DFO Canadian Science Advisory Secretariat Research Document: 35.

[4] Committee on the Status of Endangered Wildlife in Canada. COSEWIC assessment and update status report on the killer whale, Orcinus orca: Southern resident population, Northern resident population, West Coast transient population, Offshore population, Northwest Atlantic/Eastern Arctic population in Canada. Ottawa: Committee on the Status of Endangered Wildlife in Canada, http://epe.lac-bac.gc.ca/100/200/301/environment_can/cws-scf/cosewic-cosepac/killer_whale-e/CW69-14-564-2009E.pdf (2009, accessed 19 January 2022).

[5] Ford JKB, Stredulinsky EH, Towers JR, Ellis GM. Information in Support of the Identification of Critical Habitat for Transient Killer Whales (*Orcinus orca* ) off the West Coast of Canada. *Can Sci Advis Secr Res Doc* 2012; 155: 50.

[6] Noren DP. Estimated field metabolic rates and prey requirements of resident killer whales. *Mar Mammal Sci* 2011; 27: 60–77.

[7] Bigg MA, Ellis GM, Ford JKB, Balcomb KC. *Killer whales: a study of their identification, genealogy, and natural history in British Columbia and Washington State*. Nanaimo, B.C., Canada: Phantom Press, 1987.

[8] Baird RW, Stacey PJ. Variation in saddle patch pigmentation in populations of killer whales ( *Orcinus orca* ) from British Columbia, Alaska, and Washington State. *Can J Zool* 1988; 66: 2582–2585.

[9] Google Technology Company. Google Earth. *Google Earth*, https://earth.google.com/web/ (2022, accessed 12 June 2023).

[10] Williams TM, Estes JA, Doak DF, Springer AM. killer appetites: assessing the role of predators in ecological communities. *Ecology* 2004; 85: 3373–3384.

[11] Center for Whale Research. The Southern Resident Killer Whale Population. *Orcas // Killer Whales | United States | Center For Whale Research*, https://www.whaleresearch.com/orca-population (2022, accessed 30 January 2020).

[12] Couture F, Oldford G, Christensen V, Walters CJ. Requirements and availability of prey for northeastern pacific southern resident killer whales. *PLOS ONE* 2022; 17: e0270523.

[13] Robeck TR, Willis K, Scarpuzzi MR, O’Brien JK. Comparisons of life-history parameters between free-ranging and captive killer whale ( *Orcinus orca* ) populations for application toward species management. *J Mammal* 2015; 96: 1055–1070.

[14] Ford J, Ellis G. Selective foraging by fish-eating killer whales Orcinus orca in British Columbia. *Mar Ecol Prog Ser* 2006; 316: 185–199.

[15] Olesiuk PF, Bigg MA, Ellis GM. Life history and population dynamics of Resident Killer Whales (*Orcinus orca*) in the coastal waters of British Columbia and Washington State. *Rep Int Whal Comm*.

[16] Krahn MM, Wade PR, Kalinowski ST, Dahlheim ME, Taylor BL. Status Review of Southern Resident Killer Whales (*Orcinus orca*) under the Endangered Species Act. *NOAA Tech Memo NMFS-NWFSC* 2002; 54: 159.

[17] Olesiuk PF, Ellis GM, Ford JKB. Biological cycle and population dynamics of Northern Resident Killer Whale in British Columbia. *Fish Oceans Can* 2005; Canadian Science Advisory Secretariat Research Document: 82.

[18] Ford JKB, Ellis GM, Olesiuk PF. Disponibilité des proies et dynamique des populations : est-ce que la limitation des ressources alimentaires a pu causer les diminutions récentes des épaulards « résidants » (Orcinus orca) en Colombie-Britannique? 2005; 31.

[19] B.C. Minist of Environment. Species Summary:  *Phocoena phocoena* . *B.C. Conservation Data Centre*, https://a100.gov.bc.ca/pub/eswp/reports.do?elcode=AMAGF01010 (1994, accessed 13 June 2023).

[20] B.C. Minist. of Environment. Species Summary:  *Phocoenoides dalli* . *B.C. Conservation Data Centre*, https://a100.gov.bc.ca/pub/eswp/reports.do?elcode=AMAGF02010 (1994, accessed 13 June 2023).

[21] Gaskin D. Status of the Harbour Porpoise,  *Phocoena phocoena* , in Canada. *Can Field Nat* 1992; 106: 36–54.

[22] COSEWIC. COSEWIC assessment and update status report on the harbour porpoise  *Phocoena phocoena*  (Pacific Ocean population) in Canada. *Comm Status Endanger Wildl Can Ott* 2003; 22 pp.

[23] Laake J, Calambokidis J, Osmek S. Survey report for the 1997 aerial surveys fir harbor porpoise and other marine mammals of Oregon, Washington, and British Columbia outside waters. AFSC Processed Report 98-10., National Marine Fisheries Service, Seattle, WA, 1998.

[24] Jefferson TA. *Phocoenoides dalli*. *Mamm Species* 1988; 1.

[25] Morejohn G. The natural history of Dall’s porpoise in the North Pacific Ocean. In: *Behavior of Marine Animals, Current Perspectives in Research*. New York, London: Plenum Press, https://link.springer.com/chapter/10.1007/978-1-4684-2985-5_3 (1979).

[26] Heise KA. Life history parameters of the Pacific white-sided dolphin (*Lagenorhynchus obliquidens*) and its diet and occurrence in the coastal waters of British Columbia. *Thesis Submitt Partial Fulfillment Requir Degree Master Sci Fac Grad Stud Univ Br Columbia* 1996; 106.

[27] Shore V. Pacific White-sided Dolphin. *E-Fauna BC: Electronic Atlas of the Wildlife of British Columbia*, https://linnet.geog.ubc.ca/efauna_SMaps/index_static.html?sciname=Lagenorhynchus%20obliquidens&synonyms=%27none%27&mapservice=efauna_vertebrates (2021, accessed 13 June 2023).

[28] Preikshot D. The influence of geographic scale, climate and trophic dynamics upon North Pacific oceanic ecosystem models. *Thesis Submitted in Partial Fulfillment of the Requirements for the Degree of Doctor of Philosophy, University of British Columbia*, 10.14288/1.0074902 (2007).

[29] Lockyer C, Desportes G, Hansen K, Labberté S, Siebert U. Monitoring growth and energy utilisation of the harbour porpoise (*Phocoena phocoena*) in the human care. *NAMMCO Sci Publ* 2003; 5: 107–120.

[30] MacLeod R, MacLeod CD, Learmonth JA, Jepson PD, Reid RJ, Deaville R, et al. Mass-dependent predation risk and lethal dolphin–porpoise interactions. *Proc R Soc B Biol Sci* 2007; 274: 2587–2593.

[31] Ohizumi H, Miyazaki N. Feeding rate and energy intake of Dall’s porpoise in the northeastern Sea of Japan. *Proc NIPR Symp Polar* 1988; 11: 74–81.

[32] Rechsteiner E, Rosen DAS, Trites AW. Energetic requirements of Pacific white-sided dolphins (*Lagenorhynchus obliquidens*) as predicted by a bioenergetic model. *J Mammal* 2013; 94: 820–832.

[33] Olesiuk PF. Annual prey consumption by harbor seals (*Phoca vitulina*J in the Strait of Georgia, British Columbia. 1993; 25.

[34] Pitcher K, Calkins DG. Biology of the harbor seal,  *Phoca vitulina richardii* , in the Gulf of Alaska. *Environ Assess Alaskan Cont Shelf Final Rep Princ Investig* 1979; 19: 571.

[35] Fisheries and Oceans Canada. Stock Assessment of Pacific Harbour Seals (*Phoca vitulina richardii*) in Canada in 2019. 2022; 10.

[36] Jeffries S, Huber H, Calambokidis J, Laake J. Trends and Status of Harbor Seals in Washington State: 1978-1999. *J Wildl Manag* 2003; 67: 207.

[37] Brown RF, Wright BE, Riemer SD, Laake J. Trends in abundance and current status of harbor seals in Oregon 1977-2003. *Mar Mammal Sci* 2005; 21: 657–670.

[38] Hanan D. Dynamics of abundance and distribution for Pacific harbor seal, phoca vitulina richardsi, on the coast of California. A Dissertation Submitted in Partial Satisfaction of the Requirements for the Degree Doctor of Philisophy in Biology, University of California, 1996.

[39] Olesiuk PF. An assessment of the status of harbour seals (*Phoca vitulina*) in British Columbia. *Fish Oceans Can* 1999; Canadian Stock Assessment Secretariat Research Document: 71.

[40] Olesiuk PF. Recent trends in Abundance of Steller Sea Lions (*Eumetopias jubatus*) in British Columbia. *Fish Ocean Can Pac Biol Stn Nanaimo* 2018; Canadian Science Advisory Secretariat Proceedings Series DFO: 72.

[41] Winship AJ, Trites AW, Calkins DG. Growth in body size of the Steller Sea Lion. *J Mammal* 2001; 82: 20.

[42] Fisheries and Oceans Canada. Trends in Abundance and Distribution of Steller Sea Lions (*Eumetopias Jubatus*) in Canada. *Can Sci Advis Secr Sci Advis Rep* 2021; 035: 8.

[43] Winship AJ, Trites AW, Rosen DAS. A bioenergetic model for estimating the food requirements of Steller sea lions  *Eumetopias jubatus*  in Alaska, USA. *Mar Ecol Prog Ser* 2002; 229: 291–312.

[44] Laake JL, Lowry MS, DeLong RL, Melin SR, Carretta JV. Population growth and status of california sea lions: Status of California Sea Lions. *J Wildl Manag* 2018; 82: 583–595.

[45] Fisheries and Oceans Canada. California Sea Lion Abundance Estimation in Canada, 2020–21. *Can Sci Advis Secr Sci Advis Rep*; 016.

[46] Kastelein RA, Schooneman NM, Vaughan N, Wiepkema PR. Food consumption and growth of California sea lions (*Zalophus californianus*). *Zoo Biol* 2000; 19: 143–159.

[47] McHuron E, Mangel M, Schwarz L, Costa DP. Energy and prey requirements of California sea lions under variable environmental conditions. *Mar Ecol Prog Ser* 2017; 567: 235–247.

[48] Weise M, Harvey J. Temporal variability in ocean climate and California sea lion diet and biomass consumption: implications for fisheries management. *Mar Ecol Prog Ser* 2008; 373: 157–172.

[49] DeLong RL, Melin SR, Laake JL, Morris P, Orr AJ, Harris JD. Age‐ and sex‐specific survival of California sea lions (*Zalophus californianus*) at San Miguel Island, California. *Mar Mammal Sci* 2017; 33: 1097–1125.

[50] NOAA. California sea lion (*Zalophus californianus*): U.S. Stock. *Mar Mammal Stock Assess Rep*.

[51] Manuwal D, Carter H, Zimmerman T, Orthmeyer DL. *Biology and Conservation of the Common Murre in California, Oregon, Washington, and British Columbia. Volume 1: Natural History and Population Trends*. Information and Technology Report USGS/BRD/ITR-2000-0012, 2001.

[52] B.C. Minis. of Environment. Species Summary:  *Uria aalge* . *B.C. Conservation Data Centre*, https://a100.gov.bc.ca/pub/eswp/reports.do?elcode=ABNNN02010 (1996, accessed 15 June 2023).

[53] Gaston AJ, Bertram DF, Boyne AW, Chardine JW, Davoren G, Diamond AW, et al. Changes in Canadian seabird populations and ecology since 1970 in relation to changes in oceanography and food webs. *Environ Rev* 2009; 17: 267–286.

[54] B.C. Minis. of Environment. Species Summary:  *Cerorhinca monocerata* . *B.C. Conservation Data Centre*, https://a100.gov.bc.ca/pub/eswp/reports.do?elcode=ABNNN11010 (1996, accessed 15 June 2023).

[55] Bertram DF, Jones IL, Cooch EG, Knechtel HA, Cooke F. Survival rates of Cassin’s and Rhinoceros Aucklets at Triangle Island, British Columbia. *Ornithol Appl* 2000; 102: 8.

[56] Gabrielsen GW. Energy expenditure of breeding Common Murres. *Occas Pap Can Wildl Serv*; Occas. Pap. Can. Wildl. Serv.

[57] Roth JE, Nur N, Warzybok P, Sydeman WJ. Annual prey consumption of a dominant seabird, the common murre, in the California Current system. *ICES J Mar Sci* 2008; 65: 1046–1056.

[58] Vermeer K, Devito K. Size, Caloric Content, and Association of Prey Fishes in Meals of Nestling Rhinoceros Auklets. *The Murrelet* 1986; 67: 1.

[59] Pacific Salmon Commission, Joint Chinook Technical Committee Report. *2019 Exploitation rate analysis and model calibration*. Joint Technical Committee Report tcchinook-01, Pacific salmon commission, 2021.

[60] Pacific Fishery Management Council. *Stock Abundance Analysis and Environmental Assessment Part 1 for 2022 Ocean Salmon Fishery Regulations*. Preseason Report I, Pacific Fishery Management Council, 7700 NE Ambassador Place, Suite 101, Portland, Oregon 97220-1384., 2022.

[61] Pacific Fishery Management Council. *Review of 2021 Ocean Salmon Fisheries*. Stock Assessment and Fishery Evaluation Document for the Pacifc Coast Salmon Fishery Management Plan, Pacific Fishery Management Council, 7700 NE Ambassador Place, Suite 101, Portland, Oregon 97220-1384., 2022.

[62] Christensen V, Walters CJ, Pauly D, Forrest RE. *Ecopath with Ecosim: User Guide version 6*. Lenfest Ocean Futures Project 2008. 2008.

[63] Heymans JJ, Coll M, Link JS, Mackinson S, Steenbeek J, Walters C, et al. Best practice in Ecopath with Ecosim food-web models for ecosystem-based management. *Ecol Model* 2016; 331: 173–184.

[64] Shelton AO, Satterthwaite WH, Ward EJ, Feist BE, Burke B. Using hierarchical models to estimate stock-specific and seasonal variation in ocean distribution, survivorship, and aggregate abundance of fall run Chinook salmon. *Can J Fish Aquat Sci* 2019; 76: 95–108.

[65] Tucker S, Trudel M, Welch DW, Candy JR, Morris JF, Thiess ME, et al. Life History and Seasonal Stock-Specific Ocean Migration of Juvenile Chinook Salmon. *Trans Am Fish Soc* 2011; 140: 1101–1119.

[66] Ruggerone GT, Irvine JR. Numbers and Biomass of Natural- and Hatchery-Origin Pink Salmon, Chum Salmon, and Sockeye Salmon in the North Pacific Ocean, 1925–2015. *Mar Coast Fish* 2018; 10: 152–168.

[67] Pacific Salmon Commission Joint Chinook Technical Committee Report. Pacific Salmon Commission Chinook Model Base Period Re-Calibration, Volume I: Fisheries. 21.

[68] Froese R, Pauly D. FishBase. *FishBase*, https://www.fishbase.se/search.php (2023, accessed 15 June 2023).

[69] Parker RR. Critical Size and Maximum Yield for Chinook Salmon ( *Oncorhynchus tshawytscha* ). *J Fish Res Board Can* 1960; 17: 199–210.

[70] Pacific States Marine Fisheries Commission. Regional Mark Processing Centre. *Regional Mark Processing Center*, https://www.rmpc.org/ (2022, accessed 15 June 2023).

[71] Sandercock FK. Life history of coho salmon *Oncorhynchus kisutch*). In: *Pacific salmon life histories*. Vancouver, B.C.: University of British Columbia Press, 1991, pp. 396–455.

[72] Beamish RJ, Sweeting RM. Patterns in the recapture rates among release locations of coded-wire tagged juvenile coho salmon in the Strait of Georgia. Document 436, Nanaimo, British Columbia: North Pacific Anadromous Fish Commission, Pacific Biological Station, 1999.

[73] Beamish RJ, Mahnken C, Neville CM. Evidence That Reduced Early Marine Growth is Associated with Lower Marine Survival of Coho Salmon. *Trans Am Fish Soc* 2004; 133: 26–33.

[74] Bradford MJ. Comparative review of Pacific salmon survival rates. *Can J Fish Aquat Sci* 1995; 52: 1327–1338.

[75] Coronado C, Hilborn R. Spatial and Temporal Factors Affecting Survival in Coho and Fall Chinook Salmon in the Pacific Northwest. *Bull Mar Sci* 1998; 62: 17.

[76] Quinn TP, Dickerson BR, Vøllestad LA. Marine survival and distribution patterns of two Puget Sound hatchery populations of coho (*Oncorhynchus kisutch*) and chinook (*Oncorhynchus tshawytscha*) salmon. *Fish Res* 2005; 76: 209–220.

[77] Zimmerman MS, Irvine JR, O’Neill M, Anderson JH, Greene CM, Weinheimer J, et al. Spatial and Temporal Patterns in Smolt Survival of Wild and Hatchery Coho Salmon in the Salish Sea. *Mar Coast Fish* 2015; 7: 116–134.

[78] Fisheries and Oceans Canada. Coho Salmon in Georgia Basin. *Stock Status Rep* 2002; D6-07: 6.

[79] Fisheries and Oceans Canada. 2019 marine survival forecast of southern British Columbia coho. *Fish Ocean Can* 2019; Report of Marine Survival Forecast of Southern British Columbia Coho: 13.

[80] National Oceanic and Atmospheric Administration. Coho Salmon | NOAA Fisheries. *NOAA*, https://www.fisheries.noaa.gov/species/coho-salmon (2019, accessed 30 January 2020).

[81] Grant S, Pestal G. Certification Unit Profile: Fraser River Chum Salmon. *Can Manuscr Rep Fish Aquat Sci* 2009; 2874: vii + 40p.

[82] Quinn TP, Losee JP. Diverse and changing use of the Salish Sea by Pacific salmon, trout, and char. *Can J Fish Aquat Sci* 2022; 79: 1003–1021.

[83] Fisheries and Oceans Canada. *Inner south coast chum salmon*. DFO Science, Stock Status Report D6-09, Nanaimo, B.C., Canada: Pacific Biological Station, https://waves-vagues.dfo-mpo.gc.ca/Library/318397.pdf (1999).

[84] Northwest Power and Conservation Council. Recovering Chum Salmon. *Recovering Chum Salmon*, https://www.nwcouncil.org/news/2017/07/13/recovering-chum-salmon/ (2017, accessed 15 June 2023).

[85] Pacific Salmon Commission Joint Chum Technical Committee Report. *Final 1985 post season summary report*. Report TCCHUM 87–4, 1987.

[86] Pacific Salmon Foundation. Salmon Facts. *Pacific Salmon Foundation*, https://psf.ca/learn/salmon-facts/ (2023, accessed 15 June 2023).

[87] Parker RR. Estimations of Ocean Mortality Rates for Pacific Salmon ( *Oncorhynchus* ). *J Fish Res Board Can* 1962; 19: 561–589.

[88] Healey MC. Timing and Relative Intensity of Size-Selective Mortality of Juvenile Chum Salmon ( *Oncorhynchus keta* ) During Early Sea Life. *Can J Fish Aquat Sci* 1982; 39: 952–957.

[89] Duffy EJ, Beauchamp DA. Rapid growth in the early marine period improves the marine survival of Chinook salmon (*Oncorhynchus tshawytscha*) in Puget Sound, Washington. *Can J Fish Aquat Sci* 2011; 68: 232–240.

[90] North Pacific Anadromous Fish Commission. NPAFC Statistics: Pacific Salmonid Catch and Hatchery Release Data, https://npafc.org/statistics/ (2022, accessed 15 June 2023).

[91] Aydin KY, McFarlane GA, King JR, Megrey BA. The BASS/MODEL report on trophic models of the subarctic Pacific basin ecosystems. *PICES SCI Rept* 2003; 25: 93 pp.

[92] Schweigert JF, Boldt JL, Flostrand L, Cleary JS. A review of factors limiting recovery of Pacific herring stocks in Canada. *ICES J Mar Sci* 2010; 67: 1903–1913.

[93] Surma S, Pitcher TJ, Kumar R, Varkey D, Pakhomov EA, Lam ME, et al. Herring supports Northeast Pacific predators and fisheries: Insights from ecosystem modelling and management strategy evaluation. *PLOS ONE* 2018; 13: e0196307.

[94] Lance M, Chang W, Jeffries S, Pearson SF, Acevedo-Gutiérrez A. Harbor seal diet in northern Puget Sound: implications for the recovery of depressed fish stocks. *Mar Ecol Prog Ser* 2012; 464: 257–271.

[95] Bromaghin JF, Lance MM, Elliott EW, Jeffries SJ, Acevedo-Gutiérrez A, Kennish JM. New insights into the diets of harbor seals (*Phoca vitulina*) in the Salish Sea revealed by analysis of fatty acid signatures. *Fish Bull*; 111. Epub ahead of print 2013. DOI: 10.7755/FB.111.1.2.

[96] Bishop MA, Green SP. Predation on Pacific herring (*Clupea pallasi*) spawn by birds in Prince William Sound, Alaska: *Predation on Pacific herring spawn*. *Fish Oceanogr* 2001; 10: 149–158.

[97] Buckley T, Livingston P. Geographic variation in the diet of Pacific hake. *Calif Coop Ocean Fish Investig Rep*; 38.

[98] Livingston P. Importance of predation by groundfish, marine mammals and birds on walleye pollock Theragra chalcogramma and Pacific herring Clupea pallasi in the eastern Bering Sea. *Mar Ecol Prog Ser* 1993; 102: 205–215.

[99] Beamish RJ, Neville C, Sweeting R, Lange K. The Synchronous Failure of Juvenile Pacific Salmon and Herring Production in the Strait of Georgia in 2007 and the Poor Return of Sockeye Salmon to the Fraser River in 2009. *Mar Coast Fish* 2012; 4: 403–414.

[100] McMillan CJ. How important are herring to humpback whales? The role of herring in meeting the energetic requirements of humpback whales in a British Columbian feeding ground. *Res Proj Submitt Partial Fulfillment Requir Degree Master Resour Manag - Simon Fraser Univ*; Report 599.

[101] Duffy EJ, Beauchamp DA, Sweeting RM, Beamish RJ, Brennan JS. Ontogenetic Diet Shifts of Juvenile Chinook Salmon in Nearshore and Offshore Habitats of Puget Sound. *Trans Am Fish Soc* 2010; 139: 803–823.

[102] Taylor. Life history and present status of British Columbia herring stocks. *Fish Res Board Can*; Bulletin No. 143.

[103] Thompson SA, Sydeman WJ, Thayer JA, Weinstein A, Krieger KL. Trends in the Pacific Herring ( *Clupea pallasi* ) metapopulation in the California current ecosystem. 2017; 58: 18.

[104] Fisheries and Oceans Canada. Stock Assessment and Management Advice for BC Pacific Herring: 2016 Status and 2017 Forecast. *Can Sci Advis Secr Pac Reg Sci Response* 2016; 052: 56.

[105] Fisheries and Oceans Canada. Stock status update with application of management procedures for Pacific Herring (Clupea pallasii) in British Columbia: Status in 2021 and forecast for 2022. *Can Sci Advis Secr Sci Response*; 039.

[106] Sandell T, Lindquist A, Dionne P, Lowry D. 2016 Washington State Herring Stock Status Report. 2016; 90.

[107] Hanson MB, Baird RW, Ford JKB, Hempelmann-Halos J, Van Doornik DM, Candy JR, et al. Species and stock identification of prey consumed by endangered Southern resident killer whales in their summer range. *Endanger Species Res* 2010; 11: 69–82.

[108] Hanson MB, Emmons CK, Ford MJ, Everett M, Parsons K, Park LK, et al. Endangered predators and endangered prey: Seasonal diet of Southern Resident killer whales. *PLOS ONE* 2021; 16: e0247031.

[109] Fisheries and Oceans Canada. *Lingcod stock assessment and yiled advice for outside stocks in British Columbia*. Science Advisory Report 0.51, 2011.

[110] NOAA Fisheries. Pacific Halibut. *NOAA*, https://www.fisheries.noaa.gov/species/pacific-halibut (2022, accessed 15 June 2023).

[111] Sullivan P, Parma A, Clark W. Scientific Report. *Int Pac Halibut Comm* 1999; Scientific Report 79: 84.

[112] Haggarty D. Rockfish conservation areas in B.C.: Our current state of knowledge. Epub ahead of print 2013. DOI: 10.13140/RG.2.1.1681.4485.

[113] Fiscus CH, Baines GA. Food and Feeding Behavior of Steller and California Sea Lions. *J Mammal* 1966; 47: 195–200.

[114] Dorn MW, Saunders MW, Wilson CD, Cooke K, Kieser R, Wilkins ME. Status of the coastal Pacific hake/whiting stock in U.S. and Canada in 1998. *Nadian Stock Assess Secr Res Doc*; 99/90.

[115] Healey 1991. Life history of Chinook salmon ( *Oncorhynchus tshawytscha* ). In: *Pacific Salmon Life Histories*. University of British Columbia Press, Vancouver B.C, 1991.

[116] Alaska Department for Fish and Game. Pacific sand lance (*Ammodytes hexapterus*).

[117] Christensen V, Walters CJ. Ecopath with Ecosim: methods, capabilities and limitations. *Ecol Model* 2004; 172: 109–139.

[118] Beamish RJ, McFarlane GA, Neville CM, Pearsall I. Changes in the Strait of Georgia ECOPTH model needed to balance the abrupt increases in productivity that occured in 2000. *PICES Sci Rept* 2001; 17: 5–9.

[119] Mackas DL. Seasonal Cycle of Zooplankton off Southwestern British Columbia: 1979–89. *Can J Fish Aquat Sci* 1992; 49: 903–921.

[120] Fulton JM, Arai N, Mason JC. Euphausiids, coelenterates, ctenophores and other zooplankton from the Canadian Pacific coast ichthyoplankton survey. *Can Tech Rep Fish Aquat Sci* 1982; 1125: 75 pp.

[121] Iguchi N, Ikeda T. Production, metabolsim, and P:B ratio of *Euphausia pacifica* (Crustacea: Euphausiacea) in Toyama Bay, southern Japan Sea. *Plankton Biol Ecol* 1999; 46: 68–74.

[122] Pauly D, Christensen V. Mass-Balance Models of North-eastern Pacific Ecosystems. Fisheries Centre Research Reports 4, The University of British Columbia, Vancouver, Canada: The Fisheries Centre, 1996.

[123] Nyblade CF. The Strait of Juan de Fuca intertidal and subtidal benthos, 2nd annual report.

[124] Robinson CLK, Ware DM. Modelling Pelagic Fish and Plankton Trophodynamics off Southwestern Vancouver Island, British Columbia. *Can J Fish Aquat Sci* 1994; 51: 1737–1751.

[125] Ford JKB, Stedulinsky E, Towers J, Ellis GM. Information in Support of the Identification of Critical Habitat for Transient Killer Whales (*Orcinus orca*) off the West Coast of Canada. *Can Sci Advis Secr Sci Advis Rep* 2013; 025: 14.

[126] Ternullo R, Black N. Predation behavior of transient killer whales in Monterey Bay, California.

[127] Ford MJ, Hempelmann J, Hanson MB, Ayres KL, Baird RW, Emmons CK, et al. Estimation of a Killer Whale (*Orcinus orca*) Population’s Diet Using Sequencing Analysis of DNA from Feces. *PLOS ONE* 2016; 11: e0144956.

[128] Williams R, Krkošek M, Ashe E, Branch TA, Clark S, Hammond PS, et al. Competing Conservation Objectives for Predators and Prey: Estimating Killer Whale Prey Requirements for Chinook Salmon. *PLoS ONE* 2011; 6: e26738.

[129] Ford JKB, Ellis G, Barrett-Lennard LG, Morton AB, Palm RS, Balcomb KC. Dietary specialization in two sympatric populations of killer whales (*Orcinus orca*) in coastal British Columbia and adjacent waters. *Can J Zool* 1998; 76: 1456–1471.

[130] Fisheries and Oceans Canada. Recovery Strategy for the Northern and Southern Resident Killer Whales (*Orcinus orca*) in Canada. Species at Risk Act Recovery Strategy Series, Fisheries&Oceans Canada, Ottawa, x + 84 pp.

[131] Thomas AC, Nelson BW, Lance MM, Deagle BE, Trites AW. Harbour seals target juvenile salmon of conservation concern. *Can J Fish Aquat Sci* 2017; 74: 907–921.

[132] Thomas AC, Deagle B, Nordstrom C, Majewski S, Nelson BW, Acevedo-Gutiérrez A, et al. Data on the diets of Salish Sea harbour seals from DNA metabarcoding. *Sci Data* 2022; 9: 68.

[133] Huber HR, Jeffries SJ, Brown RF, Delong RL, Vanblaricom G. Correcting aerial survey counts of harbor seals in Washington and Oregon. *Mar Mammal Sci* 2001; 17: 276–293.

[134] Debertin AJ, Irvine JR, Holt CA, Oka G, Trudel M. Marine growth patterns of southern British Columbia chum salmon explained by interactions between density-dependent competition and changing climate. *Can J Fish Aquat Sci* 2017; 74: 1077–1087.

[135] Chasco B, Kaplan IC, Thomas A, Acevedo-Gutiérrez A, Noren D, Ford MJ, et al. Estimates of Chinook salmon consumption in Washington State inland waters by four marine mammal predators from 1970 to 2015. *Can J Fish Aquat Sci* 2017; 74: 1173–1194.

[136] Lance M, Thompson C. Overlap in diets and foraging of common murres (*Uria aakge*) and rhinoceros aucklets (*Cerorhinca monocerata*) after the breeding season. *Ornithology* 2005; 122: 15.

[137] Ainley DG, Spear LB, Allen SG, Ribic CA. Temporal and Spatial Patterns in the Diet of the Common Murre in California Waters. *The Condor* 1996; 98: 691–705.

[138] Lance M, Pearson S. Assembling and Assessing Seabird Diet Information in the Salish Sea. *Wash Dep Fish Wildl Wildl Res Div Olymp Wash* 2012; 17.

[139] Nichol LM, Hall AM, Ellis GM, Stredulinsky E, Boogaards M, Ford JKB. Dietary overlap and niche partitioning of sympatric harbour porpoises and Dall’s porpoises in the Salish Sea. *Prog Oceanogr* 2013; 115: 202–210.

[140] NOAA Fisheries. Harbor Porpoise. *NOAA*, https://www.fisheries.noaa.gov/species/harbor-porpoise (2022, accessed 15 June 2023).

[141] Columbia River Fish and Wildlive Conservation Office. Chinook salmon *Oncorhyncus tshawytscha*, https://www.fws.gov/sites/default/files/documents/Salmon%20Factsheet%20and%20Activities.pdf (2023).

[142] Brodeur RD, Daly EA, Benkwitt CE, Morgan CA, Emmett RL. Catching the prey: Sampling juvenile fish and invertebrate prey fields of juvenile coho and Chinook salmon during their early marine residence. *Fish Res* 2011; 108: 65–73.

[143] Beacham TD. Type, Quantity, and Size of food of Pacific salmon in the Strait of Juan de Fuca (British Columbia). *Fish Bull* 1986; 84: 14.

[144] Zahner VR. Strategies for coexisting: juvenile pink and chum salmon diets and interactions in a challenging section of coastal migration. A Thesis Submitted in Partial Fulfillment of the Requirements for the Degree of Master of Science in the Faculty Graduate and Postdoctoral Studies, University of British Columbia, 2021.

[145] Azuma T. Diel Feeding Habits of Sockeye and Chum Salmon in the Bering Sea during the Summer. *Nippon Suisan Gakkaishi* 1992; 58: 2019–2025.

[146] Davis NCD. Feeding Ecology of Pacific Salmon (Oncorhynchus spp.) in the Central North Pacific Ocean and Central Bering Sea, 1991-2000. Thesis (Doctoral), Hokkaido University, https://doi.org/10.14943/doctoral.r6113 (2003, accessed 15 June 2023).

[147] Sakai O, Yamamura O, Sakurai Y, Azumaya T. Temporal variation in chum salmon, Oncorhynchus keta, diets in the central Bering Sea in summer and early autumn. *Environ Biol Fishes* 2012; 93: 319–331.

[148] NOAA Fisheries. Pacific Herring, https://www.fisheries.noaa.gov/species/pacific-herring (2023, accessed 15 June 2023).

[149] Alaska Department of Fish and Game. Pacific Halibut Species Profile. *Alaska Department for Fish and Game*, https://www.adfg.alaska.gov/index.cfm?adfg=halibut.main (2023, accessed 15 June 2023).

[150] Pallson W, Tsou T, Bargmann G, Buckley RM, West JE, Mills ML, et al. The Biology and Assessment of Rockfishes in Puget Sound. *Wash Dep Fish Wildl*; Fish Management Division, Fish Program.

[151] Murie DJ. Comparative feeding ecology of two sympatric rockfish congeners, *Sebastes caurinus* (copper rockfish) and *S. maliger* (quillback rockfish). *Mar Biol* 1995; 124: 341–353.

[152] Beaudreau A, Essington T. Spatial, Temporal, and Ontogenetic Patterns of Predation on Rockfishes by Lingcod. *Trans Am Fish Soc* 2007; 136: 1438–1452.

[153] Fisheries and Oceans Canada. Lingcod, https://www.dfo-mpo.gc.ca/species-especes/profiles-profils/lingcod-morue-lingue-eng.html (2018, accessed 15 June 2023).

[154] Hipfner JM, Galbraith M. Diet of the Pacific Sand Lance (*Ammodytes hexapterus*) in the Salish Sea, British Columbia, in the 1960s. *Can Field-Nat* 2014; 128: 57.

[155] Winberg GG. Rate of Metabolism and Food Requirements of Fishes. *Copeia* 1962; 2: 475–476.

[156] Towers JR, Canada, Department of Fisheries and Oceans. Photo-identification catalogue, population status, and distribution of Bigg’s killer whales known from coastal waters of British Columbia, Canada, http://publications.gc.ca/collections/collection_2019/mpo-dfo/Fs97-6-3311-eng.pdf (2019, accessed 19 January 2022).

[157] Shields MW, Hysong-Shimazu S, Shields JC, Woodruff J. Increased presence of mammal-eating killer whales in the Salish Sea with implications for predator-prey dynamics. *PeerJ* 2018; 6: e6062.

[158] Malthus TR. An essay on the principle of population.

[159] Towers JR. Photo-identification catalogue and status of the northern resident killer whale population in 2014. Pacific Biological Station, Nanaimo: Fisheries and Oceans Canada, http://www.deslibris.ca/ID/10050075 (2015, accessed 22 March 2019).

[160] Fisheries and Oceans Canada. Population status update for the Northern Resident Killer Whale *(Orcinus orca)* in 2021. Canadian Science Advisory Secretariat Science Response 027, https://www.google.com/url?sa=i&rct=j&q=&esrc=s&source=web&cd=&ved=0CAIQw7AJahcKEwioj7n8tMb_AhUAAAAAHQAAAAAQAw&url=https%3A%2F%2Fwaves-vagues.dfo-mpo.gc.ca%2Flibrary-bibliotheque%2F41076680.pdf&psig=AOvVaw2bABfCcIPNGy4vRrtct2Vc&ust=1686957620387597 (2022).

[161] Fisheries and Oceans Canada. Population assessment of Pacific Harbour Seal (*Phica vitulina richardsi*). *Can Sci Advis Secr Pac Reg Sci Rep*; 011.

[162] Melin SR, Delong RL, Thomason JR, Vanblaricom GR. Attendance patterns of California sea lion (*Zalophus californianus*) females and pups during the non-breeding season at San Miguel Island. *Mar Mammal Sci* 2000; 16: 169–185.

[163] Pacific Salmon Commission. Final 1989 post season summary report. Report TCCHUM 91–1, 1991.

[164] Pacific Salmon Commission. Final 1993 post season summary report. Report TCCHUM 96–1, 1996.

[165] Pacific Salmon Commission. 2009 post season summary report. Report TCCHUM 11–1, 2011.

[166] Pacific Salmon Commission. 2018 post season summary report. Report TCCHUM, 2022.
