# Supplementary material for: The combined effects of predation, fishing, and ocean productivity on salmon species targeted by marine mammals in the northeast Pacific
Source: PLoS One. 2024 Mar 14;19(3):e0296358. doi: 10.1371/journal.pone.0296358 (PMC10939214; doi:10.1371/journal.pone.0296358)
Supplement: S2 Appendix — (DOCX) [file pone.0296358.s002.docx]

**Supporting information S2 - Appendix B**

***Additional results***


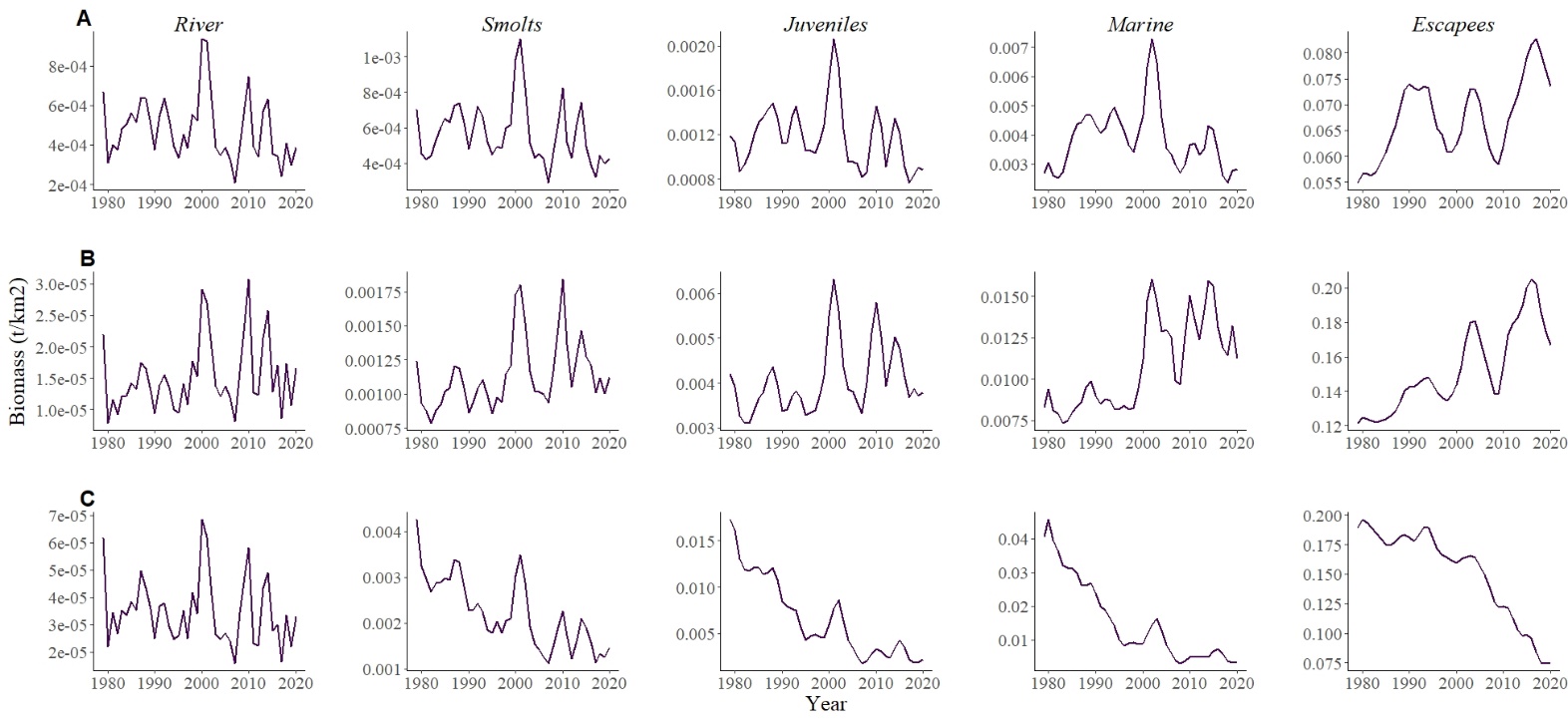


**Figure B1.** **Predicted changes in biomass of the three Chinook salmon functional groups originating from the Salish Sea: FRGSPS SP (A), FRGSPS SU (B), and FRGSPS FA (C) from 1979 to 2020. Results are presented for the stanzas River, Smolts, Juveniles, Marine, and Escapees.**


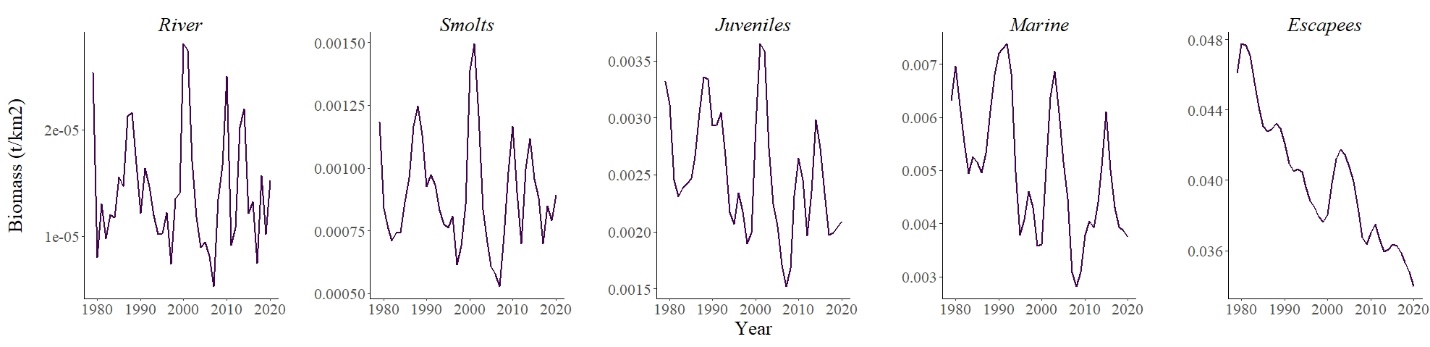


**Figure B2. Predicted changes in biomass of the Chinook salmon functional groups originating from the West Coast of Vancouver Island (WCVI) from 1979 to 2020. Results are presented for the stanzas River, Smolts, Juveniles, Marine, and Escapees.**


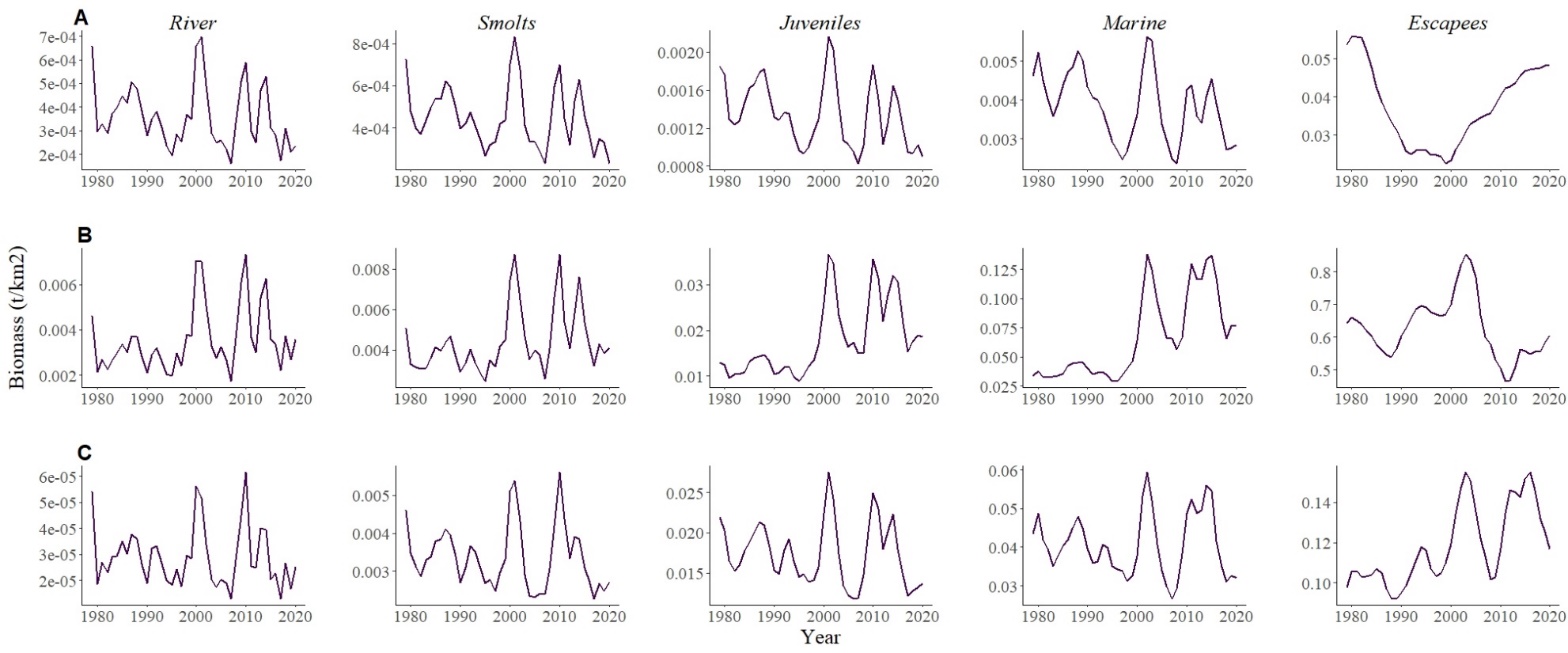


**Figure B3.** **Predicted changes in biomass of the three Chinook salmon functional groups originating from the Columbia River and the Washington, Oregon, and California Coasts: CRWORC SP (A), CRWORC SU (B), and CRWORC FA (C) from 1979 to 2020.** Results are presented for the stanzas River, Smolts, Juveniles, Marine, and Escapees.


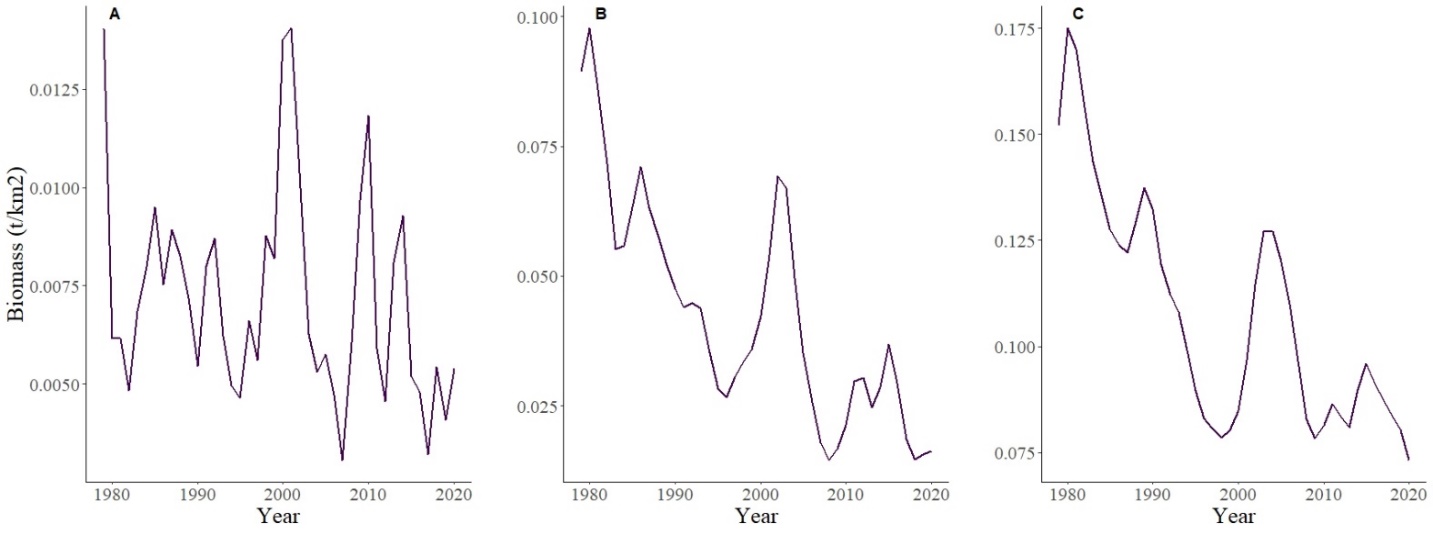


**Figure B4.** **Predicted changes in biomass of the three coho salmon stanzas: River (A), Marine (B), and Escapees (C) from 1979 to 2020.**


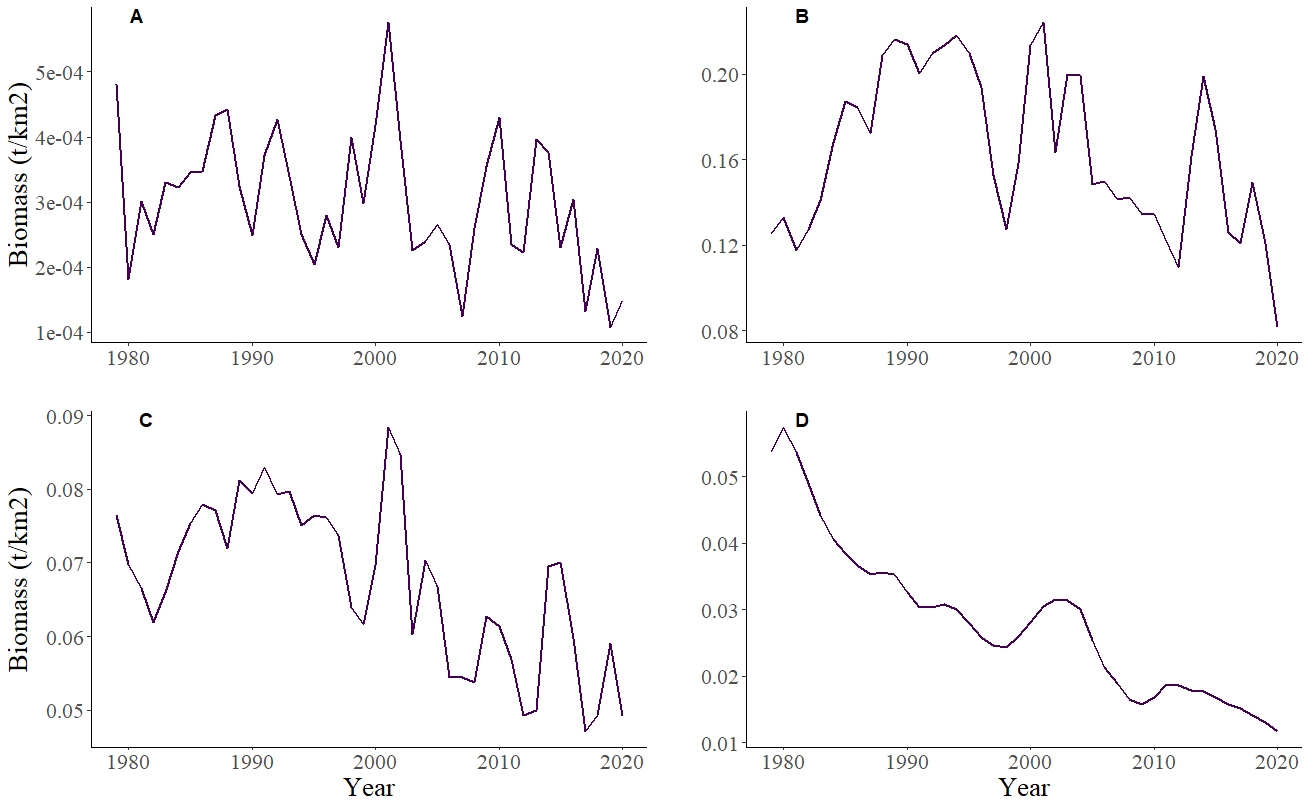


**Figure B5.** **Predicted changes in biomass of the four chum salmon stanzas: River (A), Marine (B), Returning spawners (C), and Escapees (D) from 1979 to 2020.**


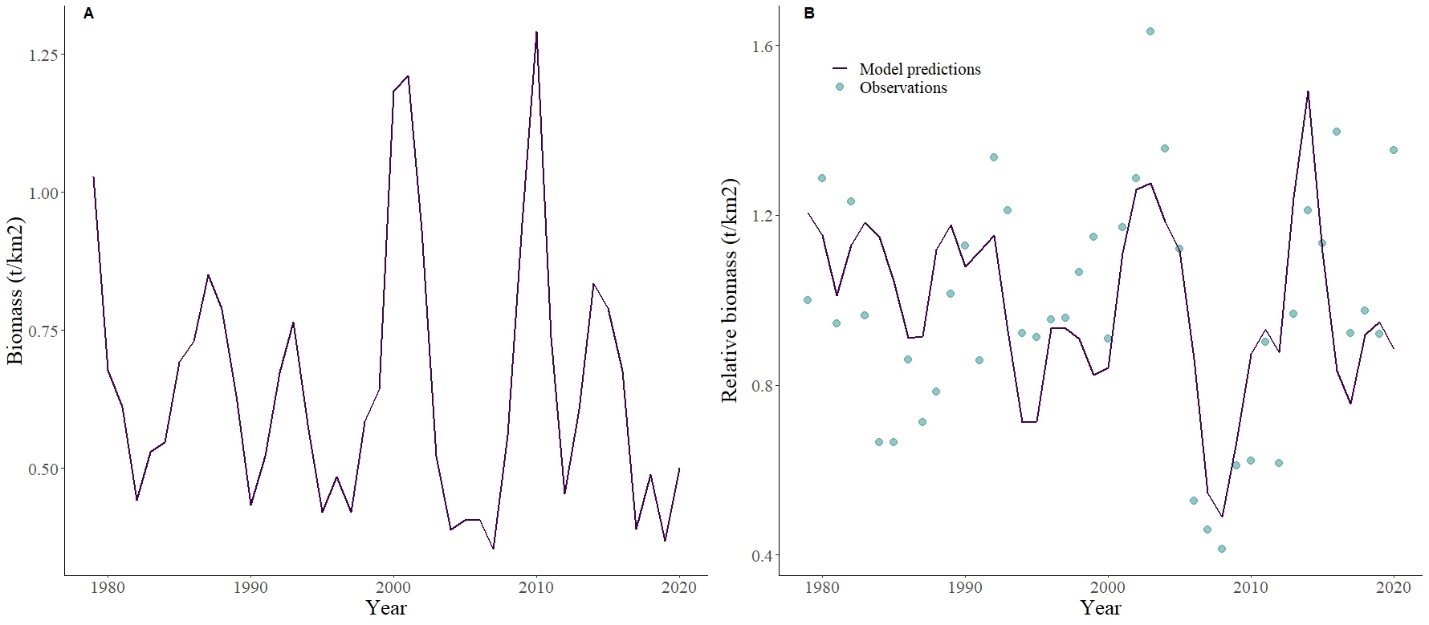


**Figure B6.** **Predicted changes in biomass of the Herring Juveniles stanza (A), and comparison of changes in herring Adults relative predicted biomass (line) versus biomass estimates extracted from literature (points) between1979 and 2020 (B).**


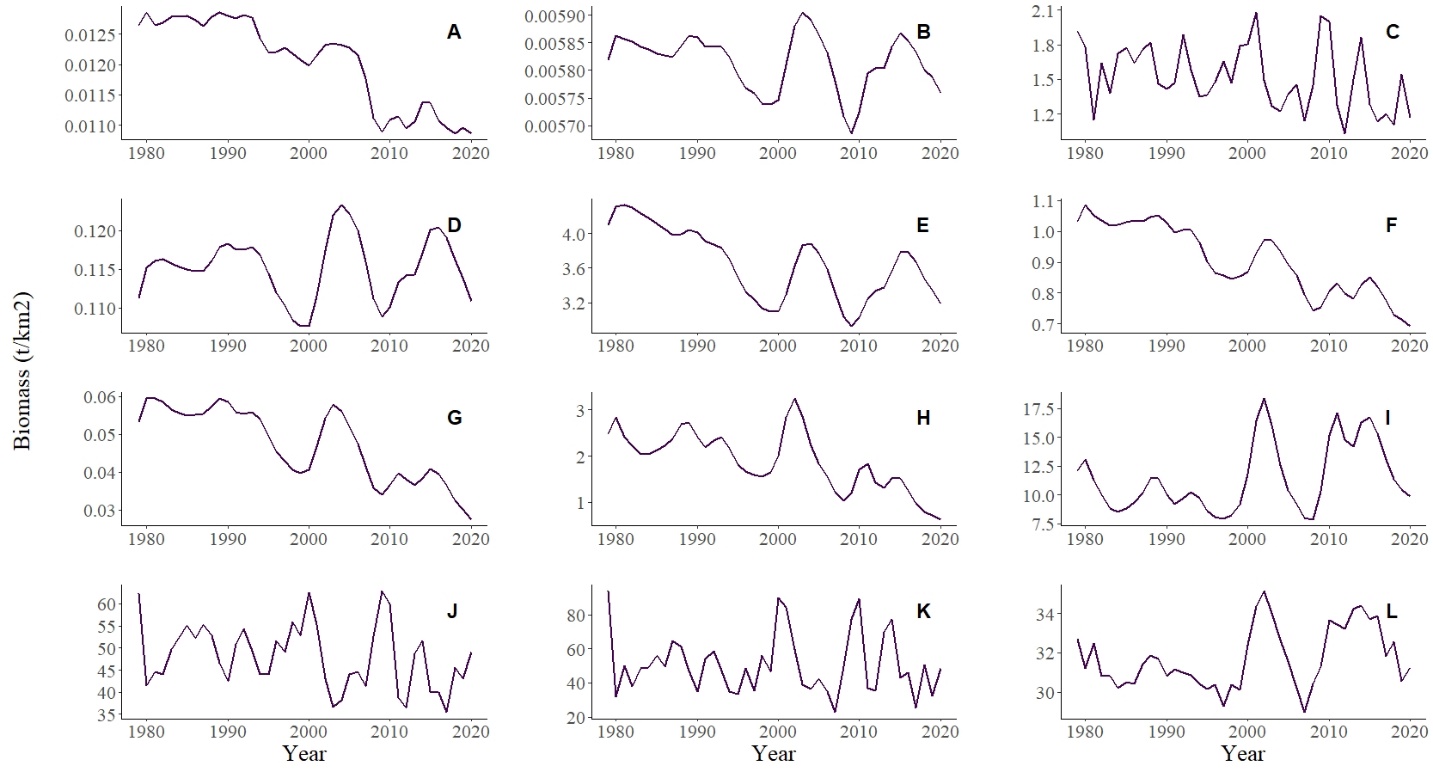


**Figure B7.** **Predicted changes in biomass of Dolphins and porpoises(A), Seabirds (B), Other salmonids (C), Halibut (D), Hake (E), Rockfish (F), Lingcod (G), Sand lance (H), Other forage fish (I), Invertebrates (J), Zooplankton (K), and Phytoplankton (L) between 1979 to 2020.**
